# Modeling Complex Ligands for High Oxidation State Catalysis: Titanium Hydroamination with Unsymmetrical Ligands

**DOI:** 10.1021/acscatal.3c05658

**Published:** 2024-03-29

**Authors:** Zhilin Hou, Rashmi Jena, Tanner J. McDaniel, Brennan S. Billow, Seokjoo Lee, Hannah I. Barr, Aaron L. Odom

**Affiliations:** Department of Chemistry, Michigan State University, 578 S. Shaw Ln, East Lansing, Michigan 48824, United States

**Keywords:** titanium, hydroamination, design, optimization, mechanism

## Abstract

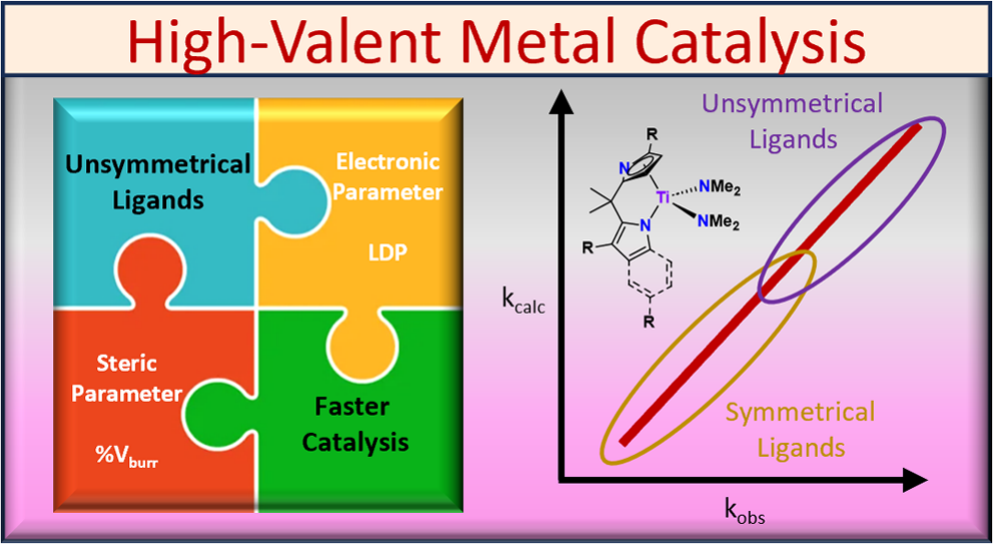

A method for modeling high oxidation state catalysts is used on precatalysts with unsymmetrical and symmetrical bidentate ligands to get a more detailed understanding of how changes to ancillary ligands affect the hydroamination of alkynes catalyzed by titanium. To model the electronic donor ability, the ligand donor parameter (LDP) was used, and to model the steric effects, percent buried volume (% *V*_bur_) was employed. For the modeling study, 7 previously unpublished unsymmetrical Ti(XX′)(NMe_2_)_2_ precatalysts were prepared, where XX′ is a chelating ligand with pyrrolyl/indolyl linkages. The rates of these unsymmetrical and 10 previously reported symmetrical precatalysts were used with the model *k*_obs_ = *a* + *b*(LDP)_1_ + *c*(LDP)_2_ + *d*(% *V*_bur_)_1_ + *e*(% *V*_bur_)_2_, where *a*–*e* were found through least-squares refinement. The model suggests that (1) the two attachment points of the bidentate ligand XX′ are in different environments on the metal (e.g., axial and equatorial in a trigonal bipyramidal or square pyramidal structure), (2) the position of the unsymmetrical ligand on the metal is determined by the electronics of the ligand rather than the sterics, and (3) that one side of the chelating ligand’s electronics strongly influences the rate, while the other side’s sterics more strongly influences the rate. From these studies, we were able to generate catalysts fitting to this model with rate constants larger than the fastest symmetrical catalyst tested.

## Introduction

Catalysis is estimated to contribute to 35% of the global gross domestic product, where it influences the four largest sectors of the world economy: petroleum, power, chemicals, and food.^1,2^ Alwin Mittasch, who helped Carl Bosch develop the industrial process for ammonia production, said, “Chemistry without catalysis would be a sword without a handle, a light without brilliance, a bell without sound.”^1^ Catalysts may be optimized along a large number of vectors including scope, efficiency, selectivity, energy efficiency, and environmental impact;^3^ consequently, general methods for efficient optimization of catalysts are critical to the development of new chemical processes.

While some random screening of catalyst architecture is a necessity, methods for relating the ancillary ligand structure to the properties that may affect reactivity, such as sterics and donor ability, are invaluable tools for catalyst optimization. Tolman and others developed methods for phosphine (L) parametrization, for example, that enable modeling of how phosphine ligand structure affects reactivity.^4^ This methodology was dependent upon experimental methods for determining phosphine donor ability that used carbon monoxide stretching frequencies on nickel complexes, LNi(CO)_3_, to assign a numerical value to ligand donation.^4−6^ However, as effective as this strategy has been for low oxidation state catalyst development, the method is not applicable to high oxidation state metals.^7^

Early metals in high oxidation states are of tremendous importance in catalysis. For example, they are extensively used in bulk chemical production, like olefin polymerization.^8,9^ In addition, important specialty chemical applications use metals in high oxidation states like titanium(IV) in Sharpless asymmetric epoxidation.^10−12^ Further, Schrock and co-workers have published extensively on group-6 d^0^-catalyzed olefin metathesis, where the systems offer unique reactivity.^13,14^

**Scheme 1:**
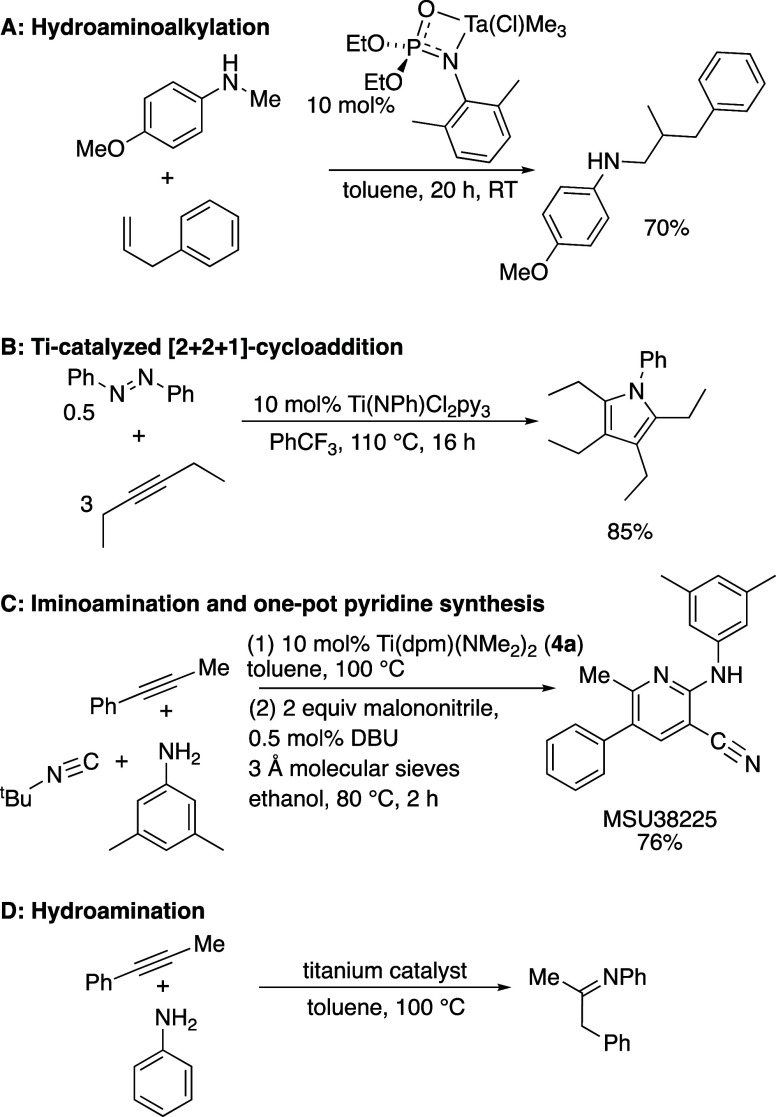
Some Recent Examples of High Oxidation State Transition Metal Reactions and the Reaction Investigated Here, Hydroamination; (A) Hydroaminoalkylation of Alkenes by Schafer and Co-workers;^15^ (B) Titanium Redox Catalysis by Tonks and Co-workers;^16^ (C) One-Pot Synthesis of a 2-Amino-3-cyanopyridines via Alkyne Iminoamination by Odom and Co-workers;^17,18^ (D) Alkyne Hydroamination Test Reaction Used in This Study

The processes catalyzed and mediated by high oxidation state (often d^0^ metals) have undergone extensive development recently, and a few examples are shown in Scheme 1: (A) hydroaminoalkylation of alkenes using Ta(V),^15^ (B) multicomponent redox chemistry to generate pyrroles,^16^ and (C) one-pot synthesis of heterocycles using alkyne iminoamination.^17,19−24^ For example, titanium-catalyzed iminoamination has been used to generate a variety of different heterocycles with biological activity, including a new class of NRF2 inhibitors based on MSU38225^18^ (Scheme 1) and proteasome inhibitors.^25^

For this study, we chose to examine modeling methods for a well-known reaction type, alkyne hydroamination (D, Scheme 1). The alkyne substrate used was 1-phenylpropyne, which gives largely one regioisomer of the product with most catalysts and aniline. The reaction is typically clean and high yielding and was followed by proton nuclear magnetic resonance (^1^H NMR) spectroscopy (vide infra).

To understand and quantify the donor abilities of a host of common anionic ligands for high oxidation state metals, Odom and co-workers developed an experimental method based on a chromium(VI) nitride system, NCr(N^*i*^Pr_2_)_2_X, where X = the ligand being interrogated.^7,26,27^ The system is readily accessed and is synthetically very versatile. The procedures for estimating the barriers to diisopropylamide rotation via ^1^H NMR have been published and discussed on several occasions in the literature.^7,26−28^ The resulting donor parameters for X, ligand donor parameters (LDPs), have been correlated to Hammett parameters for aryloxide ligands, angular overlap model parameters, and carbon NMR^(13^C NMR) data for W(VI) species.^7^ In addition, the same data has been used in combination with a steric parameter (percent buried volume, % *V*_bur_) to model simple hydroamination catalysis by titanium using a 3-parameter model.^28^

**Chart 1:**
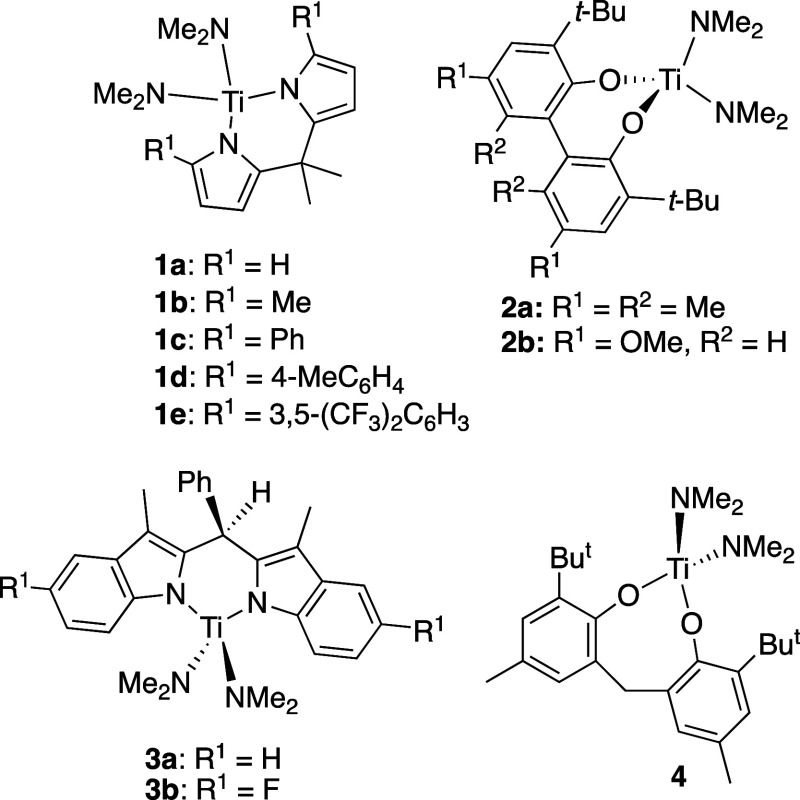
Symmetrical Precatalysts Investigated for the Previous Study^28^ a

In Chart 1 are the titanium complexes with symmetrical ligands that were previously reported as catalysts for alkyne hydroamination and used in the previous study.^28^ The precatalysts with two pyrrolyl ligands generally are η^5^,η^1^ in the solid state (vide infra) but are shown as η^1^,η^1^ to make them easier to visualize. The barriers for exchange between the two sides are low,^29^ and the key step in the catalysis seems to involve the η^1^,η^1^-ligand.^28^

a All ligands shown as η^1^ for clarity but may not be in the solid-state structures.

**Chart 2:**
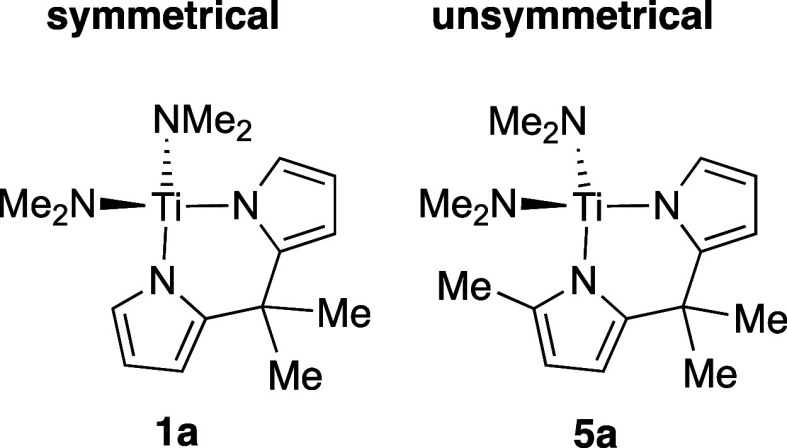
Homogeneous Titanium Catalysts with Bidentate Ancillary Ligands May Be Classified Generally into Symmetrical with ∼*C*_2_ Symmetric Systems with Equivalent Donors (e.g., **1a**) and Unsymmetrical Systems Having Different Donors on Either Side of the Chelate (e.g., **5a**)

Here, we expand this modeling method to more complex unsymmetrical ligand sets and publish an effective model for hydroamination across many types of ancillary ligands for titanium, including both symmetrical and unsymmetrical bidentate ligands (Chart 2). For the purpose of this study, “symmetrical” is being used to describe catalysts bearing ancillary ligands with approximate *C*_2*v*_ symmetry, and “unsymmetrical” ligands are those with different attachment ligands, e.g., indolyl and pyrrolyl, to the metal. For the modeling, the precatalysts with symmetrical ancillary ligands (Chart 1) and new complexes containing unsymmetrical ligands were used in the fit to give a much more detailed picture of how ancillary ligands may influence reaction rate.

While carrying out this study, we were able to design, synthesize, and test catalysts that are faster than any we have previously prepared. In the process, some experimentally determined details regarding the nature of the key transition state were discerned.

## Results and Discussion

### Synthesis and Characterization of Precatalysts

**Scheme 2:**
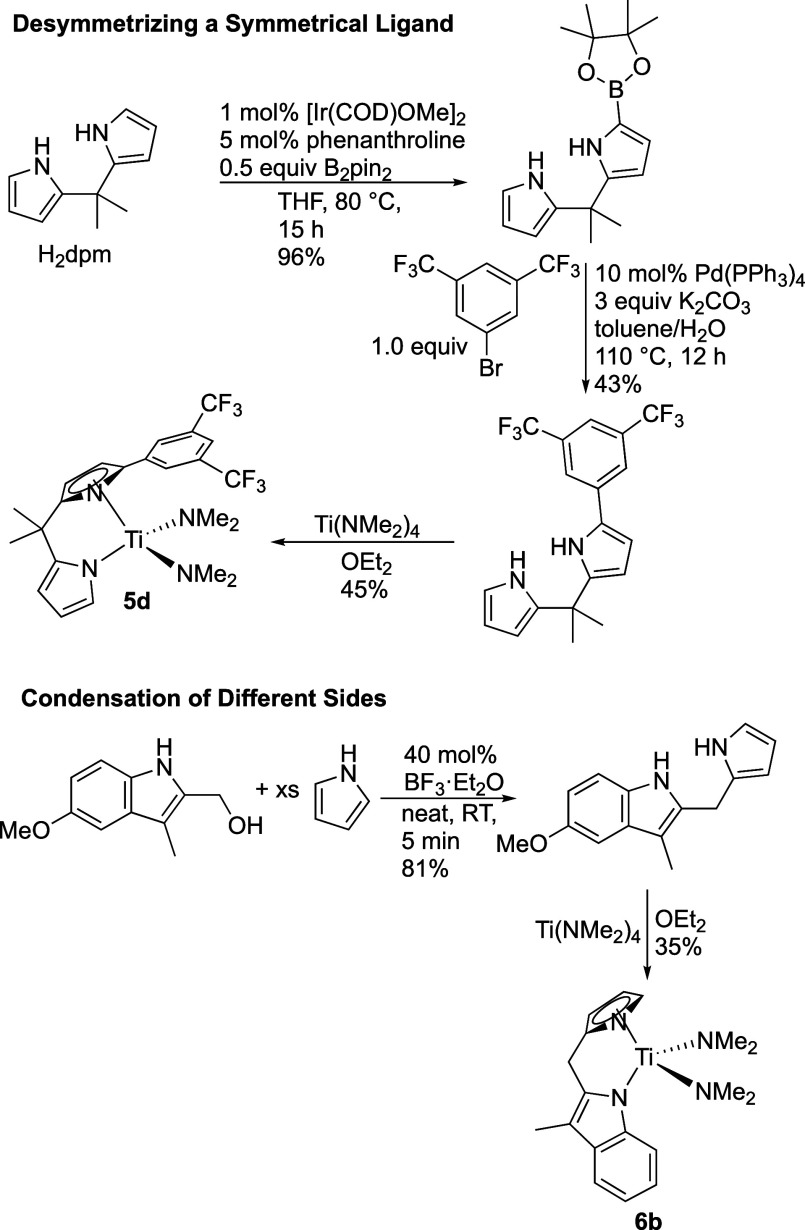
Examples of Methods for the Synthesis of Unsymmetrical Bidentate Ligands and Titanium Catalysts The top example demonstrates desymmetrizing a symmetrical ligand, and the bottom example demonstrates condensation to form the unsymmetrical ligand.

**Figure 1:**
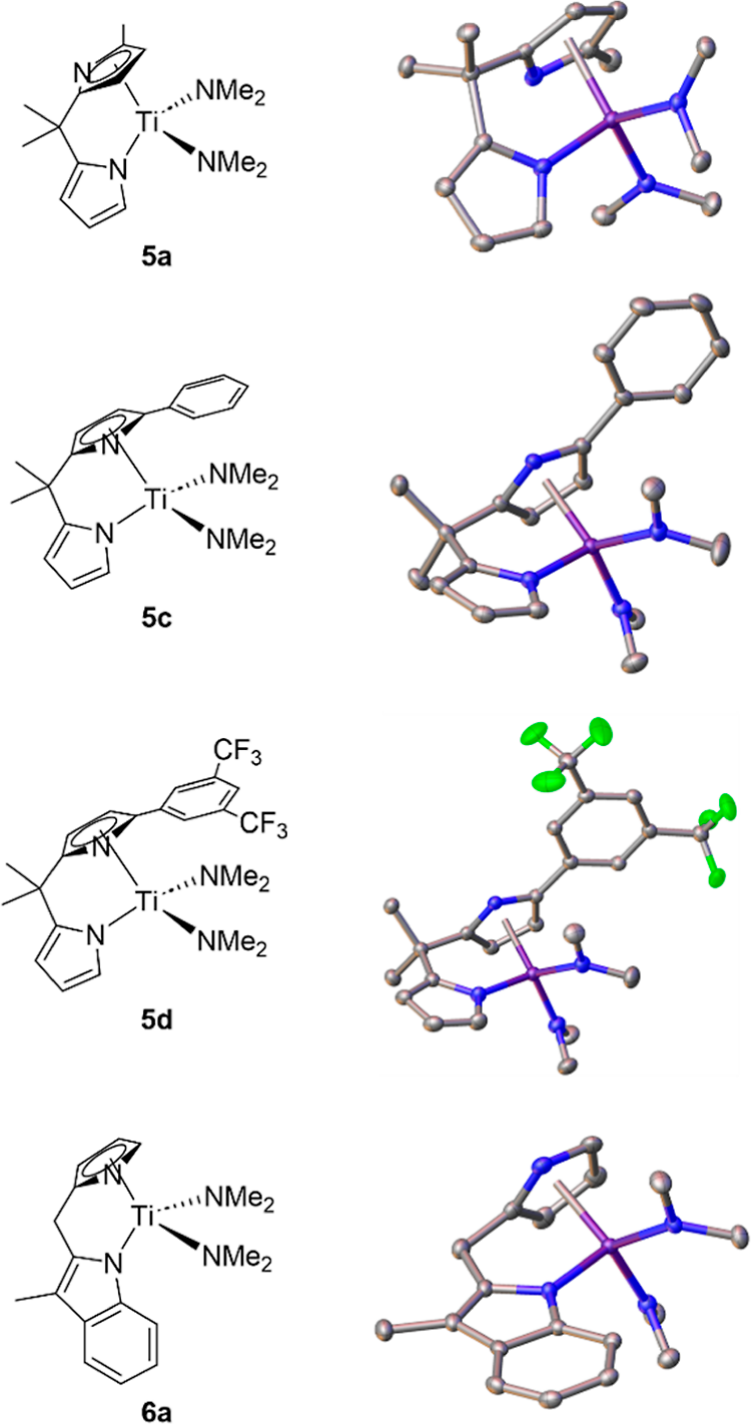
Structures of unsymmetrical catalysts characterized by single-crystal X-ray diffraction. The line drawing to the left is in approximately the same orientation as the ORTEP drawing on the right.

One can imagine two different strategies for the synthesis of unsymmetrical bidentate ligands (Scheme 2): (1) taking a symmetrical bidentate ligand and desymmetrizing it or (2) condensing two different ligand types into a bidentate framework. Both strategies were employed to generate the unsymmetrical ligands in this study (Figure 1), and the details are provided in the Supporting Information.

Scheme 2 shows examples of the desymmetrizing and condensation strategies carried through to precatalyst formation. To prepare a dipyrrolylmethane ligand with an aromatic group on one of the α-positions of one pyrrole, conditions were found to allow addition of a single pinacolboryl group to H_2_dpm, H_2_dpm = 5,5-dimethyldipyrrolylmethane (see Scheme 2) using iridium catalysis.^30,31^ Interestingly, very little of the diborylated product is observed, and the reaction appears very selective for the borylation of one side of the ligand. Then, Suzuki–Miyaura coupling was used to install the aromatic substituent.^32^

Unsymmetrical ligands also were generated using a strategy where one side of the nascent bidentate ligand was fitted with an alcohol (−CH_2_OH or −CMe_2_OH)^33^ and then coupled with a heterocycle under acid catalysis (BF_3_·OEt_2_^34^ or InCl_3_).

**Chart 3:**
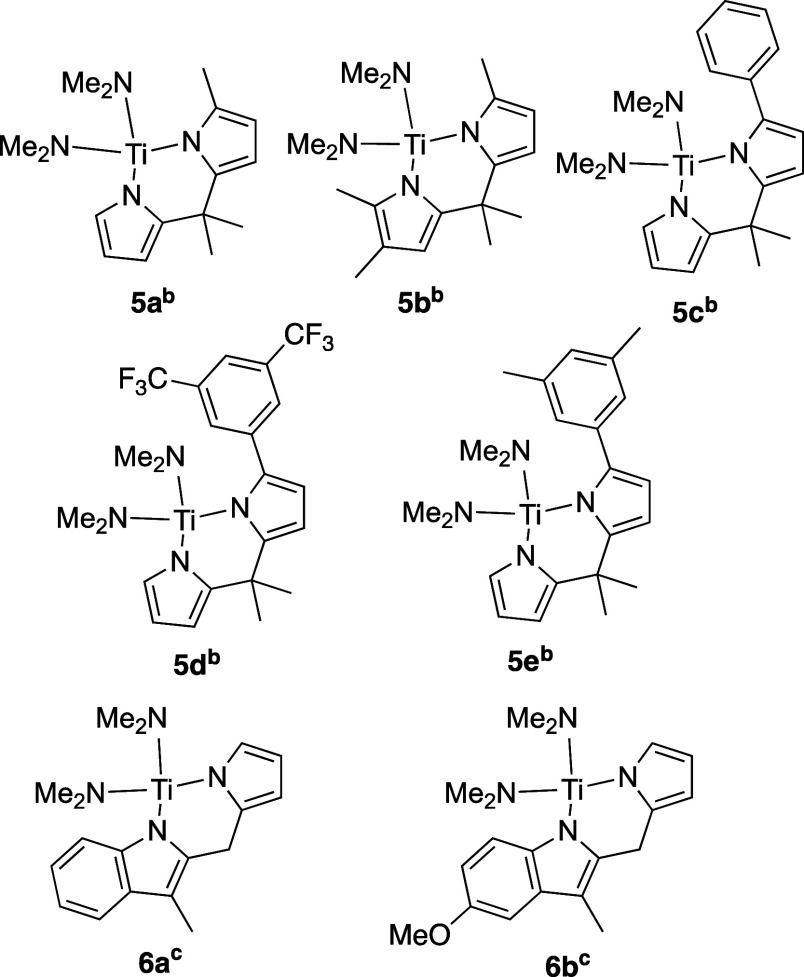
Unsymmetrical Precatalysts Investigated in This Studya

Once the unsymmetrical ligands were in hand, the reaction with Ti(NMe_2_)_4_ provided the precatalysts in high yield. For use in the titanium-catalyzed reaction, the precatalysts can be prepared and used in situ, but for this study, all the precatalysts were isolated and characterized (see the Supporting Information for details). In Chart 3 are all the precatalysts with unsymmetrical ligands investigated in this study, divided into dipyrrolylmethane derivatives (**5**) and 3-methylindolyls (**6**) containing ligands.

a All ligands shown as η^1^ for clarity but may not be in the solid-state structures, vide infra. b Bidentate ligands prepared by desymmetrization of a more symmetric ligand. c Bidentate ligands prepared by condensation of different fragments (see Scheme 2).

In the end, 7 new unsymmetrical precatalysts were synthesized with various components. The linkers between the two sides were either CH_2_ or CMe_2_ depending on the synthetic expediency. In our previous study, precatalysts with different linkers were explored (Chart 1), and the rates were determined by the electronics of the attaching components, not the linkers or the ring size of the metallacycle.^28^

For the unsymmetrical dipyrrolylmethane-derived ligands, one of the pyrroles was often η^5^ in the solid state. For example, **5d** (Chart 3) with an electron-deficient aromatic in the α-position has a substituted η^5^-pyrrolyl in the solid state by X-ray diffraction.

We structurally characterized 4 of the 7 new unsymmetrical catalysts by single-crystal X-ray diffraction—**5a**, **5c**, **5d**, and **6a**. The structures are shown in Figure 1. In the bis(pyrrolyl) containing **5** structures, the more sterically encumbered ligand (larger % *V*_bur_) is η^5^ in the solid state; this is regardless of whether the substituted pyrrolyl is more or less donating electronically (based on LDP). For the pyrrolyl/indolyl **6a**, unsurprisingly, the more aromatic pyrrolyl ligand is bound in an η^5^-fashion to the metal center.

For all the unsymmetrical catalysts (Chart 3), the two different η^5^,η^1^-configurations were calculated (B3PW91/def2TZVP), and the more stable configuration has the substituted pyrrolyl η^5^, and the indolyls are always energetically preferred to be η^1^ (see the Supporting Information for details).

**Figure 2:**
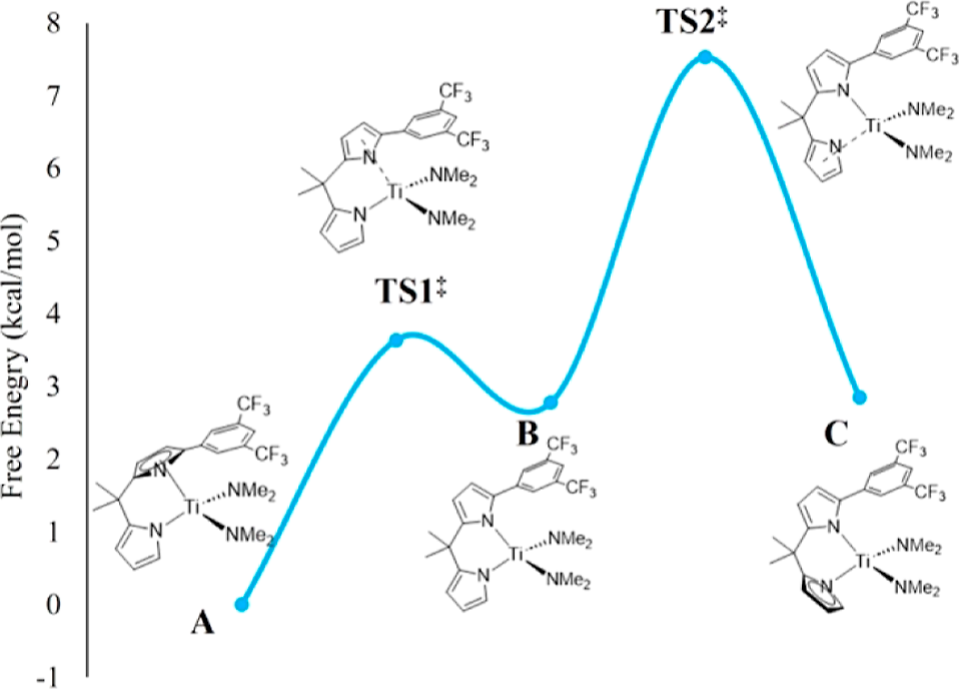
Free energy diagram for the computed structures of catalyst **5d**. The structure observed from single-crystal X-ray diffraction (**A**) was found to be about 3 kcal/mol below the η^1^,η^1^-structure and the structure with unsubstituted ring η^5^.

The fastest catalyst described here, **5d**, was studied both computationally and experimentally for its isomerization. Shown in Figure 2 are the relative energies of the computed (B3PW91/def2TZVP) structures. The gas phase calculation reproduces the structure from X-ray diffraction relatively well. The Ti–N(η^1^-pyrrolyl) distance computationally and experimentally are 2.001 and 2.020(2) Å, respectively. (There are two molecules of **5d** in the asymmetric unit, and the averages are being given for the two molecules.) The two dimethylamides in the structure are quite different, with one below the aromatic ring appended to the pyrrolyl ring and one away (Figure 1). The dimethylamide under the aromatic ring is forced to have its N–C–C plane parallel to the plane of the aromatic. The Ti–N(amide^away^) distances computationally and experimentally were found to be 1.892 and 1.895(2) Å, respectively. The Ti–N(amide^under^) distances computationally and experimentally were found to be 1.888 and 1.900(2) Å, respectively. The Ti–N(η^5^-pyrrolyl) distance computationally and experimentally are 2.254 and 2.273(1) Å, respectively.

The energy difference (B3PW91/def2TZVP) between the observed solid-state configuration and the structure where the less substituted ring is the η^5^-pyrrolyl is 3 kcal/mol. In Figure 2, the lowest energy structure with the substituted pyrrolyl η^5^ (Figure 2, **A**) was placed at 0 kcal/mol in relative free energy. The calculation suggests a small barrier (**TS1**^**⧧**^) for conversion of **A** to **B**, the structure with both pyrrolyl groups η^1^. Structure **B** has about the same energy as that of **C**, the structure with unsubstituted pyrrolyl η^5^. The barrier from **B** to **C** (**TS2**^**⧧**^) was found to be approximately 8 kcal/mol.

Variable-temperature NMR on **5d** in the aliphatic region clearly shows two inequivalent methyl groups in the backbone of the bidentate ligand, inequivalent due to methyls being syn to the nitrogen of the η^5^-pyrrolyl and anti.^29^ Considering the calculations show a small barrier (**TS1**^**⧧**^) for exchange of **A** to **B**, it is assumed that this occurs faster than the NMR time scale. By line shape analysis, we measured a barrier for isomerization of Δ*G*^⧧^ = 9.1 ± 0.6 kcal/mol for what we presume to be **C** to the fast-exchanging **A** and **B**. This is quite consistent with the DFT value of 8 kcal/mol (Figure 2). More details can be found in the Supporting Information.

### Collection of Rate Data and Modeling

**Figure 3:**
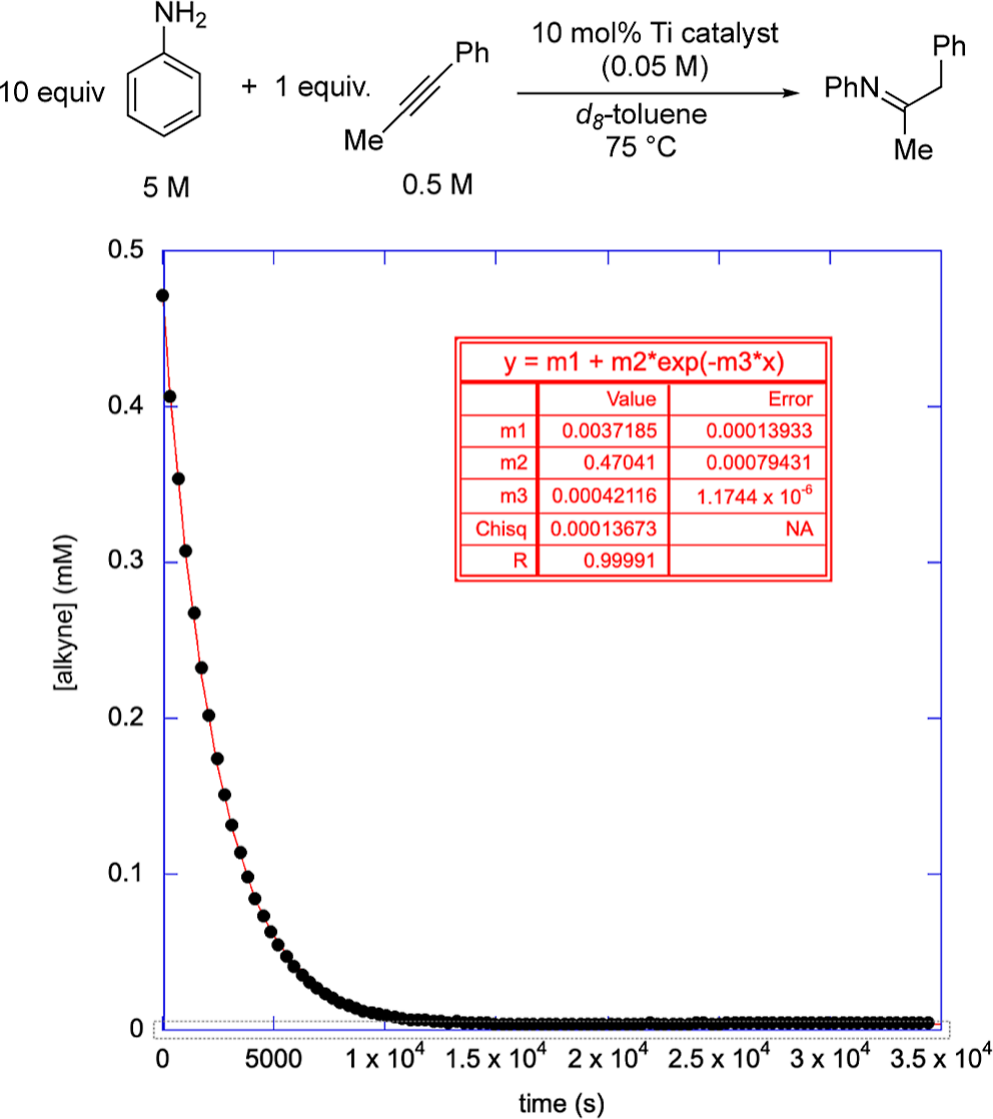
Conditions for the measurement of kinetics by ^1^H NMR and a representative plot (for **5e**) with fit. The disappearance of the alkyne starting material was used to measure the rate of reaction.

These unsymmetrical precatalysts were used in hydroamination catalysis under pseudo-first-order conditions identical to those used previously for the symmetrical precatalysts.^28^ The reaction conditions and a representative plot are provided in Figure 3. The reaction used was hydroamination of 1-phenylpropyne by excess aniline, which gives data that fit well to first-order plots. The fit of the kinetic data was done using Espenson’s first-order equation for an instrument response.^35^ Additional details regarding the collection of the kinetic data and additional fitted plots of the data are available in Supporting Information. Depending on the precatalyst, the half-life for the catalytic reaction varied from days to less than 30 min under the reaction conditions.

In our previous study,^28^ somewhat disappointingly, the fastest precatalyst examined, without unwanted side reactions, was the most common catalyst employed for the reaction at the time, **1a**. By making the ancillary ligand unsymmetrical, we hoped to discover catalysts faster than **1a**, and *in this case, we did find catalysts that were faster by the optimization of each side independently* (vide infra).

### Modeling of the Reaction Rate for Ti Hydroamination

To fit the data, decisions had to be made as to how the diverse set of ligands could be mathematically represented. We employed a 5-parameter model for the ancillary ligand structures using one LDP and one steric descriptor (% *V*_bur_) for each side of the ligand (eq 1). The LDP and % *V*_bur_ values were taken from our previous study on the symmetrical ligands.^28^ The subscripts _1_ and _2_ refer to different sides of the bidentate ligand (vide infra). Then, *a*–*e* are parameters found through regression.
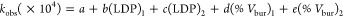
1

One assumption of statistical analysis in most cases is that the experimental error is normally distributed; however, in this case, a plot of the residuals shows that the error increases as the rate constant increases. In other words, accurate measurement of the rate under our standard conditions becomes more difficult with larger errors as the rate becomes faster. Because the error is not normally distributed, the regression statistics can be off ideal. A procedure for fixing this issue was developed, which involves scaling the data to get normally distributed error, Box–Cox analysis,^36,37^ which gave a modest improvement (*R*^2^ = 0.98) to the model over using the natural rate constants (*R*^2^ = 0.97) with improved standard errors on the parameters. The details of the Box–Cox analysis and the regression can be found in Supporting Information. The weighted values were calculated using eq 2a, with λ = 0.7, and a description of how λ was obtained can be found in Supporting Information. In eq 2a, *y* = experimentally obtained response (rate constant), *Y*_*i*_ = the weighted response, and *ẏ* = the geometric mean of all the responses.
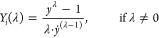
2a
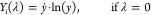
2b

While the model does not tell us the exact nature of the key transition state, it can give clues as to whether the structure has the two sides of the unsymmetrical ligand (e.g., the pyrrolyl and indolyl of the bidentate ligand in **6a**) in different or similar environments. As will be discussed, the fitting parameters are significantly different for the two sides of the bidentate ligand, which implies that the two sides of the ligand are in different environments, i.e., different types of sites on the metal such as axial and equatorial in trigonal bipyramidal, in the key transition state for the reaction.

**Scheme 3:**
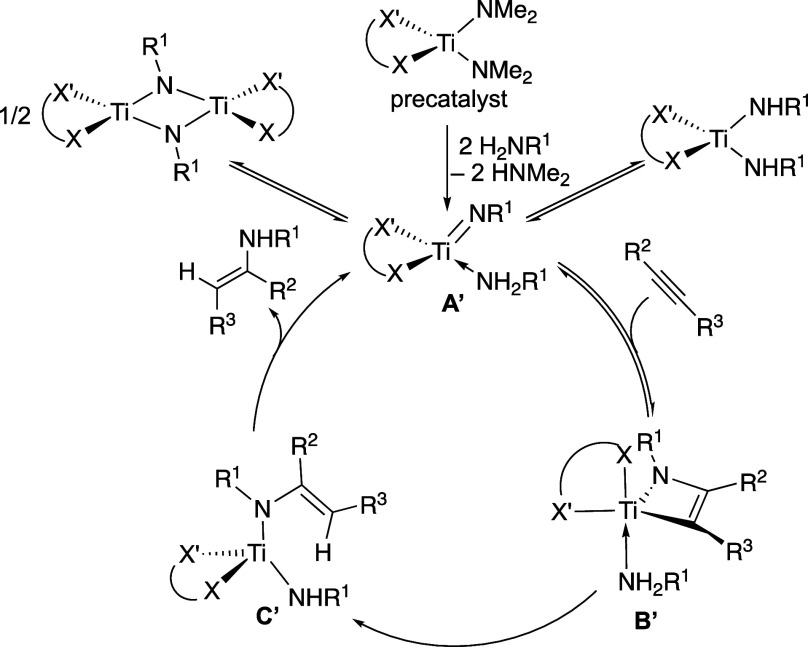
Possible Mechanism for Titanium Hydroamination Where **B′** Is Represented as a Key Intermediate and Ligand Tuning Is Perhaps Affecting Equilibria Associated with Its Formation and/or Protonolysis The mechanism shown assumes a trigonal bipyramidal intermediate. This is not the only possibility for the geometry based on the data.

To illustrate, we can make the supposition that the key transition state is the protonolysis of the Ti–C bond,^38,39^ as has been suggested for related systems. For the sake of discussion, we will assume that a trigonal bipyramidal structure such as **B′** is involved. One could then draw a mechanism, as shown in Scheme 3. The ancillary ligand’s electronics and sterics are presumably affecting the rate of the protonolysis step or the equilibria involved to reach **B′**. The model does not give the exact structure of the complexes involved, e.g., **B′** or the transition state between **B′** and **C′**, but it does make suggestions about the structure. For example, the model parameters for the two sides of the bidentate ligand (X and X′) are quite different in the key transition state, suggesting that the sites they occupy around the metal are quite different. This would not be the case for pseudotetrahedral complexes like **A′** or **C′** and is more consistent with a trigonal bipyramidal or square pyramidal structure, where X and X′ are in axial and equatorial positions, respectively, as shown in Scheme 3 for **B′**.

In addition, the model can suggest whether the sterics or the electronics of the two sides of the ligand determine how the ligand resides in the key step of the catalysis. In other words, if we assign one side (e.g., X in **B′**, Scheme 3) of the ligand as “1” and the other side (X′) as “2”, the modeling will suggest if the larger % *V*_bur_ or LDP value (our steric and electronic descriptors, respectively) determines whether X or X′ is axial or equatorial. It was found that assigning side 1 to the larger LDP value, i.e., making the assignment based on a donor ability, gave a significantly better model (see the Supporting Information for more details). Using the donor ability, i.e., LDP, to assign the ligand numbers gives *R*^2^ = 0.98 for the Box–Cox weighted rate constants (*R*^2^ = 0.97 for the unweighted values). If one assumes that the sterics determine the position of the ligand by setting side 1 based on the % *V*_bur_ values, the regression shows poorer statistics with *R*^2^ = 0.94 (or 0.93 using the unweighted values, see the Supporting Information). In short, the modeling suggests that the electronics of X′ and X in **B′** (Scheme 3) determine which ligand is in which position, e.g., equatorial vs axial, and not the sterics. It is worth noting here that this is *experimental* evidence regarding the nature of the key step for catalysis under the assumptions of the study.

In Table 1 are the model parameters from the regression analysis using the Box–Cox weighted natural parameters with all of the precatalysts with symmetrical ligands (Chart 1) and ligands **5a–e** and **6a/b** (Chart 3), for a total of 17 precatalysts. If one wants to calculate the rate of a catalyst not explored in this study, substitution of the LDP and % *V*_bur_ values for the ligand in question with the *a*–*e* values in the Table into eq 1 will give the weighted rate constant, provided that the mechanism is the same as all of the other ligands in the training set. Also, in Table 1 are the scaled parameters (−1 to +1), which give parameters that are directly comparable if one wants to see how the stereoelectronic properties of each side of the ligand affect the reaction rate. The 95% confidence intervals are shown (see the Supporting Information for more details on the error analysis).

**Table 1:** Parameters for Eq 1 for Titanium Hydroamination and Statistics for the Regression

parameter^a^ (descriptor)	natural^b^ parameters	scaled^b^ parameters (−1 to +1)
*a* (intercept)	–8.8 ± 2.5	–0.43 ± 0.18
*b* (LDP)_1_	2.4 ± 0.34	3.0 ± 0.42
*c* (LDP)_2_	–0.72 ± 0.42	–0.90 ± 0.53
*d* (% *V*_bur_)_1_	–0.17 ± 0.071	–0.63 ± 0.26
*e* (% *V*_bur_)_2_	–0.41 ± 0.068	–1.5 ± 0.25

a The descriptors are given in parentheses simply to show which parameter goes with which descriptor. In the model, the parameter given for the natural value is multiplied by the descriptor value (e.g., LDP) for that specific ligand side.

b The regression was done on the Box–Cox weighted rate constants (eq 2a). See the Supporting Information for the data analysis. The regression using the unweighted data gives similar values, which are given in the Supporting Information. For the fit, *R*^2^ = 0.98, adjusted *R*^2^ = 0.97, *n* = 17, and *F* = 140 (*p*-value = 6 × 10^–10^). The *p*-values for all the individual parameters are <0.005. The values shown (*u*_*i*_) in the table are *u*_*i*_ = *b*_*i*_ ± *t*_crit_·*s*_*i*_, where *b*_*i*_ = parameter from regression, *t*_crit_ = critical *t*-value for the 95% probability level and 12 degrees of freedom (2.179),^40^ and *s*_*i*_ = standard error. More details on the error analysis can be found in the Supporting Information.

**Figure 4:**
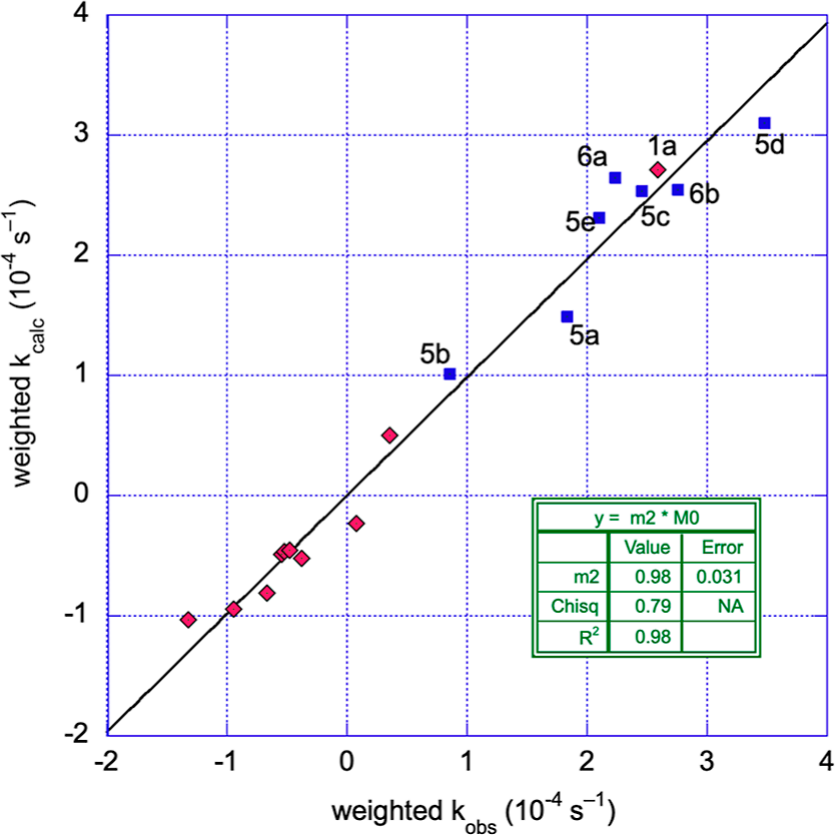
Plot of weighted (eq 2a) calculated rate constants using the model vs the weighted observed rate constants for all of the complexes used to calculate the model. Precatalysts with symmetrical ligands (Chart 1) are shown as red diamonds and precatalysts with unsymmetrical ligands **5a–e** and **6a/b** (Chart 3) are shown as blue squares and are labeled with the individual catalyst number. A total of 17 precatalysts were used in the model. The linear fit then (black line) should, ideally, have a slope of 1.00. See Supporting Information for more details regarding the modeling.

A plot of the calculated (using the model) weighted rate constants vs experimental weighted rate constants is shown in Figure 4 for all 17 of the precatalysts with symmetrical bidentate ligands (Chart 1) and unsymmetrical bidentate ligands (Chart 3). All the data used in the model fit are shown with the symmetrical catalysts as red diamonds and the unsymmetrical catalysts as blue squares. The linear fit, which ideally would have a slope of 1, has a slope of 0.98 ± 0.03 with *R*^2^ = 0.98. As can be seen, the unsymmetrical ligands, in general, were faster than their symmetrical counterparts, likely due to their more flexible electronic and steric structures; i.e., the unsymmetrical ligand can possibly place the different sides of the ligand in a more energetically favorable position, whereas a symmetrical ligand lacks this flexibility.

On the other hand, the sterics of side 2 (the side of the bidentate ligand with the lower LDP value) makes a larger contribution to the rate than the sterics of side 1 (side with the higher LDP value). Based on the signs and magnitudes of the parameters, to maximize the rate, side 1 should have a ligand that does not donate strongly and is relatively small, while the donor ability of side 2 is less important (a stronger donor appears to be somewhat better), but it is quite important that side 2 be small.

From the values for the parameters, the donor ability for side 1 (higher LDP side) makes a significant contribution to the rate of the reaction and increases the rate constant at larger values (poorer donor); however, the donor ability of side 2 seems to make a smaller contribution and perhaps increases the rate slightly at smaller LDP values (stronger donor). This results in complex **6b**, with a relatively electron-rich indolyl and electron-deficient pyrrolyl, being the second fastest catalyst examined, for example.

## Concluding Remarks

In previous work, we prepared 10 different precatalysts (Chart 1) using symmetrical, bidentate ancillary ligands (symmetrical in this context means that the ligand has X = X′, Scheme 3, on both sides of the bidentate ligand, i.e., the bidentate ligand has *C*_2*v*_ symmetry). In that case, a mathematical model of how the hydroamination rate constant varied with the structure of the ancillary ligand on the precatalyst suggested that the rate increased with electron-deficient and small ancillary ligands. Here, we examined the unsymmetrical ligands (Figure 1), where the two sides of the bidentate ligand are different sterically and electronically, and we found that this can lead to faster rates.

The model of the catalysts bearing symmetrical and unsymmetrical ligands in titanium hydroamination suggests that the position of the ligand in the key transition state is determined by the electronics (here the poorer donating end of the bidentate ligand is called “side 1”), and that this side of the ancillary ligand most importantly needs to have a poorly donating ligand (positive coefficient on descriptor LDP_1_) to get good reaction rates and is ideally small (negative coefficient on % *V*_bur_). The other side of the ancillary ligands (“side 2” with the more donating fragment) needs, most importantly, to be small to get good reaction rates, and possibly being electron-donating slightly increases the rate.

The model developed here clearly defines how to optimize a bidentate ligand on titanium for alkyne hydroamination, and precatalysts such as **5d** are faster than the previously discovered fastest symmetrical precatalyst, Ti(dpm)(NMe_2_)_2_ (**1a**). Unsymmetrical **5d** has a relatively large electron-deficient pyrrolyl ring, which becomes “side 1” in the model due to its larger LDP value (again, a large LDP value means the ligand is a poor donor). The scaled parameters can be directly compared to determine their importance to the rate constant. For side 1, the relatively large parameter *b* = +3.0 ± 0.42 means that as the LDP increases, the rate rapidly increases. The sterics for side 1 are of little importance, *d* = −0.63 ± 0.26, and the large size of side 1 in **5d** is only a minor impedance to the rate. For the more electron-donating side of the chelating ligand, side 2, the electronics make very little difference, but a more electron-donating ligand is somewhat preferred, *c* = −0.90 ± 0.53. However, it is more important that side 2 be small, *e* = −1.5 ± 0.25. As a result, the unsubstituted pyrrolyl, which acts as side 2 in **5d**, is well suited to its small size, even though it is more electron-deficient than some other ligand types.

In general, unsymmetrical catalysts were found to be faster than symmetrical systems for the set of substrates investigated. That the unsymmetrical catalysts were generally faster than the symmetrical catalysts could be broadly true for catalytic systems where the two sides of the bidentate ligand are in different environments, e.g., where one side of the ancillary ligand is axial and the other equatorial for a catalysis involving a trigonal bipyramidal key transitional state. If making the two sides of a bidentate ligand different does not change the rate, it may imply a more symmetrical key transition state, e.g., pseudotetrahedral, where the two sides of the ancillary ligand are in essentially the same environment or simply a poor response to ligand tuning.

In ongoing studies, we are examining the use of this method in understanding other catalytic reactions involving high oxidation state metals.

## Supporting Information Available

X-ray diffraction data on new precatalysts has been submitted to the Cambridge Crystallographic Database with reference numbers 2305757–2305760. Experimental details for the production of new precatalysts and characterization data; details on the kinetics and fits; additional details on the modeling and kinetics; and some additional information on DFT calculations (PDF)
Single-crystal X-ray diffraction data of 5a (CIF)
Single-crystal X-ray diffraction data of 5c (CIF)
Single-crystal X-ray diffraction data of 5d (CIF)
Single-crystal X-ray diffraction data of 8a (CIF)

## Supplementary Material

cs3c05658_si_001.pdf

cs3c05658_si_002.cif

cs3c05658_si_003.cif

cs3c05658_si_004.cif

cs3c05658_si_005.cif
