# Supplementary material for: Modeling Complex Ligands for High Oxidation State Catalysis: Titanium Hydroamination with Unsymmetrical Ligands
Source: ACS Catal. 2024 Mar 29;14(8):5531–8. doi: 10.1021/acscatal.3c05658 (PMC11036360; doi:10.1021/acscatal.3c05658)
Supplement: Supplementary file 1 — cs3c05658_si_001.pdf [file cs3c05658_si_001.pdf]

Supporting Information

Modeling Complex Ligands for High Oxidation State Catalysis: Titanium Hydroamination with Unsymmetrical Ligands

*Zhilin Hou‡, Rashmi Jena‡, Tanner J. McDaniel, Brennan S. Billow, Seokjoo Lee, Hannah I. Barr, and Aaron L. Odom**

Department of Chemistry, Michigan State University, 578 S. Shaw Ln, East Lansing, MI 48824

‡These authors contributed equally to this work: Zhilin Hou, Rashmi Jena

*Correspondence to: odoma@msu.edu

Table of Contents

General Considerations 6

Synthesis of Ligands 7

Synthesis of 2-(2-(1H-pyrrol-2-yl)propan-2-yl)-5-methyl-1H-pyrrole 7

Synthesis of $H_2dpm^{2,2'\text{-DiMe-3-CHO}}$ 7

Synthesis of $H_2dpm^{2,2',3\text{-TriMe}}$ 8

Synthesis of $H_2dpm^{2\text{-Bpin}}$ 9

General procedure A for H_2dpm derivatives 10

Synthesis of $H_2dpm^{2\text{-Ph}}$ 10

Synthesis of $H_2dpm^{2\text{-(3,5-diCF3Ph)}}$ 11

Synthesis of $H_2dpm^{2\text{-(3,5-diMe)}}$ 11

Synthesis of (1-tosyl-1H-pyrrol-2-yl)methanol....12

Synthesis of 2-((1-tosyl-pyrrol-2-yl)methyl)-3-methylindole....13

Synthesis of 2-((1H-pyrrol-2-yl)methyl)-3-methylindole....13

Synthesis of (5-methoxy-3-methyl-1H-indol-2-yl)methanol....14

Synthesis of 2-((1H-pyrrol-2-yl)methyl)-5-methoxy-3-methyl-1H-indole....14

Synthesis of Titanium Catalysts.... 15

Synthesis of Ti(dpm$^{2\text{-Me}}$)(NMe$_2$)$_2$ (**5a**)....15

Synthesis of Ti(dpm$^{3\text{-Me}}$)(NMe$_2$)$_2$ (**5b**)....15

Synthesis of Ti(dpm$^{2\text{-Ph}}$)(NMe$_2$)$_2$ (**5c**)....16

Synthesis of Ti(pyr$^{3,5\text{-CF3Ph}}$-C(CH$_3$)$_2$-pyr)(NMe$_2$)$_2$ (**5d**)....17

Synthesis of Ti(pyr$^{3,5\text{-diMePh}}$-C(CH$_3$)$_2$-pyr)(NMe$_2$)$_2$ (**5e**)....17

Synthesis of complex Ti(pyr-CH$_2$-ind$^{3\text{-Me}}$)(NMe$_2$)$_2$ (**6a**)....18

Synthesis of Ti(pyr-CH$_2$-ind-3-Me-5-OMe)(NMe$_2$)$_2$ (**6b**)....18

General Procedure for Kinetics.... 19

Representative Plots from Kinetics.... 21

Figure S1. Plot of [1-phenylpropyne] vs time with Ti(dpm$^{2\text{-Me}}$)(NMe$_2$)$_2$ (**5a**)....21

Figure S2. Plot of [1-phenylpropyne] vs time with Ti(dpm$^{3\text{-Me}}$)(NMe$_2$)$_2$ (**5b**)....21

Figure S3. Plot of [1-phenylpropyne] vs time with Ti(dpm$^{2\text{-Ph}}$)(NMe$_2$)$_2$ (**5c**)....22

Figure S4. Plot of [1-phenylpropyne] vs time with Ti(pyr$^{3,5\text{-CF3Ph}}$-C(CH$_3$)$_2$-pyr)(NMe$_2$)$_2$ (**5d**)....22

Figure S5. Plot of [1-phenylpropyne] vs time with Ti(pyr$^{3,5\text{-diMePh}}$-C(CH$_3$)$_2$-pyr)(NMe$_2$)$_2$ (**5e**)....23

Figure S6. Plot of [1-phenylpropyne] vs time with Ti(pyr-CH2-ind3-Me)(NMe2)2 (6a)....23

Figure S7. Plot of [1-phenylpropyne] vs time with Ti(pyr-CH$_2$-ind$^{3\text{-Me-5-OMe}}$)(NMe$_2$)$_2$ (**6b**)....24

Gas Chromatography.... 25

Figure S8: (Top)Possible products from 1-phenylpropyne and aniline hydroamination. (Bottom) Gas Chromatograph from the crude reaction mixture.....25

Ligand η^1-η^5 Isomerization.... 26

Figure S9: (Top)Possible structures for catalysts (Bottom) Calculated ground state energies for isomers **A**, **B**, and **C** for all catalysts.....26

Figure S10: Calculated energy profile for catalyst Ti(pyr$^{3,5\text{-}CF3Ph}$-C(CH$_3$)$_2$-pyr)(NMe$_2$)$_2$ **5d** showing interconversion between each isomer (A, B, and C) and associated transition states (TS1‡ and TS2‡)..27

Figure S11: Variable temerpature NMR study for catalyst Ti(pyr$^{3,5\text{-}CF3Ph}$-C(CH$_3$)$_2$-pyr)(NMe$_2$)$_2$ **5d** 28

Modeling of the Kinetic Data 30

Table S1. Data for the Modeling of Natural Variables. 30

Errors Analysis Using Unweighted Rate Regression as Example 32

Modeling with the Ligand Site Determined by Sterics 35

Modeling of the Scaled Values........ 36

Table S2. Data for the Modelling with Scaled Parameters. 37

Weighting of Reaction Rates: Box-Cox Analysis 38

NMR Spectra for Ligands and Titanium Catalysts........ 41

Figure S12. ^{1}H NMR of H$_2$dpm$^{2\text{-}Me}$ 41

Figure S13. ^{13}C NMR of H$_2$dpm$^{2\text{-}Me}$ 42

Figure S14. ^{13}C NMR of H$_2$dpm$^{2\text{-}Me}$ 43

Figure S15. ^{1}H-NMR (top) and 1D-NOESY (bottom) spectra of H$_2$dpm$^{2,2'\text{-}DiMe\text{-}3\text{-}DiCHO}$ 44

Figure S16. ^{1}H NMR of H$_2$dpm$^{2,2',3\text{-}TriMe}$ 45

Figure S17. ^{13}C NMR of H$_2$dpm$^{2,2',3\text{-}TriMe}$ 46

Figure S18. ^{1}H NMR of H$_2$dpm$^{2\text{-}Bpin}$ 47

Figure S19. ^{1}H NMR of H$_2$dpm$^{2\text{-}Ph}$........ 48

Figure S20. ^{13}C NMR of H$_2$dpm$^{2\text{-}Ph}$........ 49

Figure S21. ^{1}H NMR of H$_2$dpm$^{2\text{-}(3,5\text{-}diCF3Ph)}$........ 50

Figure S22. ^{13}C NMR of H$_2$dpm$^{2\text{-}(3,5\text{-}diCF3Ph)}$........ 51

Figure S23. ^{19}F NMR of H$_2$dpm$^{2\text{-}(3,5\text{-}diCF3Ph)}$ 52

Figure S24. ^{1}H NMR of H$_2$dpm$^{2\text{-}(3,5\text{-}diMe)}$ 53

Figure S25. ^{13}C NMR of H$_2$dpm$^{2\text{-}(3,5\text{-}diMe)}$ 54

Figure S26. ^{1}H NMR of (1-tosyl-1H-pyrrol-2-yl)methanol 55

Figure S27. ^{1}H NMR of 3-methyl-2-((1-tosyl-pyrrol-2-yl)methyl)-1H-indole. Residual solvents peaks (DCM and hexanes) from workup. 56

Figure S28. ^{13}C NMR of 3-methyl-2-((1-tosyl-pyrrol-2-yl)methyl)-1H-indole....................57

Figure S29. ^{1}H NMR of 2-((1H-pyrrol-2-yl)methyl)-3-methylindole. Residual solvent peaks were observed for EtOAc and H_2O. 58

Figure S30. ^{13}C NMR of 2-((1H-pyrrol-2-yl)methyl)-3-methylindole 59

Figure S31. ^{1}H NMR of (5-methoxy-3-methyl-1H-indol-2-yl)methanol....................60

Figure S32. ^{13}C NMR of (5-methoxy-3-methyl-1H-indol-2-yl)methanol....................61

Figure S33. ^{1}H NMR of 2-((1H-pyrrol-2-yl)methyl)-5-methoxy-3-methyl-1H-indole. Residual solvent peaks were observed for hexane and H_2O....................62

Figure S34. ^{13}C NMR of 2-((1H-pyrrol-2-yl)methyl)-5-methoxy-3-methyl-1H-indole 63

Figure S35. ^{1}H NMR of Ti(dpm$^{2\text{-}Me}$)(NMe_2)$_2$ (**5a**) 64

Figure S36. ^{13}C NMR of Ti(dpm$^{2\text{-}Me}$)(NMe_2)$_2$ (**5a**) 65

Figure S37. ^{1}H NMR of Ti(dpm$^{2,2',3\text{-}TriMe}$)(NMe_2)$_2$ (**5b**) 66

Figure S38. ^{13}C NMR of Ti(dpm$^{2,2',3\text{-}TriMe}$)(NMe_2)$_2$ (**5b**) 67

Figure S39. ^{1}H NMR of Ti(dpm$^{2\text{-}Ph}$)(NMe_2)$_2$ (**5c**) 68

Figure S40. ^{13}C NMR of Ti(dpm$^{2\text{-}Ph}$)(NMe_2)$_2$ (**5c**) 69

Figure S41. ^{1}H NMR of Ti(pyr$^{3,5\text{-}CF3Ph}$-C(CH_3)$_2$-pyr)(NMe_2)$_2$ (**5d**) 70

Figure S42. ^{13}C NMR of Ti(pyr$^{3,5\text{-}CF3Ph}$-C(CH_3)$_2$-pyr)(NMe_2)$_2$ (**5d**) 71

Figure S43. ^{19}F NMR of Ti(pyr$^{3,5\text{-}CF3Ph}$-C(CH_3)$_2$-pyr)(NMe_2)$_2$ (**5d**) 72

Figure S44. ^{1}H NMR of Ti(pyr$^{3,5\text{-}diMePh}$-C(CH_3)$_2$-pyr)(NMe_2)$_2$ (**5e**) 73

Figure S45. ^{13}C NMR of Ti(pyr$^{3,5\text{-}diMePh}$-C(CH_3)$_2$-pyr)(NMe_2)$_2$ (**5e**) 74

Figure S46. ^{1}H NMR of Ti(pyr-CH_2-ind$^{3\text{-}Me}$)(NMe_2)$_2$ (**6a**) 75

Figure S47. ^{13}C NMR of Ti(pyr-CH_2-ind$^{3\text{-}Me}$)(NMe_2)$_2$ (**6a**) 76

Figure S48. ^{1}H NMR of Ti(pyr-CH_2-ind$^{3\text{-}Me\text{-}5\text{-}OMe}$)($NMe_2$)$_2$ (**6b**) 77

Figure S49. ^{13}C NMR of Ti(pyr-CH_2-ind$^{3\text{-}Me\text{-}5\text{-}OMe}$)($NMe_2$)$_2$ (**6b**) 78

Single Crystal X-ray Diffraction.................... 79

Figure S50. Structure of Ti(dpm$^{2\text{-}Me}$)(NMe_2)$_2$ (**5a**) recrystallized from toluene/n-hexane................79

Table S3. Crystallographic data and structural refinement of Ti(dpm$^{2\text{-}Me}$)(NMe_2)$_2$ (**5a**)..................79

Figure S51. Structure of Ti(dpm$^{2\text{-}Ph}$)(NMe_2)$_2$ (**5c**) recrystallized from toluene/n-hexane.................80

Table S4. Crystallographic data and structural refinement of Ti(dpm$^{2\text{-}Ph}$)(NMe_2)$_2$ (**5c**)...................80

Figure S52. Structure of Ti(pyr$^{3,5\text{-}CF3Ph}$-C(CH_3)$_2$-pyr)(NMe_2)$_2$ (5d) recrystallized from ether/n-hexane. 82

Table S5. Crystallographic data and structural refinement of Ti(pyr$^{3,5\text{-}CF3Ph}$-C(CH_3)$_2$-pyr)(NMe_2)$_2$ (**5d**) 82

Figure S53. Structure of Ti(pyr-CH_2-ind$^{3\text{-}Me}$)(NMe_2)$_2$ (**6a**) recrystallized from ether/n-hexane........83

Table S6. Crystallographic data and structural refinement of Ti(pyr-CH_2-ind$^{3\text{-}Me}$)(NMe_2)$_2$ (**6a**)......83

References.. 85

General Considerations

All manipulations were carried out under an inert dinitrogen atmosphere in an MBraun glovebox or using standard Schlenk techniques. Toluene was sparged with purified dinitrogen and passed over an activated alumina column prior to use. *n*-Hexane was dried over sodium benzophenone radical, refluxed, and distilled under dinitrogen prior to use. All deuterated NMR solvents were purchased from Cambridge Isotope Laboratories. C_6D_6 was dried over CaH_2 and distilled under dinitrogen. $CDCl_3$ was dried over P_2O_5 and distilled under dinitrogen.

$Ti(NMe_2)_4$ was purchased from Gelest and used as received. Tetrakis(triphenylphosphine)palladium(0) was purchased from Strem and used as received. 1-phenyl-1-propyne was purchased from Combi-blocks and distilled from barium oxide prior to use. Aniline was purchased from Sigma-Aldrich and distilled from KOH and passed through dry alumina prior to use. $\{Ir(COD)OMe\}_2$, H_2dpm, and 5-formyl-2,2'-dimethylpyrroylmethane were prepared according to the literature procedures.[1-3]

Spectra were taken on Varian instruments located in the Max T. Rogers Instrumentation Facility at Michigan State University. These include an Agilent DDR2 500 spectrometer equipped with a 5 mm pulsed-field-gradient (PFG) OneProbe and operating at 499.955 MHz (^{1}H) and 125.77 MHz (^{13}C), a Varian Inova 600 spectrometer equipped with a 5 mm PFG switchable broadband probe operating at 599.89 MHz (^{1}H) and 564.30 MHz (^{19}F), a UNITY plus 500 spectrometer equipped with a 5 mm Pulsed-Field-Gradient (PFG) switchable broadband probe and operating at 499.955 MHz (^{1}H) and 125.77 (^{13}C). Single crystal data was collected on XtaLAB Synergy, Dualflex, Hypix diffractometer using CuK_α radiation. Data collection was done at 100 K under a continuous flow of liquid nitrogen. In Olex2 program, crystal structures were solved with ShelXT solution using intrinsic phasing and refined with the SheXTL refinement package using least squares minimization.[4[Sheldrick, 2015 #92] All hydrogens are refined anisotropically. All crystals were stable at room temperature for mounting.

Synthesis of Ligands

Synthesis of 2-(2-(1H-pyrrol-2-yl)propan-2-yl)-5-methyl-1H-pyrrole

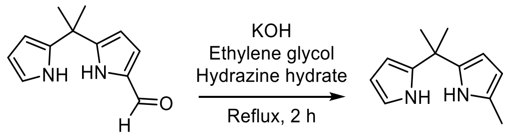

To a dry 100 mL Schlenk flask was added 5-formyl-2,2'-dimethylpyrroylmethane[2] (0.50 g, 2.47 mmol), KOH (0.99 g, 25.3 mmol), ethylene glycol (13 mL), and hydrazine hydrate (1.067 g, 21.31 mmol). The reaction mixture was degassed with nitrogen and stirred at 70 °C for 30 min. Then, the reaction was heated at reflux (200 °C) for 2 h. The reaction mixture was then allowed to cool to room temperature and diluted with water (50 mL) and DCM (50 mL). The aqueous layer was extracted with DCM (3 × 50 mL). The organic layers were combined, dried with Na_2SO_4, and filtered. The crude product was purified using column chromatography on silica gel, using hexanes/ethyl acetate as the eluent (9:1) to yield the product as a light brown solid (0.28 g, 60.2%). ^{1}H NMR (C_6D_6, 500 MHz): δ = 6.97 (s, 1H, pyrr-NH), 6.83 (s, 1H, pyrr-NH), 6.20-6.23 (m, 2H, pyrr-H), 6.10-6.11 (m, 1H, pyrr-H), 6.00-6.01 (app t, J = 2.8 Hz, 1H, pyrr-H), 5.88-5.89 (m, 1H, pyrr-H), 1.72 (s, 3H, pyrr-CH_3), 1.44 (s, 6H, $C(CH_3)_2$). $^{13}C\{^1H\}$ NMR (C_6D_6, 125 MHz): δ = 138.81, 137.10, 126.21, 116.66, 107.71, 105.52, 104.23, 103.65, 35.13, 29.37, 12.51. HRMS $[M+H]^+$: Found: *m/z* 189.1401; Calcd for $C_{12}H_{17}N_2$ 189.1392. MS (EI): m/z 188 (M^+). M.pt.: 97-98 °C.

Synthesis of $H_2dpm^{2,2'\text{-DiMe-3-CHO}}$

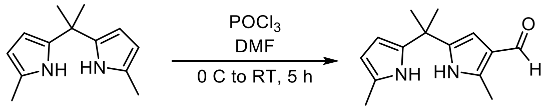

In a dry 50 mL Schlenk flask, under nitrogen, the Vilsmeier reagent was prepared by addition of $POCl_3$ (0.97 mL, 10.4 mmol) to excess DMF (9.0 mL, 117 mmol) dropwise at 0 °C. The Vilsmeier reagent was allowed to warm up to room temperature and stir for 15 min. While under nitrogen, this reagent was then added dropwise to a solution of DMF (5 mL) and $H_2dpm^{2,2'\text{-DiMe}}$ (2.31 g, 11.42 mmol) over 20 min at 0 °C. The reaction mixture was allowed to stir for 3 h at 0 °C. To this reaction mixture was added aqueous KOH solution (2 M, 60 mL) and DCM (100 mL). This solution was allowed to stir for 20 min. The organic layer

was than separated, washed with saturated aqueous NH_4Cl, washed with water, and then dried over Na_2SO_4. The solvents (DCM and DMF) were removed in vacuo to give the crude product as a viscous orange oil. The crude product was purified by column chromatography on silica gel using hexanes/ethyl acetate (9:1) as the eluent to yield the product as a light orange-red solid (1.34 g, 51% yield). There are two possible isomers of $H_2dpm^{2,2'\text{-DiMe-3-CHO}}$, the difference is between the two locations of the aldehyde relative to the dimethylmethane linker (shown in Figure S11). Therefore, if we saturated the signal for the methyl groups on the linker, labeled **E**, we can deduce which isomer is correct. For the structure on the right, if **E** is saturated, signals for the protons **A/I** (N–H), **F** (–CH), and **B** (–CHO) should be observed. For the structure on the left, if **E** is saturated, signals for the protons **C/F** (–CH) and **A/I** (N–H) should be observed. As shown at the bottom of Figure S11, when **E** is saturated the signals for **C/F** (–CH) and **A/I** (N–H) are observed, and **B** (–CHO) is absent. This suggests that the left structure is the correct isomer. ^{1}H NMR (DMSO-d^6, 500 MHz): δ = 11.00 (s, 1H, pyrr-NH), 10.06 (s, 1H, pyrr-NH), 9.63 (s, 1H, pyrr-CHO), 5.95-5.96 (d, *J* = 2.6 Hz, 1H, pyrr-H), 5.53-5.54 (t, *J* = 2.9 Hz, 1H, pyrr-H), 5.50 (m, 1 H, pyrr-H) 2.37 (s, 3H, pyrr-CH_3), 2.08 (s, 3H, pyrr-CH3), 1.49 (s, 6H, $C(CH_3)_2$). ^{13}C{^{1}H} NMR (DMSO-d^6, 125 MHz): δ = 184.60, 140.92, 138.76, 137.18, 126.37, 121.10, 104.63, 103.60, 102.58, 35.07, 28.70, 13.30, 11.33. HRMS $[M+H]^+$: Found: *m/z* 231.1498; Calcd for $C_{14}H_{19}N_2$ 231.1497. MS (EI): m/z 230 (M^+). M.pt.: 174-175 °C.

Synthesis of $H_2dpm^{2,2',3\text{-TriMe}}$

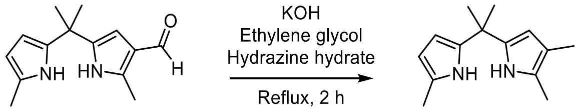

To a dry 100 mL Schlenk flask was added 5,5'-dimethyl-4-formyl-2,2'-dimethylpyrroylmethane (1.49 g, 6.49 mmol), KOH (3.71 g, 66.3 mmol), ethylene glycol (13 mL) and hydrazine hydrate (2.75 mL, 55.9 mmol). The reaction mixture was degassed with nitrogen and stirred at 70 °C for 30 min. Then, the reaction was heated at reflux (200 °C) for 2 h. The reaction mixture was then allowed to cool to room temperature and diluted with water (50 mL) and DCM (50 mL). The aqueous layer was extracted with DCM (3 × 50 mL). The organic layers were combined, dried with Na_2SO_4, and filtered. The crude product was purified

using column chromatography on silica gel using hexanes/ethyl acetate as the eluent (9:1) to yield the product as a light brown solid. (0.87 g, 62% yield). ^{1}H NMR (C_6D_6, 500 MHz): δ = 7.02 (s, 1H, pyrr-NH), 6.82 (s, 1H, pyrr-NH), 6.06-6.07 (appt, J = 2.9 Hz, 1H, pyrr-H), 5.90-5.92 (m, 2H, pyrr- H), 2.06 (s, 3H, pyrr-CH_3), 1.73 (s, 3H, pyrr-CH_3) 1.67 (s, 3H, pyrr-CH_3), 1.51 (s, 6H, $C(CH_3)_2$). ^{13}C NMR (C_6D_6, 125 MHz): δ = 137.63, 136.09, 126.03, 121.95, 113.01, 105.90, 105.64, 103.91, 35.08, 29.47, 12.50, 10.96, 10.33. HRMS $[M+H]^+$: Found: *m/z* 215.1548; Calcd for $C_{14}H_{19}N_2$ 215.1548. MS (EI): m/z 214 (M^+). M.pt.: 76-77 °C.

Synthesis of $H_2dpm^{2\text{-}Bpin}$

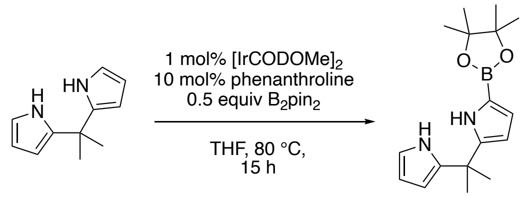

In a N_2 glovebox, {Ir(COD)OMe}$_2$ (7 mg, 0.01 equiv) and phenanthroline (2 mg, 0.1 equiv) were dissolved in THF (2 mL) and stirred for 5 min in a 25 mL Schlenk flask, then B_2pin_2 (127 mg, 0.5 equiv) was added. Lastly, H_2dpm (174 mg, 1.0 equiv) was added together with another portion of THF (3 mL). The reaction was removed from the glovebox and heated in an oil both at 80 °C for 15 h. The mixture was allowed to cool to room temperature, and THF was evaporated. The crude product was diluted with hexane (20 mL) and flushed through a silica plug. Removal of solvent afforded crude product as a light brown oil (290 mg, 96%). The product was used for the next step without further purification. ^{1}H NMR ($CDCl_3$, 500 MHz): δ 8.28 (s, 1H), 7.75 (s, 1H), 6.77 (t, J = 3.0 Hz, 1H), 6.66-6.61 (m, 1H), 6.18-6.14 (m, 1H), 6.14 (d, J = 2.7 Hz, 1H), 6.11-6.07 (m, 1H), 1.66(s, 6H), 1.29 (s, 12H). LRMS (EI): Calcd for $C_{17}H_{25}BN_2O_2$: 300, found: 300.

General procedure A for H_2dpm derivatives

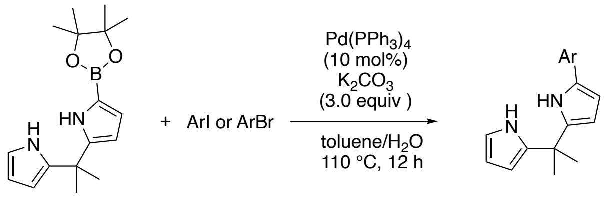

In a glove box, tetrakis(triphenylphosphine)palladium(0) (10 mol%), potassium carbonate (2.0 equiv), dpm-Bpin (1.0 equiv), aryl iodide or aryl bromide (1.0 equiv), and 3 mL (per mmol of dpm-Bpin starting material) of toluene were added to a 50 mL Schlenk tube. The reaction was then removed from the glovebox and charged with 1 mL of water under a constant flow of dry dinitrogen. Then, the Schlenk tube was sealed and heated in an oil bath at 110 °C for 12 h. The organic layer was diluted with EtOAc, separated, and dried over sodium sulfate. The crude product was purified by column chromatography on silica gel (hexane/ethyl acetate) to afford pure product.

Synthesis of $H_2dpm^{2\text{-}Ph}$

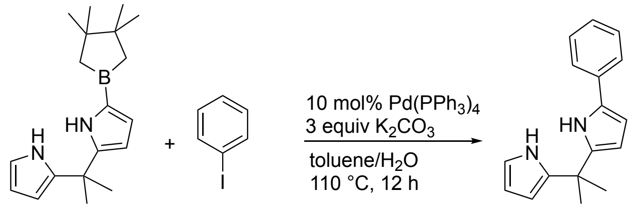

General procedure A was followed using $H_2dpm^{2\text{-}Bpin}$ (780 mg, 1.0 equiv), iodobenzene (583 mg, 1.1 equiv), tetrakis(triphenylphosphine)palladium(0) (300 mg, 10 mol%), potassium carbonate (1.07 g, 3.0 equiv), 5 mL of toluene, and 1 mL of water. Removal of solvent and purification by silica gel column chromatography (1:10 EtOAC:Hexanes) afforded product as a colorless oil (130 mg, 20%). ^{1}H NMR ($CDCl_3$, 500 MHz): δ 7.96 (s, 1H), 7.86 (s, 1H), 7.36 (dd, *J* = 18.6, 7.5 Hz, 4H), 7.18 (t, *J* = 7.3 Hz, 1H), 6.67 (s, 1H), 6.45 (s, 1H), 6.17 (d, *J* = 9.8 Hz, 3H), 1.71 (s, 6H). ^{13}C NMR (126 MHz, $CDCl_3$) δ 140.41, 138.81, 132.78, 131.49, 128.92, 126.10, 123.67, 117.29, 108.09, 105.87, 105.75, 104.00, 77.41, 77.16, 76.91, 35.70, 29.46. LRMS (EI): Calcd for $C_{17}H_{18}N_2$: 250; found: 250.

Synthesis of $H_2dpm^{2\text{-}(3,5\text{-}diCF3Ph)}$

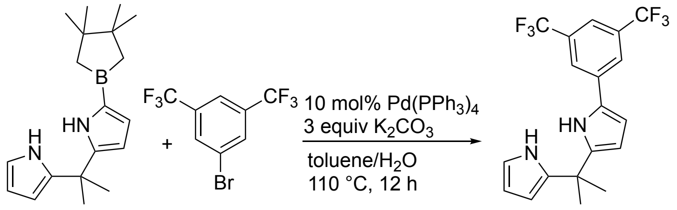

General procedure A was followed using $H_2dpm^{2\text{-}Bpin}$ (300 mg, 1.0 equiv), 1-bromo-3,5-bis(trifluoromethyl)benzene (293 mg, 1.0 equiv), tetrakis(triphenylphosphine)palladium(0) (164 mg, 4 mol%), potassium carbonate (392 mg, 2.0 equiv), toluene (3 mL), and water (1 mL). Removal of solvent afforded product as a colorless oil (165 mg, 43%). ^{1}H NMR ($CDCl_3$, 500 MHz): δ 8.03 (s, 1H), 7.86 (s, 1H), 7.72 (s, 2H), 7.62 (s, 1H), 6.71 (t, J = 3.4 Hz, 1H), 6.65 – 6.46 (m, 1H), 6.26 – 6.21 (m, 1H), 6.20 (s, 1H), 6.17 (d, J = 4.4 Hz, 1H), 1.73 (s, 6H). $^{13}C\{^{1}H\}$ NMR ($CDCl_3$, 126 MHz): δ $^{13}C\{^{1}H\}$ NMR ($CDCl_3$, 126 MHz): δ 142.75, 138.25, 134.69, 132.28 (q, 33.2 Hz, CF_3), 124.55, 123.20, 122.38, 119.01 (q, 4.1 Hz), 117.60, 108.67, 108.31, 106.87, 104.35, 77.42, 77.16, 76.91, 35.81, 29.37. ^{19}F NMR ($CDCl_3$, 470 MHz) δ –63.05. MS (EI): Calcd for $C_{19}H_{16}F_6N_2$: 386; found: 386.

Synthesis of $H_2dpm^{2\text{-}(3,5\text{-}diMe)}$

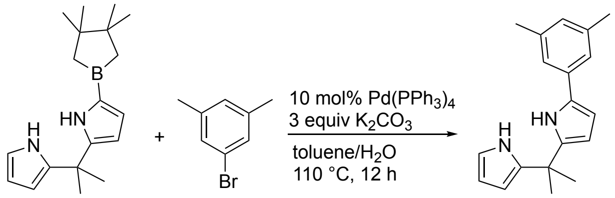

General procedure A was followed using $H_2dpm^{2\text{-}Bpin}$ (600 mg, 1.0 equiv), 1-bromo-3,5-dimethylbenzene (370 mg, 1.0 equiv), tetrakis(triphenylphosphine)palladium(0) (230 mg, 10 mol%), potassium carbonate (828 mg, 3.0 equiv), toluene (6 mL), and water (1 mL). The compound was purified by column silica gel chromatography using 3% EtOAc in hexanes. Removal of solvent afforded product as a colorless oil (400 mg, 72%). ^{1}H NMR ($CDCl_3$, 500 MHz): δ 7.93 (s, 1H), 7.85 (s, 1H), 6.98 (s, 2H), 6.83 – 6.78 (m, 1H), 6.66 (td, J = 2.6, 1.5 Hz, 1H), 6.38 (dd, J = 3.5, 2.7 Hz, 1H), 6.18 – 6.15 (m, 1H), 6.15 – 6.12 (m, 2H), 2.30 (s, 6H), 1.69 (s, 6H). $^{13}C\{^{1}H\}$ NMR ($CDCl_3$, 126 MHz): δ 163.14, 161.72, 141.57,

137.63, 133.67, 129.98, 124.22, 123.62, 115.14, 112.43, 107.88, 101.79, 47.11, 40.12, 30.10, 21.30. MS (EI): Calcd for $C_{19}H_{22}N_2$: 278; found: 278.

Synthesis of (1-tosyl-1H-pyrrol-2-yl)methanol

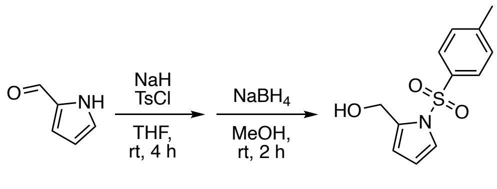

The procedure was adapted from the literature.[5] A 250 mL Schlenk flask equipped with a stir bar was purged with dinitrogen and charged with pyrrole-2-carboxaldehyde (2.0 g, 1.0 equiv) and THF (50 mL). Sodium hydride (0.6 g, 1.1 equiv) was added in 3 portions over 30 min. The mixture was stirred at room temperature until gas evolution ceased. Next, a solution of *para*-toluenesulphonyl chloride (4.4 g, 1.1 equiv) in THF (15 mL) was added to the reaction, and the mixture was stirred at room temperature for 4 h. The reaction was quenched with water (50 mL). The crude product was extracted with ethyl acetate and washed with brine. Removal of the solvent afforded crude product as a brown solid (5.4 g). ^{1}H NMR and ^{13}C NMR spectra were consistent with those previously reported.[6] ^{1}H NMR ($CDCl_3$, 500 MHz): δ 9.97 (s, 1H), 7.80 (d, J = 8.4 Hz, 2H), 7.62 (dd, J = 3.1, 1.8 Hz, 1H), 7.33 (s, 2H), 7.15 (dd, J = 3.8, 1.8 Hz, 1H), 6.40 (t, J = 3.4 Hz, 1H), 2.42 (s, 3H). The brown solid was used without further purification and dissolved in 30 mL of MeOH. Sodium borohydride (1.6 g, 2.0 equiv) was added in 3 portions over 10 min, and the resulting mixture was stirred for 2 h. To the reaction was added water (20 mL) then ethyl acetate (20 mL). The organic layer was separated, dried over Na_2SO_4, and evaporated to afford a brown solid. The crude product was purified by recrystallization in DCM/hexanes (white solid, 5.14 g, 97%). ^{1}H NMR and ^{13}C NMR spectra were consistent with those previously reported.[6] ^{1}H NMR ($CDCl_3$, 500 MHz): δ 7.71 (d, J = 8.6 Hz, 2H), 7.31 (d, J = 8.7 Hz, 2H), 7.28-7.25 (m, 1H), 6.25 (dd, J = 3.4, 1.9 Hz, 1H), 6.23 (t, J = 3.3 Hz, 1H), 4.60 (d, J = 5.7 Hz, 2H), 2.41 (s, 3H).

Synthesis of 2-((1-tosyl-pyrrol-2-yl)methyl)-3-methylindole

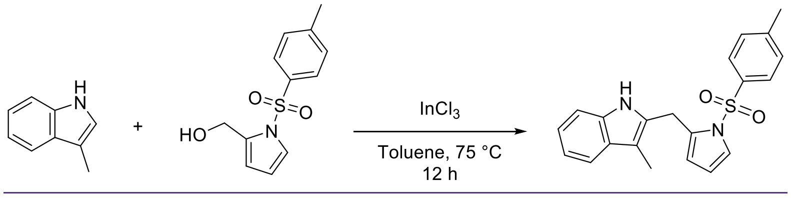

A 100 mL Schlenk flask was loaded with (1-tosyl-pyrrol-2-yl)methanol (502 mg, 1.0 equiv, 2 mmol), 3-methylindole (262 mg, 1.0 equiv, 2.0 equiv), $InCl_3$ (88.4 mg, 40 mol%), toluene (10 mL), and a stir bar. The reaction was heated in an oil bath at 75 °C for 12 h. After the removal of solvent, the product was purified by chromatography on silica gel (5% ethyl acetate in hexanes) to obtain the product as light brown solid (290 mg, 40%). ^{1}H NMR ($CDCl_3$, 500 MHz, 25 °C): δ 7.70 (s, 1H), 7.47-7.44 (m, 1H), 7.40 (d, J = 8.6 Hz, 2H), 7.33 (dd, J = 3.4, 1.8 Hz, 1H), 7.11-7.04 (m, 3H), 6.97 (d, J = 7.9 Hz, 2H), 6.23 (t, J = 3.4 Hz, 1H), 6.05 (s, 1H), 4.21(s, 2H), 2.24 (s, 3H), 2.21 (s, 3H). ^{13}C{^{1}H} NMR ($CDCl_3$, 126 MHz, 25 °C): δ 144.90, 135.85, 135.22, 131.84, 130.71, 129.76, 129.26, 126.45, 123.31, 121.43, 119.10, 118.39, 114.68, 111.63, 110.48, 107.95, 24.50, 21.61, 8.52. LRMS (EI): calc'd: 364; found: 364.

Synthesis of 2-((1H-pyrrol-2-yl)methyl)-3-methylindole

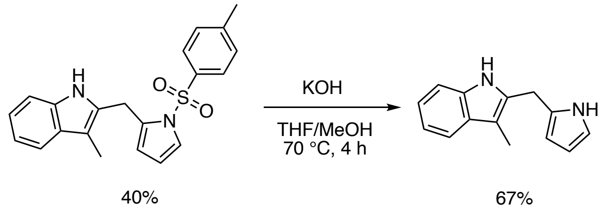

In 100 mL round bottom flask, KOH (178 mg, 4.0 equiv) was dissolved in a mixture of methanol (20 mL) and THF (20 mL). Then, the reaction was charged with 3-methyl-2-((1-tosyl-pyrrol-2-yl)methyl)indole (290 mg, 1.0 equiv) and heated at 70 °C for 4 h. After cooling to room temperature, ethyl acetate (20 mL) was added. The organic layer was washed with brine, separated, dried over Na_2SO_4, and evaporated to afford product as a grey solid (mg, 67%). ^{1}H NMR ($CDCl_3$, 500 MHz): δ 7.85 (s, 1H), 7.69 (s, 1H), 7.52 (d, J = 7.5 Hz, 1H), 7.22 (d, J = 7.0 Hz, 1H), 7.16-7.08 (m, 2H), 6.70-6.63 (m, 1H), 6.18 (q, J = 2.9 Hz, 1H), 6.09 (s, 1H), 4.12 (s, 2H), 2.31 (s, 3H). ^{13}C{^{1}H} NMR ($CDCl_3$, 126 MHz): δ 135.52, 131.58, 129.34, 128.36, 121.70, 119.34, 118.47, 117.56, 110.58, 108.75, 108.02, 106.83, 24.90, 8.62.

Synthesis of (5-methoxy-3-methyl-1H-indol-2-yl)methanol

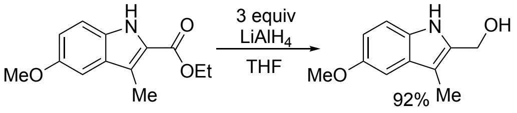

In a 250 mL 2-neck round-bottom flask, LAH (770 mg, 5.0 equiv) was added to dry THF (40 mL) under a constant flow of N_2. A solution of ethyl 3-formyl-5-methoxy-1H-indole-2-carboxylate (1.0 g, 1.0 equiv) in dry THF (10 mL) was added dropwise. The reaction was stirred at 40 °C for 12 h. After the reaction cooled to room temperature, the reaction was slowly quenched with 1 M HCl and extracted with ethyl acetate. The organic layer was washed with brine, separated, and dried over sodium sulfate. The evaporation of the solvent afforded crude product as a light yellow solid (710 mg, 92%). ^{1}H NMR ($CDCl_3$, 500 MHz): δ 8.06 (s, 1H), 7.20 (d, J = 8.7 Hz, 1H), 6.97 (d, J = 2.6 Hz, 1H), 6.84 (dd, J = 8.7, 2.5 Hz, 1H), 4.79 (s, 2H), 3.87 (s, 3H), 2.25 (s, 3H). ^{13}C{^{1}H} NMR ($CDCl_3$, 126 MHz): δ 154.03, 133.90, 130.88, 129.33, 112.44, 111.65, 108.38, 100.90, 56.82, 56.06, 8.58.

Synthesis of 2-((1H-pyrrol-2-yl)methyl)-5-methoxy-3-methyl-1H-indole

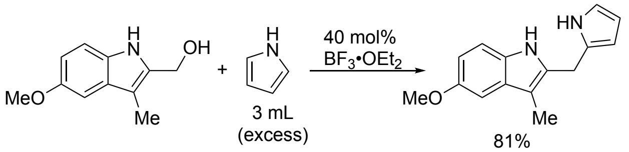

In a 25 mL Schlenk flask, (5-methoxy-3-methyl-1H-indol-2-yl)methanol (450 mg, 1.0 equiv) was dissolved in an excess of pyrrole (3 mL). Slowly, $BF_3{\cdot}Et_2O$ (0.12 mL, 0.4 equiv) was added dropwise to the solution under a constant flow of N_2. The reaction rapidly turned purple. The mixture was allowed to stir at room temperature for 5 min. The reaction was quenched by adding 20 mL of saturated sodium bicarbonate solution. The product was extracted by adding EtOAc (20 mL). The organic layer was separated and dried over sodium sulfate. Excess pyrrole was removed under vacuum. The crude product was purified by column chromatography (silica gel, 30% ethyl acetate in hexanes). Removal of solvent afforded the product as a colorless oil (460 mg, 81%). ^{1}H NMR (DMSO-d_6, 500 MHz): δ 10.52 (s, 1H), 10.40 (s, 1H), 7.13 (d, J = 8.6 Hz, 1H), 6.87 (d, J = 2.4 Hz, 1H), 6.63 (dd, J = 8.7, 2.4 Hz, 1H), 6.60-6.55 (m, 1H), 5.88 (q, J = 2.8 Hz, 1H), 5.73 (s, 1H), 3.94 (s, 2H), 3.74 (s, 3H), 2.15 (s, 3H). ^{13}C{^{1}H} NMR (DMSO-d_6, 126

MHz): δ 152.88, 134.22, 130.31, 129.05, 128.91, 116.42, 111.17, 109.93, 107.28, 105.31, 105.08, 99.88, 55.33, 24.44, 8.42. LRMS (EI): Calcd for $C_{15}H_{16}N_2O$: 240; found: 240.

Synthesis of Titanium Catalysts

*Synthesis of Ti(dpm$^{2\text{-}Me}$)(NMe$_2$)$_2$ (**5a**)*

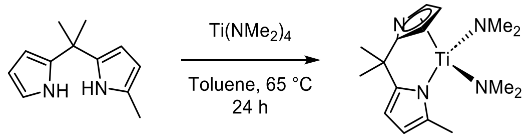

A 35 mL pressure tube equipped with a stir bar was loaded with $Ti(NMe_2)_4$ (0.575 g, 2.57 mmol, 1 equiv) and toluene (3 mL). A 20 mL scintillation vial was loaded with H_2dpm^{2Me} (0.483 g, 2.57 mmol, 1 equiv) and toluene (3 mL). Both solutions were cooled in a liquid nitrogen cold well for 15 min. The cold solution of H_2dpm^{2Me} was added dropwise to the vigorously stirring solution of $Ti(NMe_2)_4$. The reaction was allowed to warm and then stir at room temperature for 1 h. The pressure tube was then sealed and heated at 65 °C. The reaction progress was monitored by ^{1}H NMR and was completed after 24 h. Volatiles were removed in vacuo to give a light red solid. This solid was rinsed with cold pentane to yield the product as an orange powder (0.67 g, 82% yield). X-ray quality crystals can be grown by dissolving the complex in the minimum amount of pentane and cooling to –30 °C. ^{1}H NMR (C_6D_6, 500 MHz): δ = 7.05 – 6.97 (m, 1H), 6.59 – 6.45 (m, 1H), 6.35 (d, *J* = 2.4 Hz, 1H), 6.20 (d, *J* = 3.2 Hz, 1H), 6.01 (d, *J* = 2.6 Hz, 1H), 2.96 (s, 12H), 2.03 (s, 3H), 1.82 (s, 6H) $^{13}C\{^1H\}$ NMR (C_6D_6, 125 MHz): δ = 161.93, 161.70, 140.25, 123.61, 114.76, 113.67, 107.65, 101.56, 46.91, 39.54, 29.68, 15.01. Elemental Analysis: Calcd for $C_{17}H_{29}N_4Ti$. C, 59.63; H, 8.13; N, 17.38. Found: C, 59.55; H, 8.48; N, 17.89. M.pt.: 127-128 °C.

*Synthesis of Ti(dpm$^{3\text{-}Me}$)(NMe$_2$)$_2$ (**5b**)*

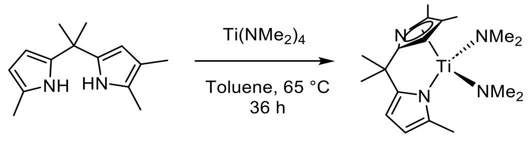

A 35 mL pressure tube equipped with a stir bar was loaded with $Ti(NMe_2)_4$ (0.228 g, 1.02 mmol, 1 equiv) and toluene (3 mL). A 20 mL scintillation vial was loaded with $H_2dpm^{2,2',3\text{-}TriMe}$ (0.220 g, 1.02 mmol,

1 equiv) and toluene (3 mL). Both solutions were then cooled in a liquid nitrogen cold well for 15 min. The cold solution of $H_2dpm^{2,2',3\text{-TriMe}}$ was added dropwise to the vigorously stirred solution of $Ti(NMe_2)_4$. The reaction was allowed to warm and stir at room temperature for 1 h. The pressure tube was then sealed and heated at 65 °C. The reaction progress was monitored by ^{1}H NMR and was complete after 36 h. Volatiles were removed *in vacuo* to give a light red solid. This solid was rinsed with cold pentane to yield the product as a red-orange powder (0.252 g, 71% yield). X-ray quality crystals can be grown by dissolving the complex in the minimum amount of pentane and cooling to –30 °C. ^{1}H NMR (C_6D_6, 500 MHz): δ = 6.13 (d, *J* = 2.8 Hz, 1H, pyrr-H) 5.99-6.00 (m, 2H, pyrr-H), 2.85 (s, 12H, $N(CH_3)_2$), 2.29 (s, 3H, CH_3), 2.14 (s, 3H, CH_3), 1.83 (s, 6H, $C(CH_3)_2$), 1.80 (s, 3H, CH_3). $^{13}C\{^1H\}$ NMR (C_6D_6, 126 MHz): δ = 162.12, 160.84, 135.35, 134.62, 121.78, 112.46, 108.29, 105.75, 46.76, 39.61, 30.35, 16.25, 13.68, 11.64. M.pt.: 108-109 °C.

*Synthesis of Ti(dpm$^{2\text{-}Ph}$)(NMe_2)$_2$ (**5c**)*

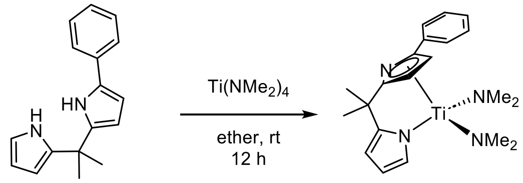

In 20 mL scintillation vial with a stir bar was loaded with $Ti(NMe_2)_4$ (0.068 g, 0.3 mmol, 1 equiv) and diethyl ether (3 mL). A 20 mL scintillation vial was loaded with H_2dpm^{2Ph} (0.074 g, 0.3 mmol, 1 equiv) and diethyl ether (3 mL). Both solutions were then cooled in a liquid nitrogen cold well for 15 min. The cold solution of H_2dpm^{2Ph} was added dropwise to the vigorously stirred solution of $Ti(NMe_2)_4$. The reaction mixture was stirred at room temperature for 12 h. The volatiles were removed in vacuo to give a sticky red-orange solid. This solid was washed with cold pentane three times, which afforded analytically pure orange solid (50 mg, 44%). X-ray quality crystals were grown from a saturated solution in pentane. ^{1}H NMR (C_6D_6, 500 MHz): δ = ^{1}H NMR (500 MHz, C_6D_6) δ 7.71 (d, *J* = 8.1 Hz, 2H), 7.14 – 7.07 (m, 2H), 7.03 (d, *J* = 8.9 Hz, 2H), 6.60 (d, *J* = 2.6 Hz, 1H), 6.53 (d, *J* = 2.7 Hz, 1H), 6.48 (d, *J* = 2.7 Hz, 1H), 6.29 – 6.16 (m, 1H), 2.77 (s, 12H), 1.87 (s, 6H). ^{13}C NMR (C_6D_6, 126 MHz,) δ 163.24, 161.74, 140.91, 133.81, 128.35, 128.31, 125.57, 124.14, 115.03, 112.68, 107.91, 101.78, 47.09, 40.08, 30.04. M.pt: 154-156 °C.

*Synthesis of Ti(pyr$^{3,5\text{-}CF3Ph}$-C(CH$_3$)$_2$-pyr)(NMe$_2$)$_2$ (**5d**)*

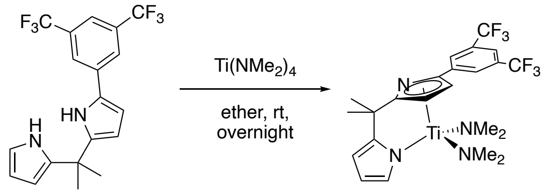

In 20 mL scintillation vial with a stir bar was loaded with Ti(NMe$_2$)$_4$ (0.058 g, 0.26 mmol, 1 equiv) and diethyl ether (3 mL). A 20 mL scintillation vial was loaded with pyr$^{3,5\text{-}CF3Ph}$-C(CH$_3$)$_2$-pyr (0.100 g, 0.26 mmol, 1 equiv) and diethyl ether (3 mL). Both solutions were then cooled in a liquid nitrogen cold well for 15 min. The cold solution of pyr$^{3,5\text{-}CF3Ph}$-C(CH$_3$)$_2$-pyr was added dropwise to the vigorously stirred solution of Ti(NMe$_2$)$_4$. The reaction mixture was stirred at room temperature for 12 h. The volatiles were removed in vacuo to give a viscous red-orange solid. Recrystallization from ether/n-hexane afforded product as orange crystals (60 mg, 45%). ^{1}H NMR (C$_6$D$_6$, 500 MHz): δ 8.09 (s, 2H), 7.64 (s, 1H), 6.88 (dd, J = 2.3, 1.3 Hz, 1H), 6.37 (s, 2H), 6.31 (dd, J = 3.1, 2.3 Hz, 1H), 6.17 (dd, J = 3.0, 1.3 Hz, 1H), 2.58 (s, 12H), 1.78 (s, 6H). ^{13}C{^{1}H} NMR (C$_6$D$_6$, 126 MHz): δ 164.43, 161.08, 137.53, 136.91, 131.78, 131.51, 125.70, 125.25, 120.28, 113.33, 112.74, 109.61, 104.61, 46.81, 39.97, 29.89. ^{19}F NMR (C$_6$D$_6$, 470 MHz, 25 °C) δ –62.58. M.pt.: 143-144 °C.

*Synthesis of Ti(pyr$^{3,5\text{-}diMePh}$-C(CH$_3$)$_2$-pyr)(NMe$_2$)$_2$ (**5e**)*

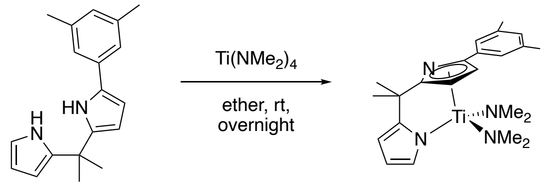

In 20 mL scintillation vial with a micro stir bar was loaded with Ti(NMe$_2$)$_4$ (0.292 g, 1.30 mmol, 1 equiv) and diethyl ether (3 mL). A 20 mL scintillation vial was loaded with pyr$^{3,5\text{-}diMePh}$-C(CH$_3$)$_2$-pyr (0.362 g, 1.30 mmol, 1 equiv) and diethyl ether (3 mL). Both solutions were then cooled in a liquid nitrogen cold well for 15 min. The cold solution of pyr$^{3,5\text{-}diMePh}$-C(CH$_3$)$_2$-pyr was added dropwise to the vigorously stirred solution of Ti(NMe$_2$)$_4$. The reaction mixture was stirred at room temperature for 12 h. The volatiles were removed in vacuo to give a sticky red-orange solid. Recrystallization from ether/n-hexane afforded the

product as orange crystals (300 mg, 56%). ^{1}H NMR (C_6D_6, 500 MHz): δ 7.51 (s, 2H), 7.06 (dd, J = 2.6, 1.3 Hz, 1H), 6.71 (s, 1H), 6.67 (d, J = 2.7 Hz, 1H), 6.58-6.55 (m, 1H), 6.51 (d, J = 2.7 Hz, 1H), 6.25 (dd, J = 3.1, 1.2 Hz, 1H), 2.80 (s, 12H), 2.13 (s, 6H), 1.89 (s, 6H). $^{13}C\{^{1}H\}$ NMR (C_6D_6, 126 MHz, 25 °C): δ 163.14, 161.72, 141.57, 137.63, 133.67, 129.98, 124.22, 123.62, 115.14, 112.43, 107.88, 101.79, 47.11, 40.12, 30.10, 21.30. M.pt.: 157-159 °C. Elemental Analysis: Calcd for $C_{24}H_{35}N_4Ti$. C: 66.99; H: 7.82; N: 13.59. Found: C: 66.90; H: 7.92; N: 13.30

*Synthesis of complex Ti(pyr-CH$_2$-ind^{3-Me})(NMe$_2$)$_2$ **(6a)***

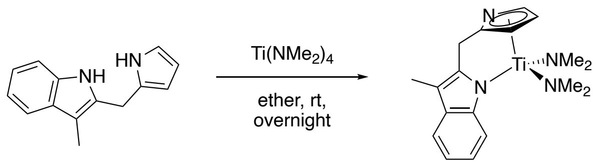

In 20 mL scintillation vial with a stir bar was loaded with $Ti(NMe_2)_4$ (0.320 g, 1.16 mmol, 1 equiv) and diethyl ether (3 mL). A 20 mL scintillation vial was loaded with Hpyr-CH$_2$-Hind^{3Me} (0.300 g, 1.16 mmol, 1equiv) and diethyl ether (3 mL). Both solutions were then cooled in a liquid nitrogen cold well for 15 min. The cold solution of Hpyr-CH$_2$-Hind^{3Me} was added dropwise to the vigorously stirred solution of $Ti(NMe_2)_4$. The reaction mixture was stirred at room temperature for 12 h. The volatiles were removed in vacuo to give a sticky red-orange solid. Recrystallization from ether/n-hexane afforded product as orange crystals (250 mg, 50%). ^{1}H NMR and $^{13}C\{^{1}H\}$ NMR show peaks for free dimethylamine. ^{1}H NMR (C_6D_6, 500 MHz): 77.67 (d, J = 7.7 Hz, 1H), 7.62 (d, J = 7.9 Hz, 1H), 7.40 (td, J = 7.5, 1.4 Hz, 1H), 7.35 (td, J = 7.3, 1.2 Hz, 1H), 6.95 (t, J = 1.4 Hz, 1H), 6.13 (dd, J = 2.7, 1.3 Hz, 1H), 5.91 (dd, J = 2.7, 1.5 Hz, 1H), 4.02 (s, 2H), 3.12 (s, 3H), 2.98 (s, 12H). $^{13}C\{^{1}H\}$ NMR (C_6D_6, 126 MHz, 25 °C): δ 155.79, 153.42, 143.92, 130.36, 129.33, 120.52, 119.60, 117.14, 116.76, 116.29, 115.31, 105.99, 47.39, 29.16, 9.24.

*Synthesis of Ti(pyr-CH$_2$-ind-3-Me-5-OMe)(NMe$_2$)$_2$ **(6b)***

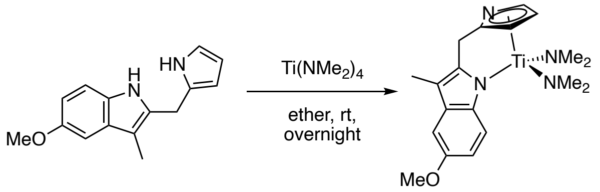

In 20 mL scintillation vial with a stir bar was loaded with $Ti(NMe_2)_4$ (0.390 g, 1.66 mmol, 1 equiv) and diethyl ether (3 mL). A 20 mL scintillation vial was loaded with Hpyr-CH_2-Hind$^{3\text{-Me-5-OMe}}$ (0.400 g, 1.66 mmol, 1 equiv) and diethyl ether (3 mL). Both solutions were then cooled in a liquid nitrogen cold well for 15 min. The cold solution of Hpyr-CH_2-Hind$^{3\text{-Me-5-OMe}}$ was added dropwise to the vigorously stirred solution of $Ti(NMe_2)_4$. The reaction mixture was stirred at room temperature for 12 h. The volatiles were removed in vacuo to give a sticky red-orange solid. Recrystallization from ether/n-hexane afforded product as orange crystals (217 mg, 35%). ^{1}H NMR (C_6D_6, 500 MHz) δ 7.50 (d, J = 8.2 Hz, 1H), 7.18 (d, J = 8.2 Hz, 2H), 6.96 (s, 1H), 6.13 (s, 1H), 5.91 (s, 1H), 4.02 (s, 2H), 3.67 (s, 3H), 2.99 (s, 13H), 2.22 (s, 3H). ^{13}C{^{1}H} NMR (C_6D_6, 126 MHz): δ 156.96, 155.14, 153.43, 139.26, 130.61, 128.42, 116.66, 116.19, 115.93, 109.94, 105.93, 99.56, 55.52, 47.42, 29.31, 9.36.

General Procedure for Kinetics

The procedure from the previous study was followed.[7] All manipulations were conducted in an N_2 glovebox. All measurements are done using volumetric syringes for accurate volumes. A 2 mL volumetric flask was loaded the titanium precatalyst (10 mol%, 0.1 mmol) and ferrocene (56 mg, 0.3 mmol). Toluene-d_8 (~0.75 mL) was added to the volumetric flask to completely dissolve the solids. Next, aniline (911 μL, 10 mmol) and 1-phenylpropyne (125 μL, 1.0 mmol) were added to the volumetric flask. Lastly, the solution was diluted up to 2 mL with toluene-d_8. The solution was mixed via a pipette, i.e., the solution was drawn up into the pipette and dispensed back into the volumetric flask, five times to ensure the homogeneity of the solution. Then, 0.75 mL solution was loaded into a threaded J. Young NMR tube that and removed from the dry box. This solution was heated at 75 °C in the NMR spectrometer (Varian Inova 600). The relative 1-phenylpropyne versus ferrocene concentration was monitored as a function of time. The fits are exponential decay of the starting material. The expression used to fit the data was $Y_t = Y_\infty + (Y_0 - Y_\infty)e^{-k_{obs}t}$,[8] where Y = concentration at time t (Y_t), infinity (Y_∞), or at the start of the reaction (Y_0). Each kinetic experiment was done in triplicates and the average value was used to represent rate constant for the catalyst under investigation.

Representative Plots from Kinetics

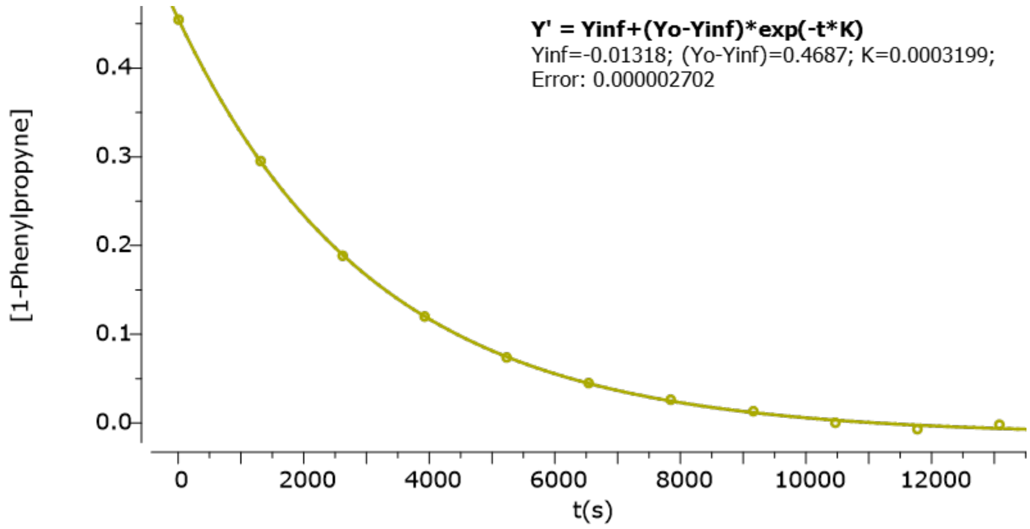

*Figure S1. Plot of [1-phenylpropyne] vs time with Ti(dpm$^{2\text{-}Me}$)(NMe$_2$)$_2$ (**5a**)*

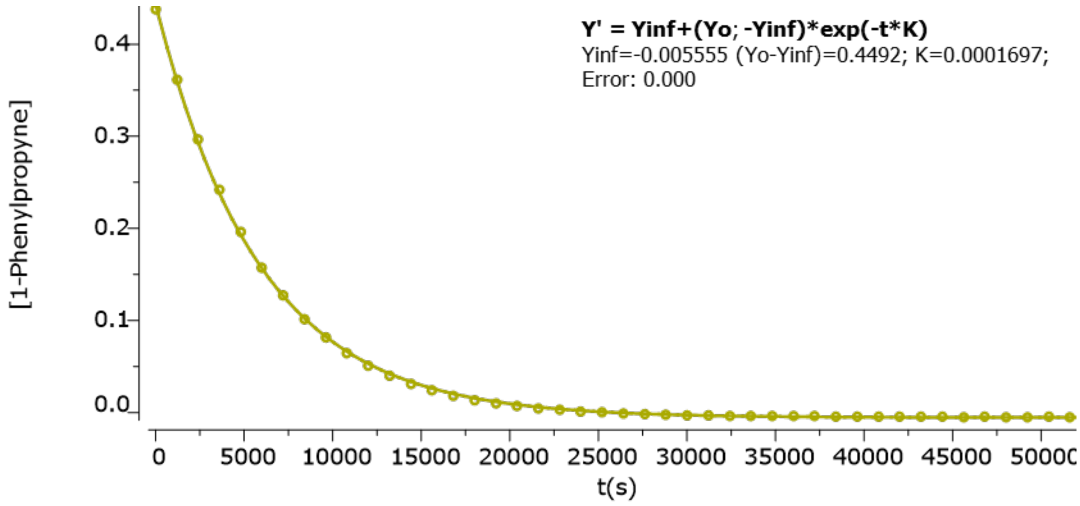

*Figure S2. Plot of [1-phenylpropyne] vs time with Ti(dpm$^{3\text{-}Me}$)(NMe$_2$)$_2$ (**5b**)*

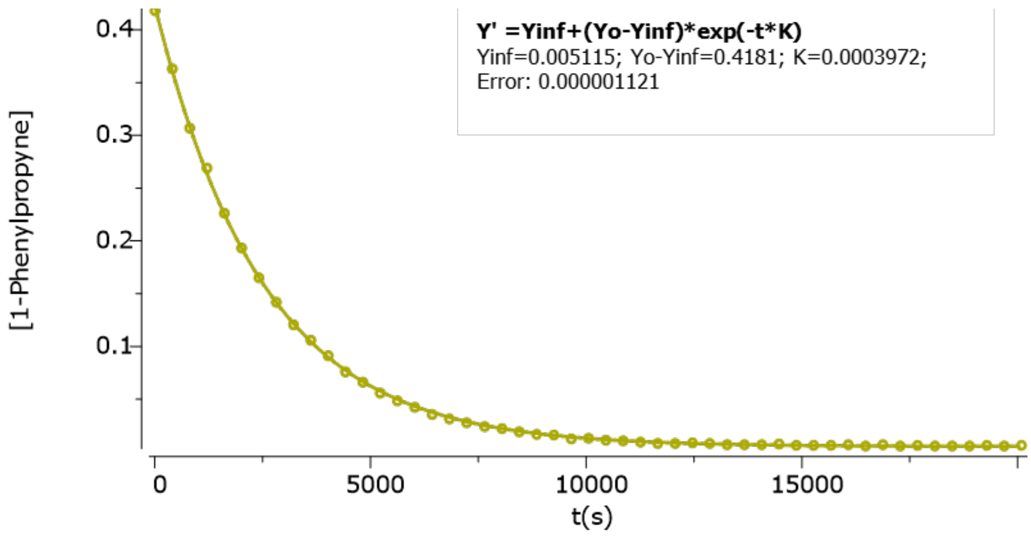

*Figure S3. Plot of [1-phenylpropyne] vs time with Ti(dpm$^{2\text{-}Ph}$)(NMe$_2$)$_2$ (**5c**)*

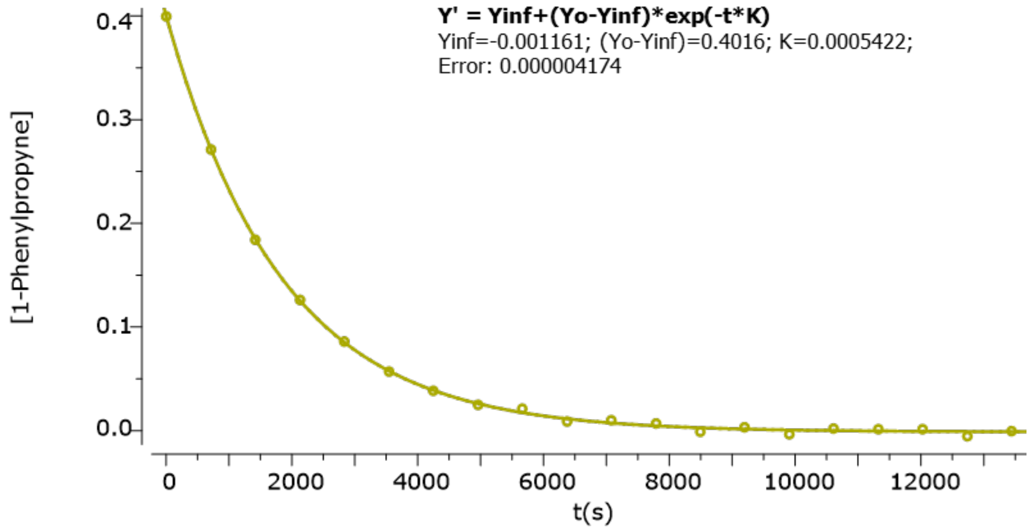

*Figure S4. Plot of [1-phenylpropyne] vs time with Ti(pyr$^{3,5\text{-}CF3Ph}$-C(CH$_3$)$_2$-pyr)(NMe$_2$)$_2$ (**5d**)*

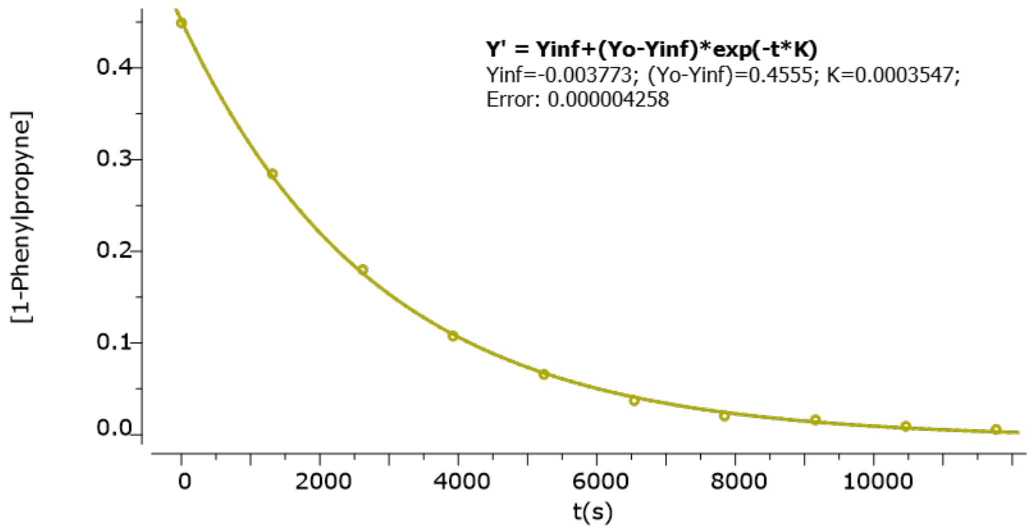

*Figure S5. Plot of [1-phenylpropyne] vs time with Ti(pyr$^{3,5\text{-}diMePh}$-C(CH_3)$_2$-pyr)(NMe_2)$_2$ (**5e**)*

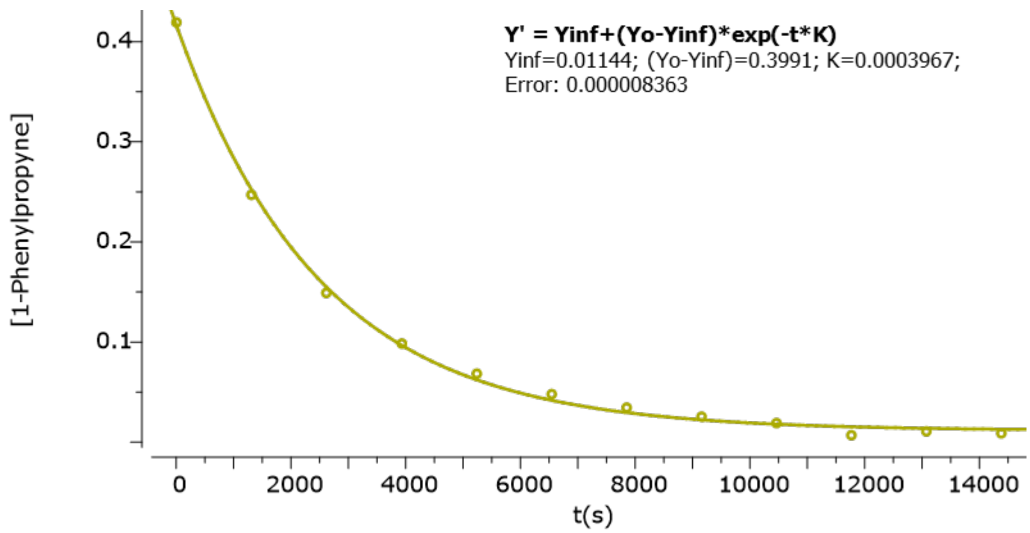

Figure S6. Plot of [1-phenylpropyne] vs time with Ti(pyr-CH2-ind3-Me)(NMe2)2 (6a)

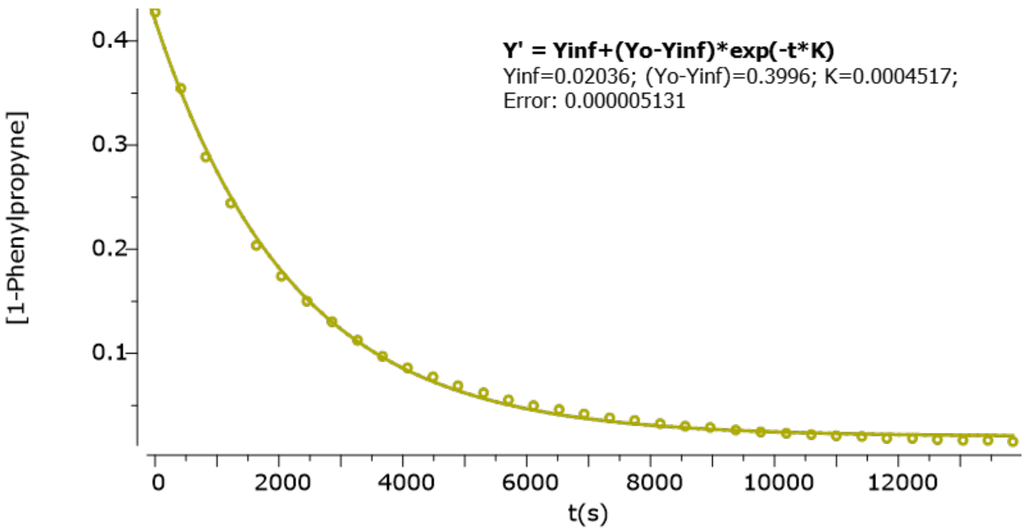

*Figure S7. Plot of [1-phenylpropyne] vs time with Ti(pyr-CH$_2$-ind$^{3\text{-}Me\text{-}5\text{-}OMe}$)(NMe$_2$)$_2$ (**6b**)*

Gas Chromatography

To show that there are no side reactions for the fastest catalyst (**5d**) examined here, we ran the reaction and examined products by GC. The GC sample was prepared by diluting the crude reaction mixture after passing through silica plug to remove metal. The hydroamination of 1-phenylpropyne and aniline could give 2 regioisomers as shown in Figure S8. The chromatogram here shows two peaks at retention time 9.43 mins and 9.37 mins, corresponding to two imine isomers **C** (major) and **D** (minor). Some of the imine product was hydrolyzed to ketone during silica plug filtration, leading to ketone **E** (retention time = 5.23 mins). Peaks at 4.05, 4.70, 10.98 mins corresponds to aniline (**A**), 1-phenylpropyne (**B**), and $H_2dpm^{2-(3,5-diCF3Ph)}$ (**F**). No side reactions were observed.

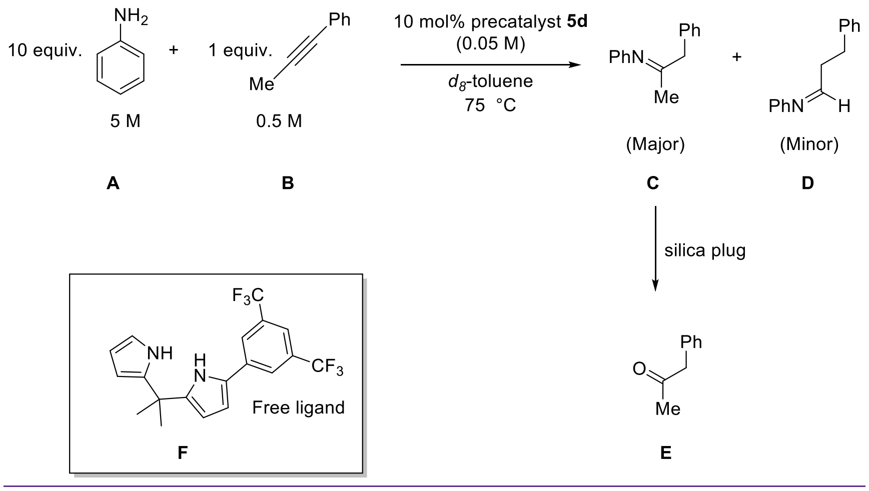

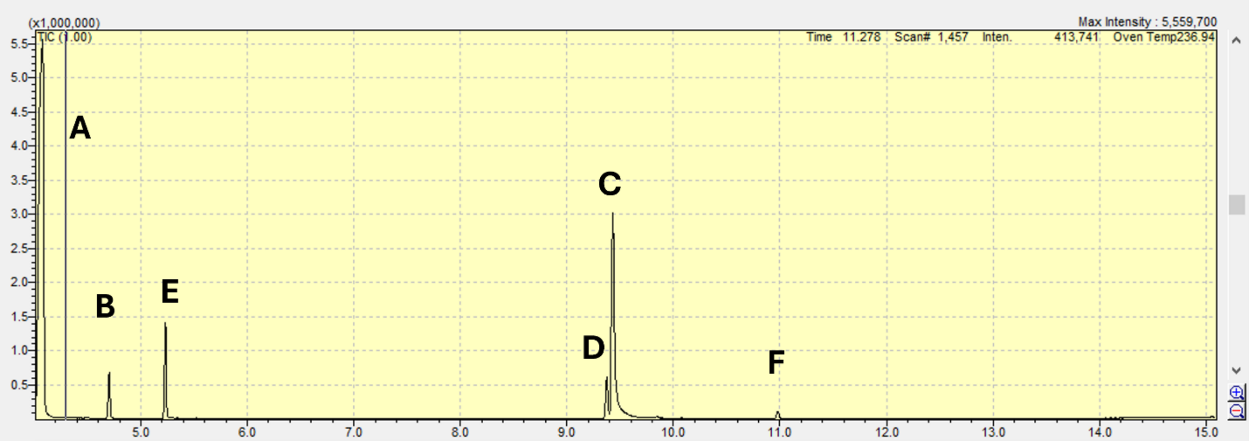

Figure S8: (Top)Possible products from 1-phenylpropyne and aniline hydroamination. (Bottom) Gas Chromatograph from the crude reaction mixture.

Ligand η¹-η⁵ Isomerization

Previously our group has done a study to calculate the enthalpic barrier for dipyrrolylmethane (dpm) isomerization.[9] We did a similar study here to understand the energetic preference for substituted pyrrole binding in an η^5 fashion. Theoretically, there are three possible structures **A**, **B**, and **C** of the catalyst under consideration (Figure S9). We used Density Functional Theory to calculate the energies with B3PW91/def2TZVP. The transition states corresponding from **A** to **B** and **B** to **C** are represented as **TS1**‡ and **TS2**‡, respectively; these were found for catalyst **5d**. The ground state structures **A**, **B**, and **C** were located for all the complexes with unsymmetrical bidentate ligands. Based on DFT calculations, isomer **A** is more stable in most cases and consistent with crystal structures, i.e., the substituted pyrrolyl side being η^5 is energetically preferred. In all cases with indoles, this ligand is strongly preferred to be η^1. The calculated energy profile for all catalysts is shown in Figure S9 to compare the relative stability of each isomer with respect to **A** (set to zero). Unlike other catalysts, **5b** shows isomer **B** has lower energy than isomer **A** and that could be attributed to the 2-Me groups on both sides, but such η^1,η^1-dpm derivatives have been observed in other cases, usually with the metal picking up an additional donor ligand.[7]

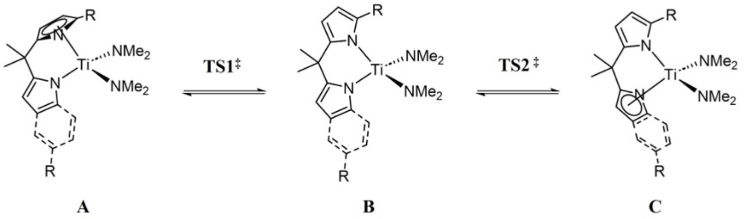

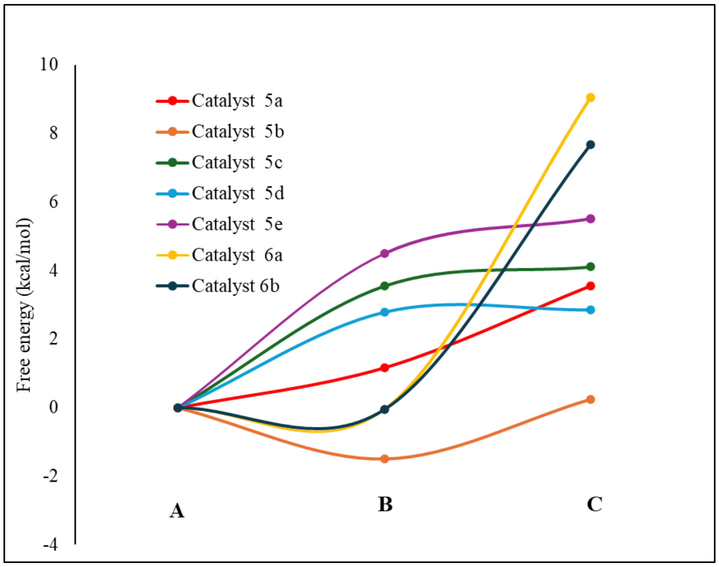

*Figure S9: (Top)Possible structures for catalysts (Bottom) Calculated ground state energies for isomers **A**, **B**, and **C** for all catalysts.*

We did further analysis of our fastest catalyst Ti(pyr$^{3,5\text{-}CF3Ph}$-C(CH_3)$_2$-pyr)(NMe_2)$_2$ **5d** by finding transition states corresponding to interconversion between each of the isomers. We found isomer **A** has the lowest energy as expected, η^5-substituted pyrrolyl. The energy barrier **TS1**‡ to go from isomer **A** (**5d**) to isomers **B** (**5d**) is 3.64 kcal/mol. This energy gap can be easily overcome at room temperature, which is consistent with NMR spectroscopy (*vide infra*). The transition state energy **TS2**‡ is higher than **TS1**‡ suggesting the conversion of isomer **B** to **A** is energetically more favorable than the conversion of isomer **B** to **C** (Figure S10). The η^1,η^1-isomer (**B**) and the η^5,η^1-isomer where the unsubstituted pyrrolyl is coordinated through the π-system (**C**) have the same energy (2.8 kcal/mol) according to DFT.

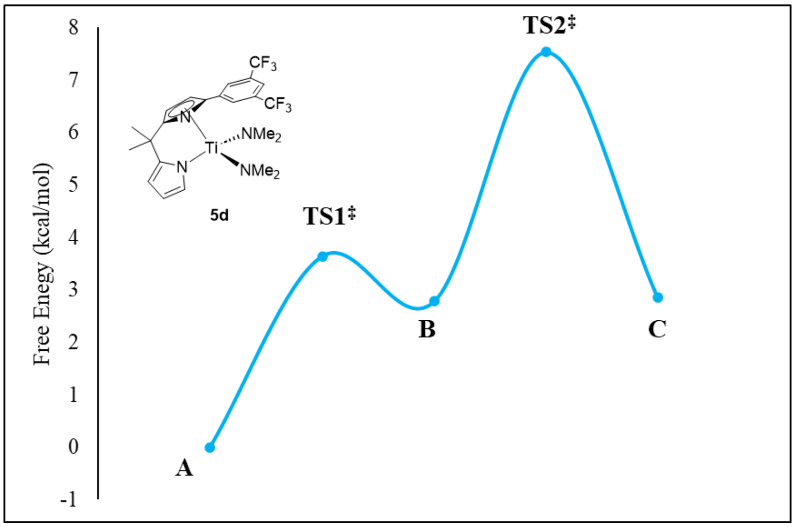

*Figure S10: Calculated energy profile for catalyst Ti(pyr$^{3,5\text{-}CF3Ph}$-C(CH_3)$_2$-pyr)(NMe_2)$_2$ **5d** showing interconversion between each isomer (A, B, and C) and associated transition states (TS1‡ and TS2‡).*

Variable temperature NMR spectroscopy was used to further analyze the η^1-η^5 ligand isomerism of Ti(pyr$^{3,5\text{-}CF3Ph}$-C(CH_3)$_2$-pyr)(NMe_2)$_2$ **5d** complex. 0.03 M solution of **5d** was prepared in the J-young NMR tube. Proton NMR was collected between –92 °C to 25 °C (Figure S11). Temperature calibration was done using a methanol sample in the NMR probe. At 182 K, we believe the ground state structure should be similar to crystal structure, where substituted pyrrole is coordinated in η^5-fashion to the metal center. In such case, the two methyl groups present on the ligand backbone will be in different chemical environment, and show two singlets in the NMR spectrum (boxed region in Figure S11). As we increase the temperature to 208 K, the coalescence temperature, the two peaks start to broaden and merge into a broad peak with a flat top. Warming the sample to 232 K gives a sharp signal for the methyl groups suggesting fast exchange on the NMR timescale.

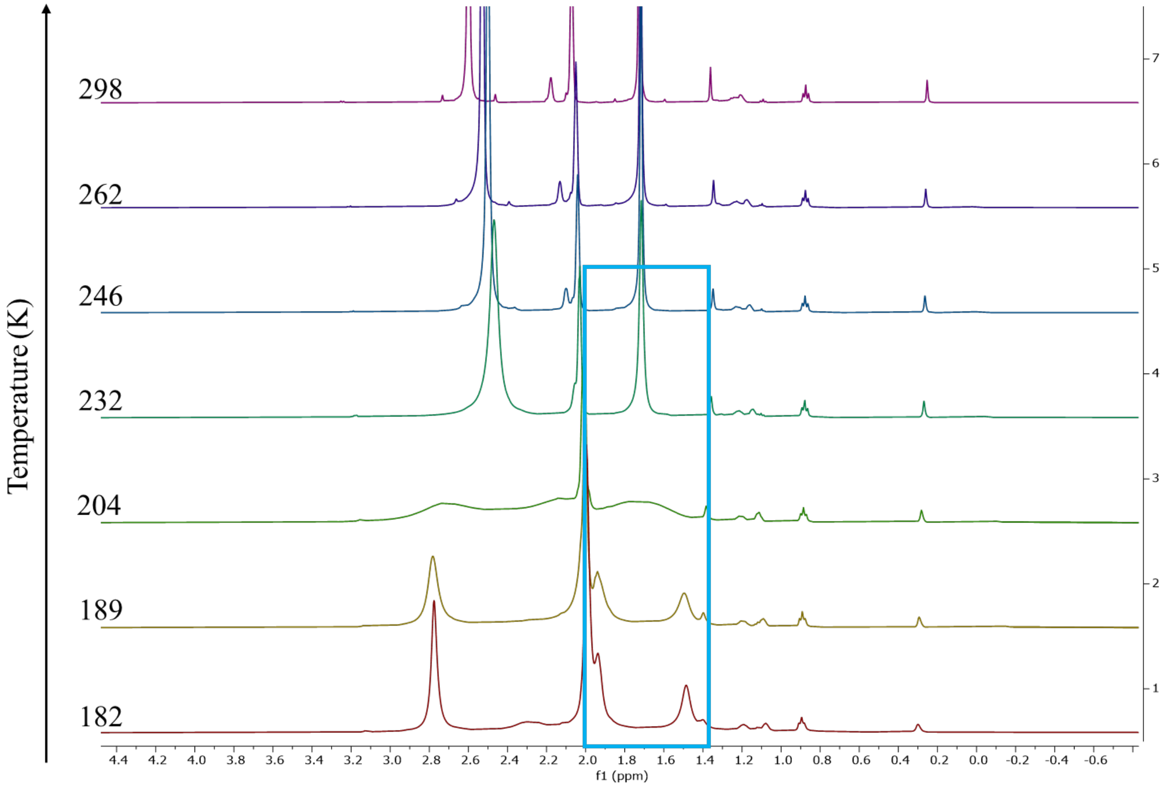

Figure S11: Variable temerpature NMR study for catalyst Ti(pyr$^{3,5\text{-}CF3Ph}$-C(CH$_3$)$_2$-pyr)(NMe$_2$)$_2$ ***5d***

Line shape analysis[10] was used to calculate the rate constant associated with different temperatures. At 182 K the rate constant k1 is given by Eq. S1; where $\Delta w = w - w_o$, w is the width at the half height of the broadened peak (189 K), and w_o is the width of the half-height at 182 K. At the coalescence temperature, 204 K, the rate constant k2 is given Eq. S2; where ϑ_a and ϑ_b are chemical shifts of the different methyl protons in Hz. At 232 K, the rate constant k3 is calculated by using Eq. S3; where w_f is the half height of the peak at the fast exchange limit.

$$k1 = \pi(\Delta w) \quad \textbf{Eq. S1}$$

$$k2 = \frac{\pi(\vartheta_a - \vartheta_b)}{\sqrt{2}} \quad \textbf{Eq. S2}$$

$$k3 = \frac{\pi(\vartheta_a - \vartheta_b)^2}{2(w - w_f)} \quad \textbf{Eq. S3}$$

Using Eyring Equation (Eq. S4), the slope and intercept of ln(k_{obs}/T) vs 1/T plot was used to calculate enthalpy and entropy associated with η^1-η^5 ligand isomerism.

$$ln\left(\frac{k_{obs}}{T}\right) = -\frac{\Delta H^{\ddagger}}{RT} + ln\frac{k_B}{h} + \frac{\Delta S^{\ddagger}}{R} \quad \textbf{Eq S4}$$

**Temperature (K)**	**Rate (s^{-1})**	**1/T (10^{-3})**	**ln(k/T)**
181.9	20.2	5.498	-2.199
204.7	499	4.886	0.890
246.0	25,600	4.310	4.704

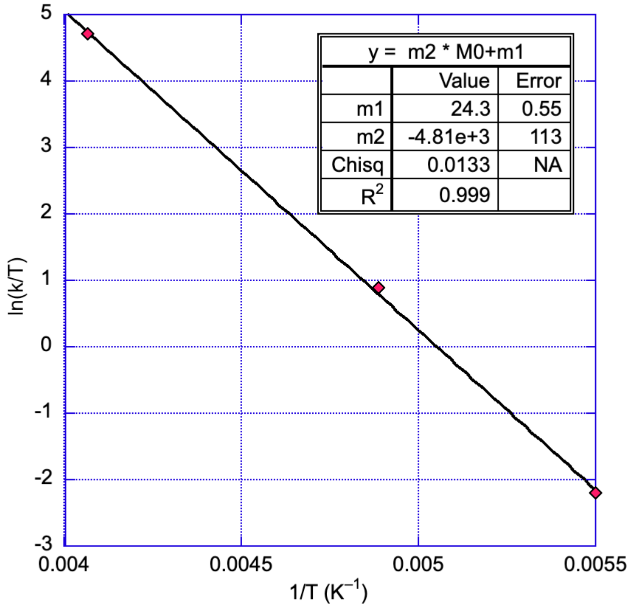

The enthapic barrier, $\Delta H^{\ddagger}$, for η^1-η^5 ligand isomerism of catalyst **5d** = 9.5 ± 0.2 kcal/mol, and $\Delta S^{\ddagger}$ = 1.2 ± 1.1 cal/mol. The Gibbs free energy, $\Delta G^{\ddagger}$, at room temperature is 9.1 ± 0.6 kcal/mol. The lower barrier between **A** and **B** suggests that these will be quickly exchanging at the accessible temperatures on the NMR timescale. As a result, we assume we are measuring the barrier (**TS2**‡) between **C** and the fast exchanging **B**/**A** system.

Modeling of the Kinetic Data

The data used in the modeling is in Table S1 below. The LDP and %V_{bur} values are from our previous studies. The rate constants for complexes **1-4** were previously published.[7] The parameters for new compounds (**5-6**) were all known from the previous study. For the unsymmetrical **5-6** complexes, "side 1" is simply the side of the ligand with the larger LDP value. Using the larger %V_{bur} as "side 1" does not give as good a model, statistically speaking.

Table S1. Data for the Modeling of Natural Variables.

Complex	LDP1	LDP2	%V_{bur}1	%V_{bur}2	k_{obs} x 10^4 (s^{-1})	k_{calc}	Abs diff
**1a**	13.64	13.64	20.4	20.4	4.16	4.11	0.05
**3a**	12.49	12.49	22.6	22.6	0.66	0.60	0.06
**1b**	13.46	13.46	23.7	23.7	1.35	1.66	0.31
**1c**	14.03	14.03	27.1	27.1	0.52	0.51	0.01
**1d**	13.91	13.91	26.7	26.7	0.55	0.55	0.00
**1e**	14.32	14.32	27.9	27.9	0.58	0.53	0.05
**3b**	12.66	12.66	22.6	22.6	1.08	0.91	0.17
**4**	11.98	11.98	21.6	21.6	0.43	0.32	0.11
**2a**	11.87	11.87	21.5	21.5	0.24	0.18	0.06
**2b**	11.82	11.82	21.5	21.5	0.05	0.09	0.04
**5a**	13.64	13.46	20.4	23.7	3.10	2.69	0.41
**5b**	13.46	13.09	23.7	23.1	1.90	2.32	0.42
**5c**	14.03	13.64	27.1	20.4	3.95	4.14	0.19
**5d**	14.32	13.64	27.9	20.4	5.50	4.82	0.68
**5e**	13.91	13.64	26.7	20.4	3.46	3.86	0.40
**6a**	13.64	12.49	20.4	22.6	3.64	4.18	0.54
**6b**	13.64	12.22	20.4	23.3	4.40	4.10	0.30
**u(i)**	13.07	13.07	24.15	24.15			
**Delta u(i)**	1.25	1.25	3.75	3.75		Ave. diff =	0.22

The parameters of the multivariate analysis were found using Microsoft Excel (16.78.3). The output is shown below.

Regression Statistics	
Multiple R	0.98494985
R Square	0.97012621
Adjusted R Square	0.96016828
Standard Error	0.35649998
Observations	17

ANOVA

	df	*SS*	*MS*	*F*	*Significance F*
Regression	4	49.5265641	12.381641	97.4224799	4.8481E-09
Residual	12	1.52510686	0.12709224		
Total	16	51.051671			

	Coeff.	*Standard Error*	*t Stat*	*P-value*	*Lower 95%*	*Upper 95%*
a	-7.58	1.57	-4.84	0.000408	-10.99	-4.165
b	2.80	0.211	13.3	1.525E-08	2.344	3.262
c	-0.98	0.264	-3.734	0.00287	-1.561	-0.410
d	-0.159	0.0443	-3.59	0.00374	-0.255	-0.062
e	-0.484	0.0421	-11.48	7.94E-08	-0.575	-0.392

The fits were done using both the "natural" variables, the direct LDP and $\%V_{bur}$ values, and scaled variables. The natural variables give a model that can be used to calculate the rate constant of a new catalyst if the LDP and $\%V_{bur}$ values are known or can be accurately estimated. The scaled variables allow direct comparison between the different coefficients and comparison of electronic and steric factors.

Below is a plot of the calculated (from the model) rate constants vs the observed rate constants. Obviously, the plot should have a slope of 1 and has a slope of 0.99 ± 0.03. The R^2 is 0.97 for this fit.

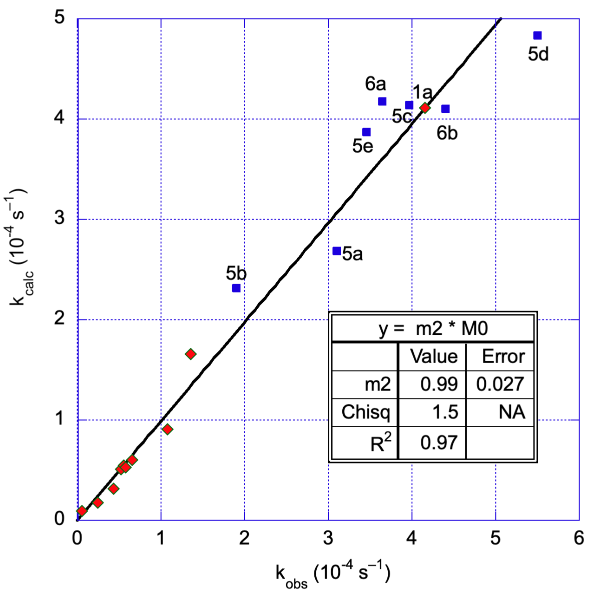

Errors Analysis Using Unweighted Rate Regression as Example

The error analysis from the regression by Microsoft Excel is shown above, and the "by hand" analysis gives the same result, which is shown here. In Table S1 are the experimentally obtained rate constants, the expected responses from the model (k_{calc}), and the absolute values of the difference (residuals). The sum of the squares of the residuals (SSR) is found by:

$$SSR = \sum_{i=1}^{n} (y_i - \hat{y}_i)^2 = 1.53$$

where y_i = response (rate constant) and $\hat{y}_i$ = the expected response from the model, which for our data here is 1.53. (The units in the error analysis will be 10^{-4} s^{-1} for all the values, just like the rate constants in the table.) The Mean Residual Sum of Squares (MSR) is found by dividing the SSR by the degrees of freedom.

$$MSR = \frac{SSR}{DoF} = 0.127$$

The degrees of freedom for this calculation are the number of data points (n) minus the number of parameters (p), which in this case is DoF = $n - p$ = 17 – 5 = 12, and the 1.53/12 = 0.127. The Total Sum of

Squares (TSS) is the sum of the difference between all the responses (rate constants in this case) and the mean value for the rate constants ($\bar{y}$):[11]

$$TSS = \sum_{i=1}^{n} (y_i - \bar{y})^2 = 51.1$$

The Total Mean Sum of Squares is then TSS the degrees of freedom for this calculation, which is $n - 1 = 16$, so

$$MST = \frac{TSS}{DoF} = \frac{51.1}{16} = 3.19$$

The Explained Sum of Squares (ESS) is TSS minus the SSR:

$$ESS = TSS - SSR = 51.1 - 1.53 = 49.5$$

The ratio of ESS and TSS is R^2:

$$R^2 = \frac{ESS}{TSS} = \frac{49.5}{51.1} = 0.970$$

which is precisely the R^2 value found by linear regression of a plot of k_{calc} vs k_{obs} (vide supra).

The standard error is calculated from the model matrix (**X**), which is the design matrix (**D**) with a column of "1" in front. The matrix **X** is simple as shown below.

1	13.64	13.64	20.4	20.4
1	12.49	12.49	22.6	22.6
1	13.46	13.46	23.7	23.7
1	14.03	14.03	27.1	27.1
1	13.91	13.91	26.7	26.7
1	14.32	14.32	27.9	27.9
1	12.66	12.66	22.6	22.6
1	11.98	11.98	21.6	21.6
1	11.87	11.87	21.5	21.5
1	11.82	11.82	21.5	21.5
1	13.64	13.46	20.4	23.7
1	13.46	13.09	23.7	23.1
1	14.03	13.64	27.1	20.4
1	14.32	13.64	27.9	20.4

1	13.91	13.64	26.7	20.4
1	13.64	12.49	20.4	22.6
1	13.64	12.22	20.4	23.3

From the matrix above, the dispersion matrix is calculated, $(\mathbf{X}^{\mathbf{T}}\mathbf{X})^{-1}$, which is multiplied by the variance. The variance (σ^2) is the SSR/DoF = MSR = 0.127. As a result, the standard errors (s_i) are the square roots of the diagonal elements in the matrix $(\mathbf{X}^{\mathbf{T}}\mathbf{X})^{-1}\,\sigma^2$. In this case, with the natural variables in the **X** matrix above $(\mathbf{X}^{\mathbf{T}}\mathbf{X})^{-1}\,\sigma^2$ =

**2.4565182**	-0.0694428	-0.1285479	0.01899512	-0.0126794
-0.0694428	**0.04440677**	-0.0430437	0.00074517	0.00097475
-0.1285479	-0.0430437	**0.06970082**	-0.005775	-0.0031515
0.01899512	0.00074517	-0.005775	**0.00196051**	9.4101E-06
-0.0126794	0.00097475	-0.0031515	9.4101E-06	**0.0017752**

with the diagonal elements for the standard errors in bold. The standard errors for the parameters are then the square root of the diagonal elements of the matrix:

	Coefficients	*Standard Error*
a	-7.5794107	$\sqrt{2.4565} = 1.57$
b	2.802784419	$\sqrt{0.444} = 0.211$
c	-0.985275006	$\sqrt{0.0697} = 0.264$
d	-0.158790778	$\sqrt{0.00196} = 0.0443$
e	-0.483539354	$\sqrt{0.001775} = 0.042$

To find the confidence limits in Table 1 in the manuscript, the standard errors were multiplied by the t_{crit} values for the 95% confidence limit and DoF. (The DoF in this calculation is given by $n - p$, which in this case is 17 – 5 = 12.) The t_{crit} value for DoF = 12 is 2.179.[12]

Modeling with the Ligand Site Determined by Sterics

We also did the modeling with "side 1" determined by the $\%V_{bur}$ value. The tabulated data then become:

**Complex**	**LDP1**	**LDP2**	**$\%V_{bur}1$**	**$\%V_{bur}2$**	**k_{obs} x 10^4 (s^{-1})**	**k(calc)**	**Abs diff**
**1a**	13.64	13.64	20.4	20.4	4.16	3.82	0.34
**3a**	12.49	12.49	22.6	22.6	0.66	0.67	0.00
**1b**	13.46	13.46	23.7	23.7	1.35	1.61	0.26
**1c**	14.03	14.03	27.1	27.1	0.52	0.56	0.04
**1d**	13.91	13.91	26.7	26.7	0.55	0.60	0.05
**1e**	14.32	14.32	27.9	27.9	0.58	0.57	0.01
**3b**	12.66	12.66	22.6	22.6	1.08	0.94	0.14
**4**	11.98	11.98	21.6	21.6	0.43	0.42	0.02
**2a**	11.87	11.87	21.5	21.5	0.24	0.29	0.05
**2b**	11.82	11.82	21.5	21.5	0.05	0.21	0.16
**5a**	13.46	13.64	23.7	20.4	3.10	3.99	0.89
**5b**	13.46	13.09	23.7	23.1	1.90	1.45	0.45
**5c**	14.03	13.64	27.1	20.4	3.95	4.30	0.35
**5d**	14.32	13.64	27.9	20.4	5.50	4.39	1.11
**5e**	13.91	13.64	26.7	20.4	3.46	4.25	0.79
**6a**	12.49	13.64	22.6	20.4	3.64	3.77	0.13
**6b**	12.22	13.64	23.3	20.4	4.40	3.77	0.63
						average	0.32

Regression Statistics	
Multiple R	0.9639
R Square	0.9290
Adjusted R Square	0.9054
Standard Error	0.5495
Observations	17

ANOVA

	df	*SS*	*MS*	*F*	*Significance F*
Regression	4	47.4284	11.8571	39.2699	0.0000
Residual	12	3.6233	0.3019		
Total	16	51.0517			

	Coefficients	Standard Error	t Stat	P-value	Lower 95%	Upper 95%
a	-6.5566	2.5051	-2.6173	0.0225	-12.0147	-1.0985
b	0.1578	0.3354	0.4705	0.6464	-0.5730	0.8887
c	1.4718	0.3100	4.7485	0.0005	0.7965	2.1471
d	0.0612	0.0963	0.6357	0.5369	-0.1485	0.2709
e	-0.6422	0.0603	-10.6450	0.0000	-0.7737	-0.5108

From these sterically-driven values, we can give a different set of parameter with their 95% confidence intervals. As shown, only two of the parameters (excluding the intercept) is above the confidence interval in this case.

Coefficients	*Steric Model with 95% confidence intervals*
a	–6.55 ± 5.46
b	0.16 ± 0.73
c	1.47 ± 0.68
d	0.061 ± 0.21
e	–0.64 ± 0.13

The statistics for this sterically-driven case are significantly worse. For example, the R^2 here is 0.93, compared to $R^2 = 0.97$ for the case above where the electronics are determining the ligand position.

Modeling of the Scaled Values

The scaling was done using the equation below, where x_i = scaled variable, u_i = natural variable, u_i^0 = midpoint of the range of the natural variables, and Δu_i = the difference between the midpoint and the high value (half the full range).

$$x_i = \frac{u_i - u_i^0}{\Delta u_i}$$

The equations for the calculation of u_i^0 and Δu_i are shown below.

$$\Delta u_i = u_i^{high} - u_i^0$$

$$u_i^0 = \frac{u_i^{high} + u_i^{low}}{2}$$

The values in Table S1 were scaled (–1 to +1) in this way and are shown in Table S2. A comparison of the scaled and natural parameters can be found in Table 1 in the manuscript.

In order to determine if the electronic parameter (LDP) or the steric parameter ($\%V_{bur}$) determined the position of the ligand in the key transition state, we also modelled the data where the larger $\%V_{bur}$ determined ligand one. The statistics for this model are somewhat worse than if the electronics are used to determine the ligand label, suggesting the electronics of the ligand determine the orientation in the key transition state.

Table S2. Data for the Modelling with Scaled Parameters.

**Complex**	**LDP1**	**LDP2**	**$\%V_{bur}1$**	**$\%V_{bur}2$**	**k_{obs} x 10^4 (s^{-1})**	**k_{calc}**
**1a**	0.456	0.456	-1	-1	4.16	4.11
**3a**	-0.464	-0.464	-0.4133333	-0.4133333	0.66	0.60
**1b**	0.312	0.312	-0.12	-0.12	1.35	1.66
**1c**	0.768	0.768	0.78666667	0.78666667	0.52	0.51
**1d**	0.672	0.672	0.68	0.68	0.55	0.55
**1e**	1	1	1	1	0.58	0.53
**3b**	-0.328	-0.328	-0.4133333	-0.4133333	1.08	0.91
**4**	-0.872	-0.872	-0.68	-0.68	0.43	0.32
**2a**	-0.96	-0.96	-0.7066667	-0.7066667	0.24	0.18
**2b**	-1	-1	-0.7066667	-0.7066667	0.05	0.09
**5a**	0.456	0.312	-1	-0.12	3.10	2.69
**5b**	0.312	0.016	-0.12	-0.28	1.90	2.32
**5c**	0.768	0.456	0.78666667	-1	3.95	4.14
**5d**	1	0.456	1	-1	5.50	4.82
**5e**	0.672	0.456	0.68	-1	3.46	3.86
**6a**	0.456	-0.464	-1	-0.4133333	3.64	4.18
**6b**	0.456	-0.68	-1	-0.2266667	4.40	4.10

Regression Statistics	
Multiple R	0.96
R Square	0.93
Adjusted R Square	0.91
Standard Error	0.55

Observations	17

ANOVA

	df	*SS*	*MS*	*F*	*Significance F*
Regression	4	47.43	11.86	39.27	8.40E-07
Residual	12	3.62	0.30		
Total	16	51.05			

	Coeff.	*Standard Error*	*t Stat*	*P-value*	*Lower 95%*	*Upper 95%*
a	-6.56	2.51	-2.62	0.02	-12.01	-1.10
b	0.16	0.34	0.47	0.65	-0.57	0.89
c	1.47	0.31	4.75	0.00	0.80	2.15
d	0.06	0.10	0.64	0.54	-0.15	0.27
e	-0.64	0.06	-10.65	1.82E-07	-0.77	-0.51

Weighting of Reaction Rates: Box-Cox Analysis

The analysis above of raw rate constants from the experiments suffers from a shortcoming. One assumption of the statistical analysis is that the error is normally distributed. This can be checked by plotting the residuals vs the calculated rate constants. The residuals (e) are found using:

$$e_i = y_i - \hat{y}_i$$

where $\hat{y}_i$ = the calculated value of the response (rate constant) from the model. A plot of these residuals vs the calculated rate constants is shown below. Notice how the data is "funnel-shaped" with larger residuals for the larger rate constants. This suggests that the data are not normally distributed, and our error is increasing with increasing rate constant. In other words, accurate measurement is becoming more difficult as the rates get faster. This is not uncommon in data derived from sources like NMR spectroscopy, as not all peak integrations have the same error with peak size, for example.[12] Regardless of the cause, this plot suggests one of the underlying assumptions of the statistical analysis used is invalid. (Plots very similar to this one are found in several texts on statistics as data that needs to be weighted; for example, see page 63 of Draper and Smith 3rd Ed[13] and page 154 of Carlson and Carlson 2nd Ed.[12])

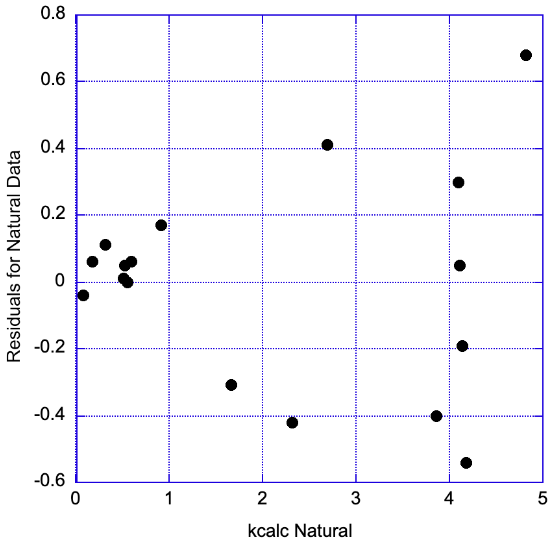

To address this problem, one can weight the data; for example, the fit can be done on the natural log or inverse of the rate constant rather than the rate constant itself. A detailed procedure for getting a normally distributed response for multivariate analysis was developed by Box and Cox.[12, 14-15] The procedure uses Eq 2 in the manuscript to weight the data. The parameter λ is varied (we examined 1 to –1) and is plotted vs the natural log of RSS of the resulting model. The plot for our data is shown below, which suggests that λ ~ 0.7 is a minimum.

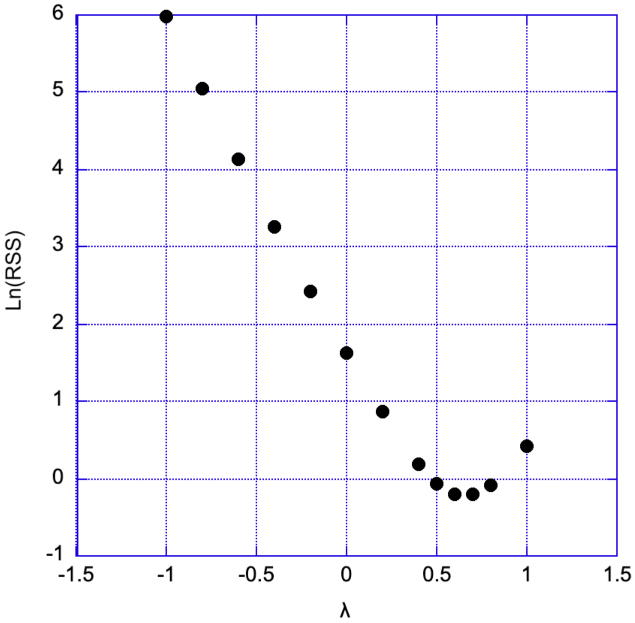

Using this weighting scheme on the responses, one can see that the residuals are more symmetrically distributed around zero across the calculated rate constants.

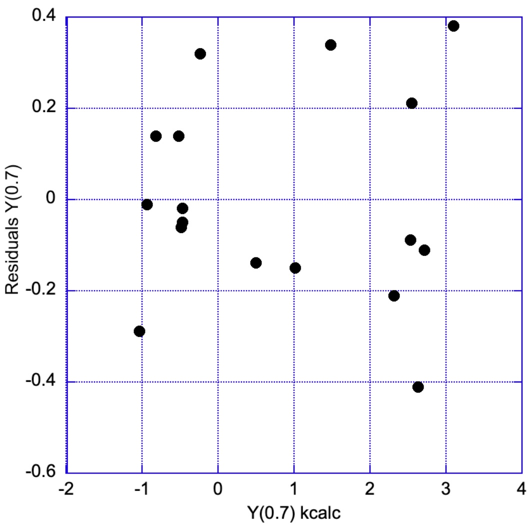

In addition, the use of responses with normally distributed error resulted in somewhat better statistics for the regression and smaller standard errors on the parameters, which are shown in Table 1 in the article.

The sterically-driven model was also carried out with the weighted responses as well. Just as with the electronically-driven model, the statistics improved slightly, with an $R^2 = 0.94$ (c.f., $R^2 = 0.93$ for the unweighted data). Also just as before, the steric-determined model is quite poor relative to the electronic model. The parameters with 95% confidence level intervals from the regression on the weighted data are shown below.

Coefficients	*Steric Model from weighted responses with 95% confidence intervals*
a	–7.95 ± 4.33
b	0.19 ± 0.58
c	1.37 ± 0.54
d	0.016 ± 0.166
e	–0.55 ± 0.10

NMR Spectra for Ligands and Titanium Catalysts

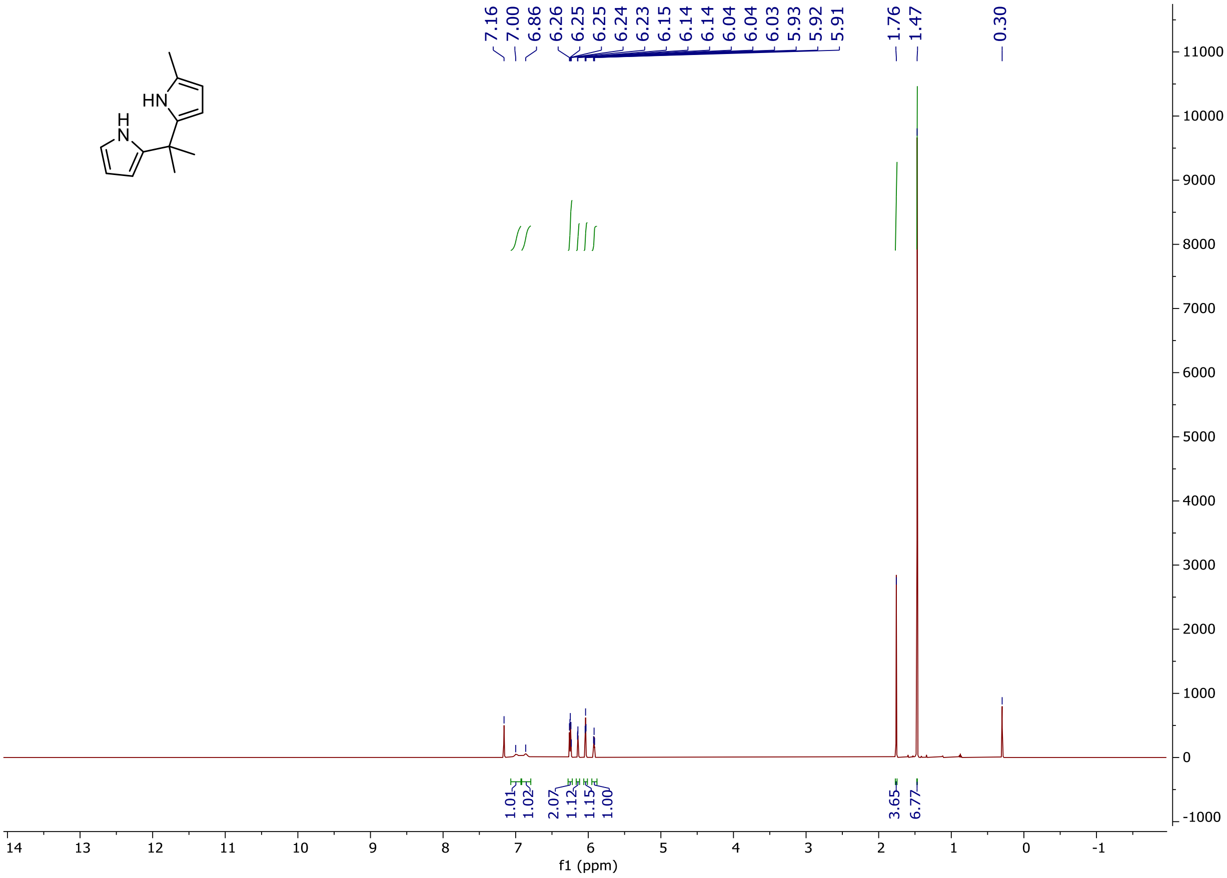

Figure S12. ^{1}H NMR of $H_2dpm^{2\text{-}Me}$

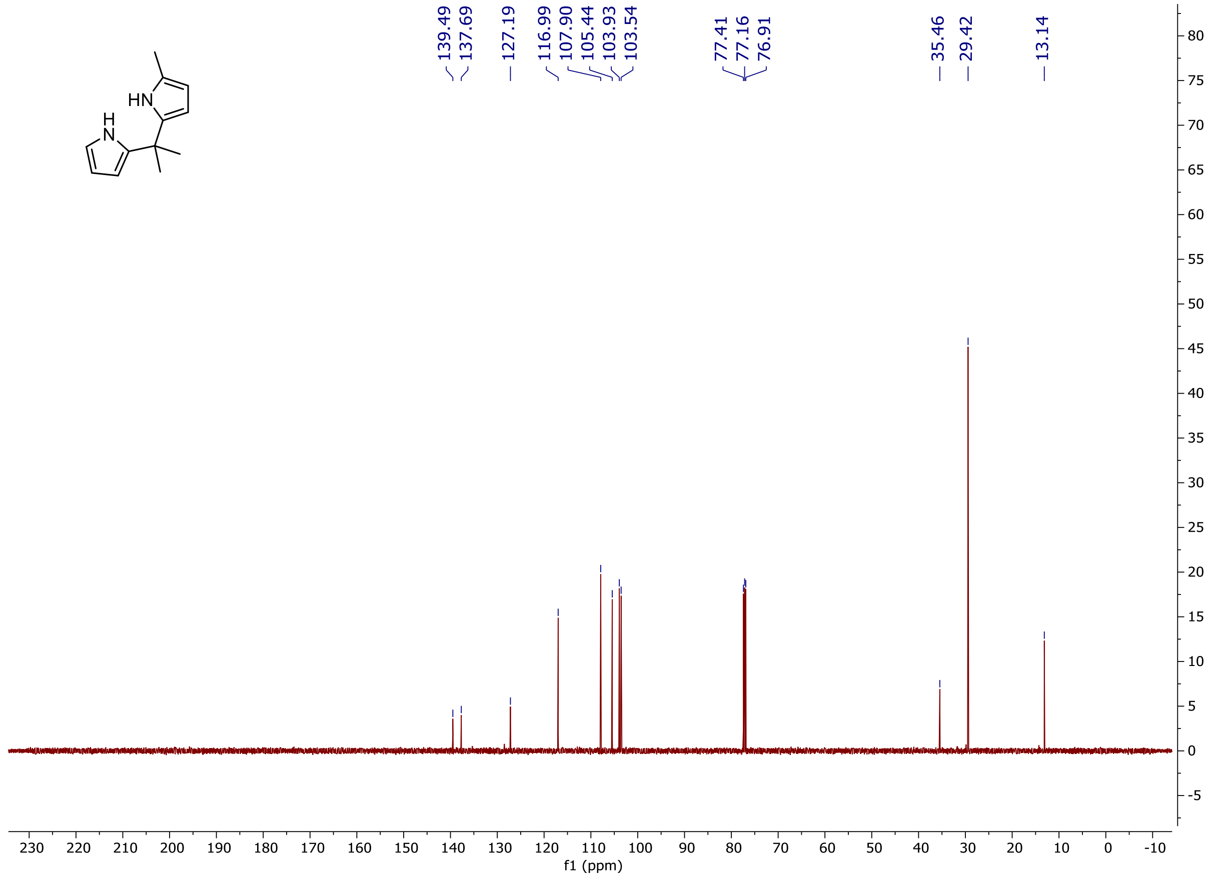

Figure S13. ^{13}*C NMR of* $H_2dpm^{2\text{-}Me}$

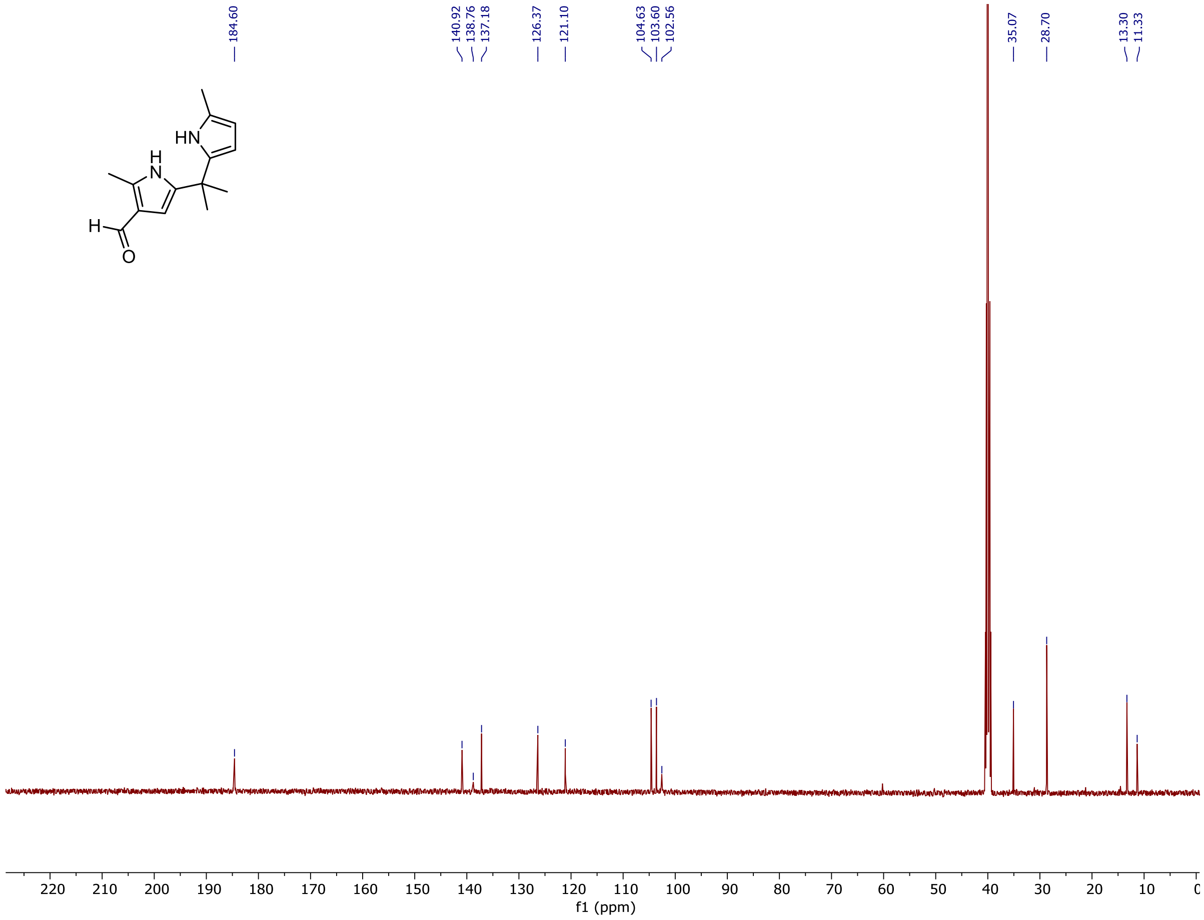

Figure S14. ^{13}C *NMR of* $H_2dpm^{2\text{-}Me}$

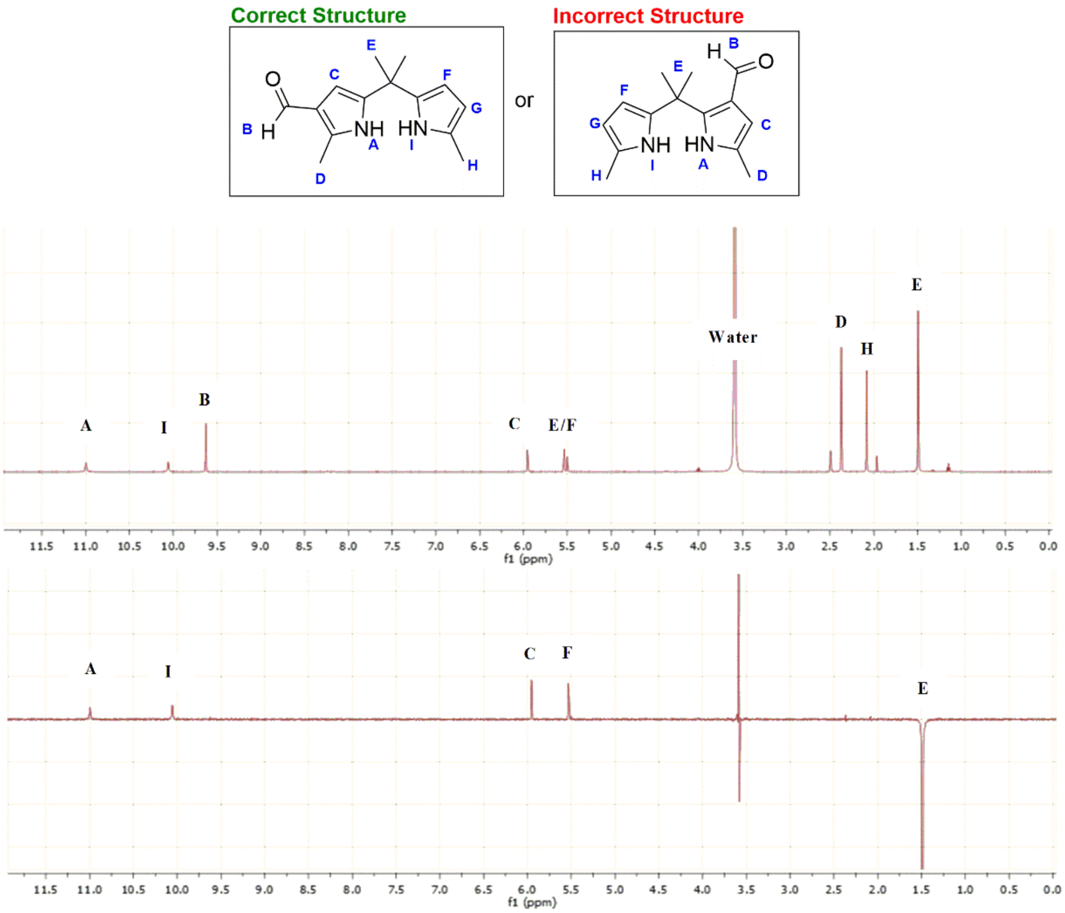

Figure S15. *^{1}H-NMR (top) and 1D-NOESY (bottom) spectra of $H_2dpm^{2,2'\text{-}DiMe\text{-}3\text{-}DiCHO}$*

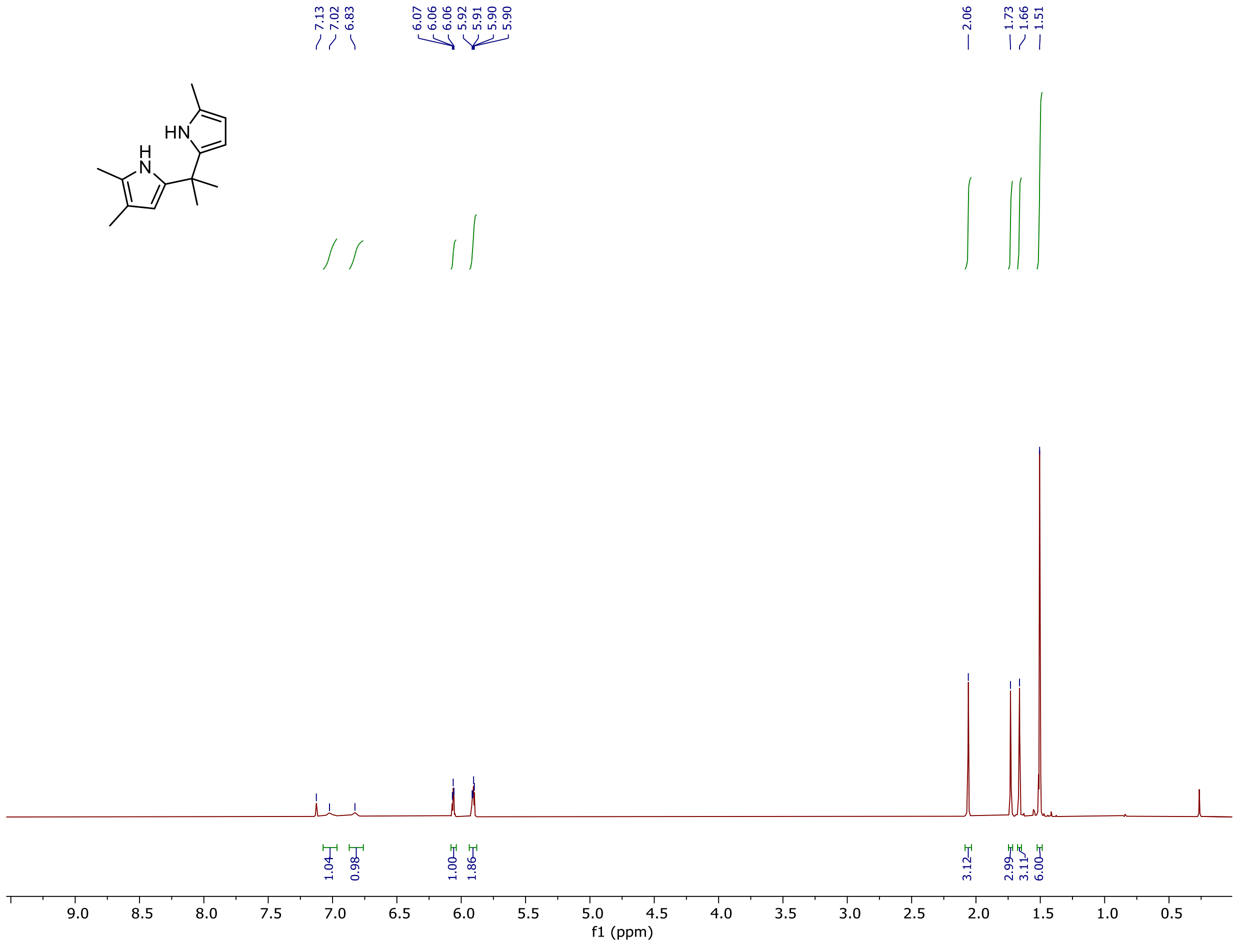

Figure S16. ^{1}H NMR of $H_2dpm^{2,2',3\text{-}TriMe}$

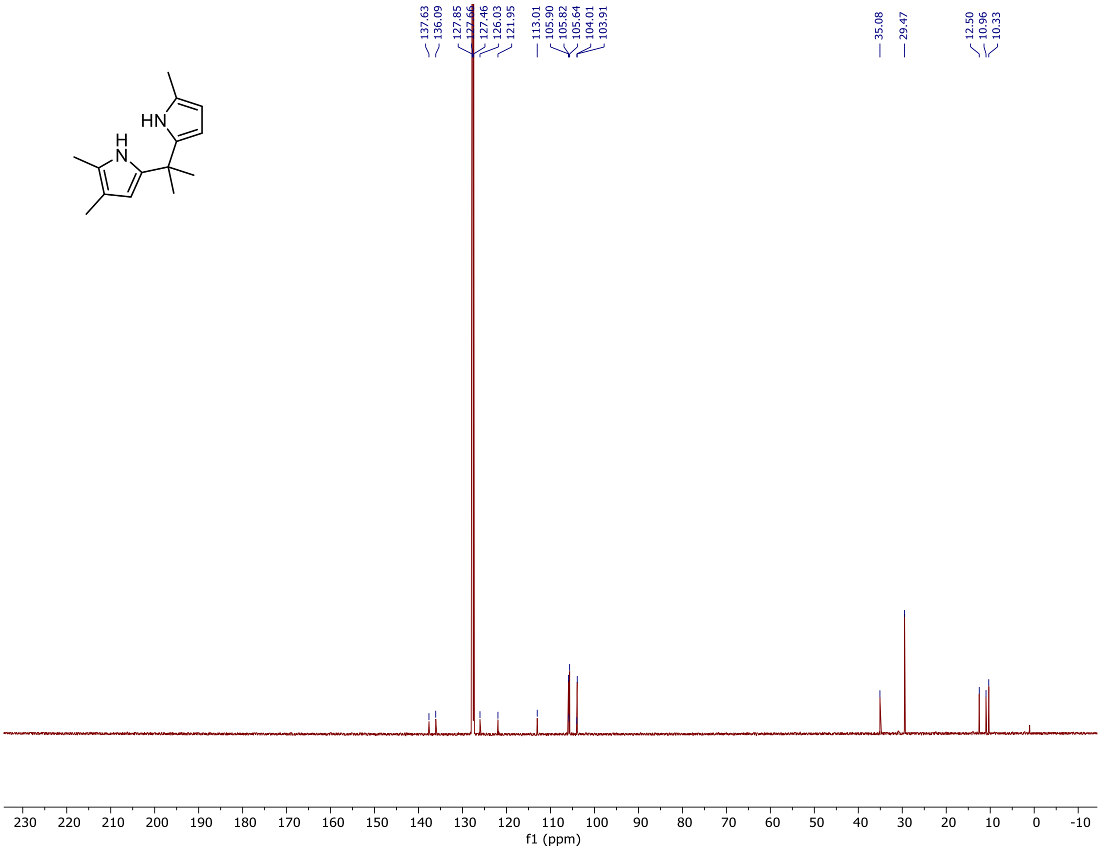

Figure S17. ^{13}C NMR of $H_2dpm^{2,2',3\text{-}TriMe}$

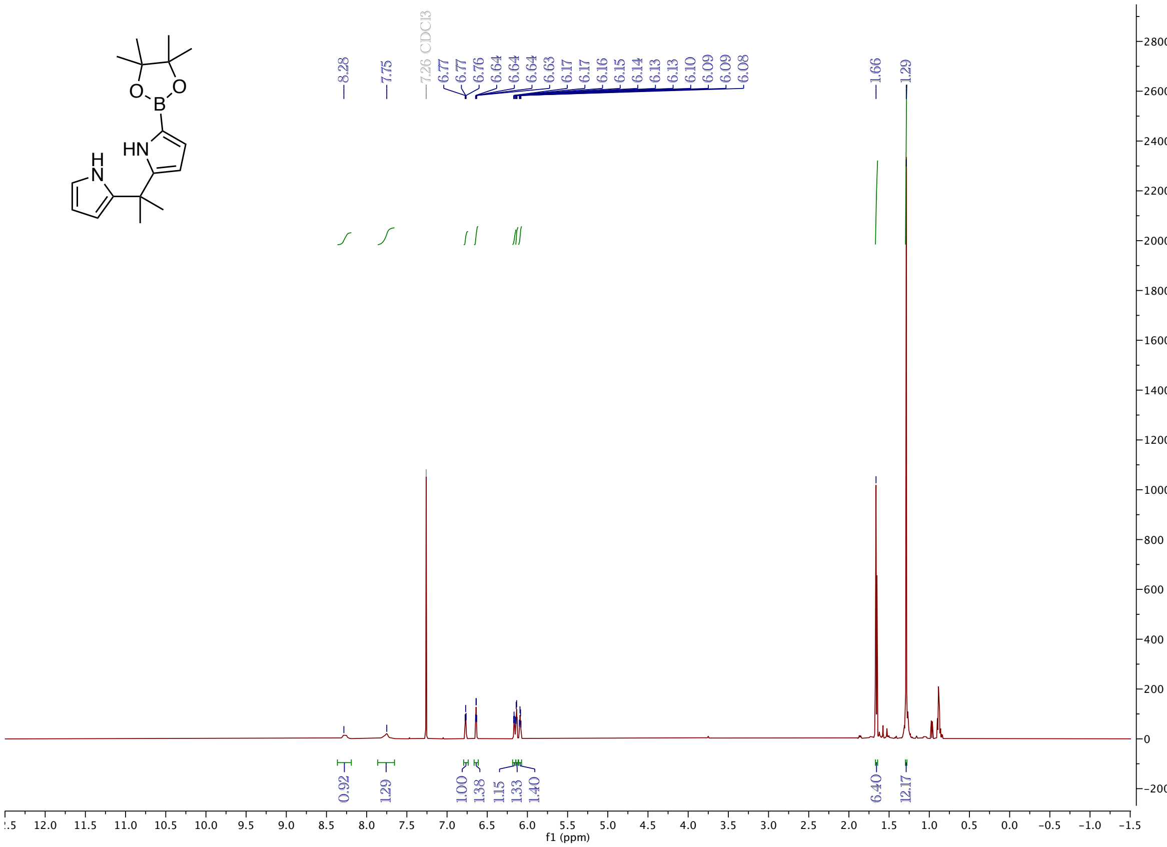

Figure S18. *^{1}H NMR of $H_2dpm^{2\text{-}Bpin}$*

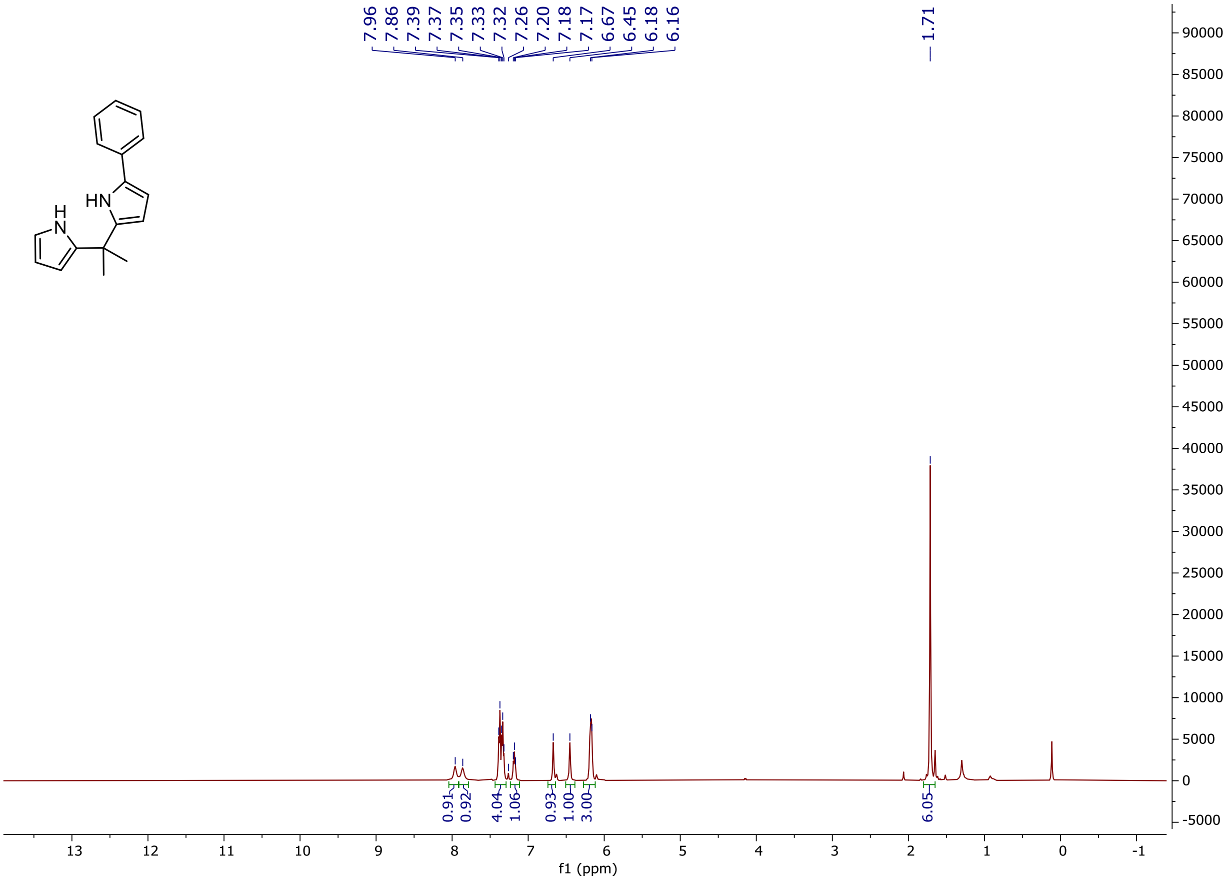

Figure S19. ¹H NMR of $H_2dpm^{2\text{-}Ph}$

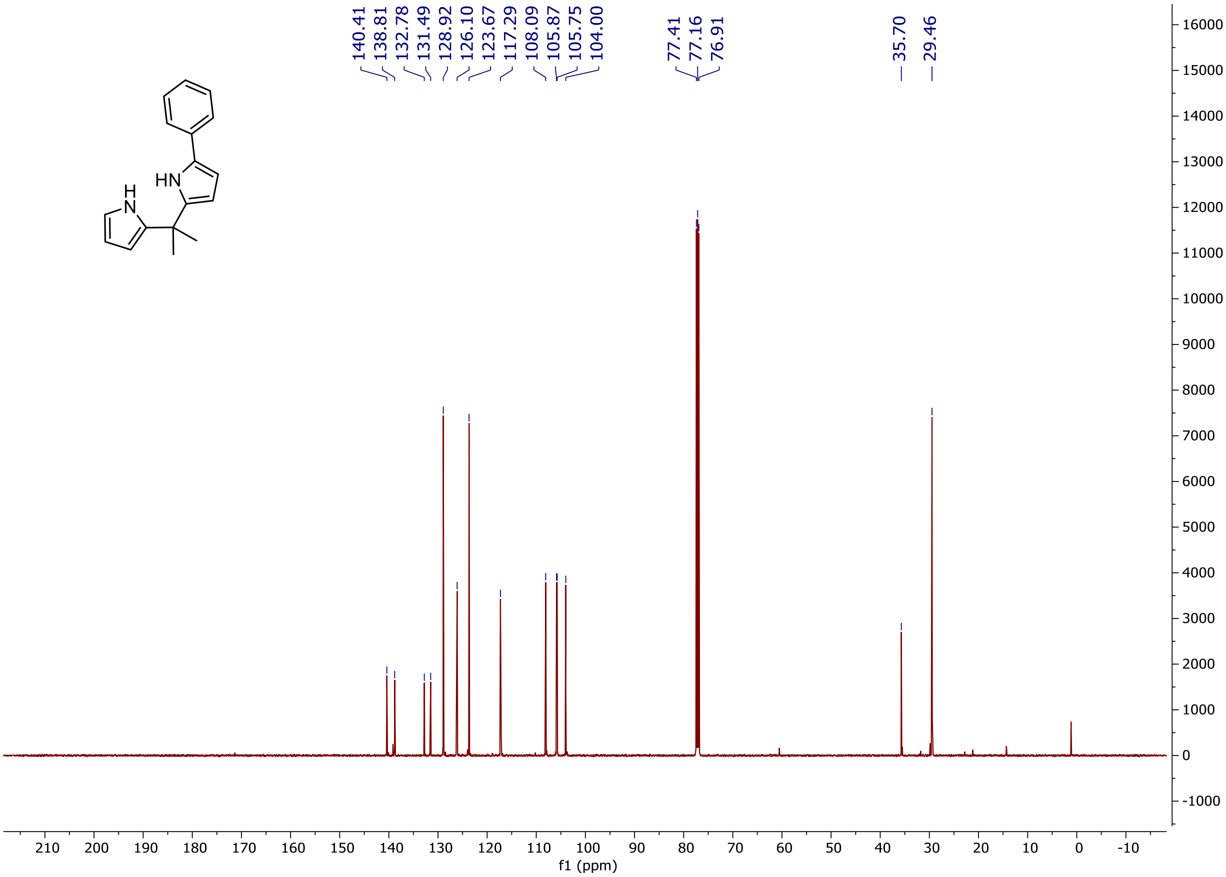

Figure S20. ^{13}C NMR of $H_2dpm^{2\text{-}Ph}$

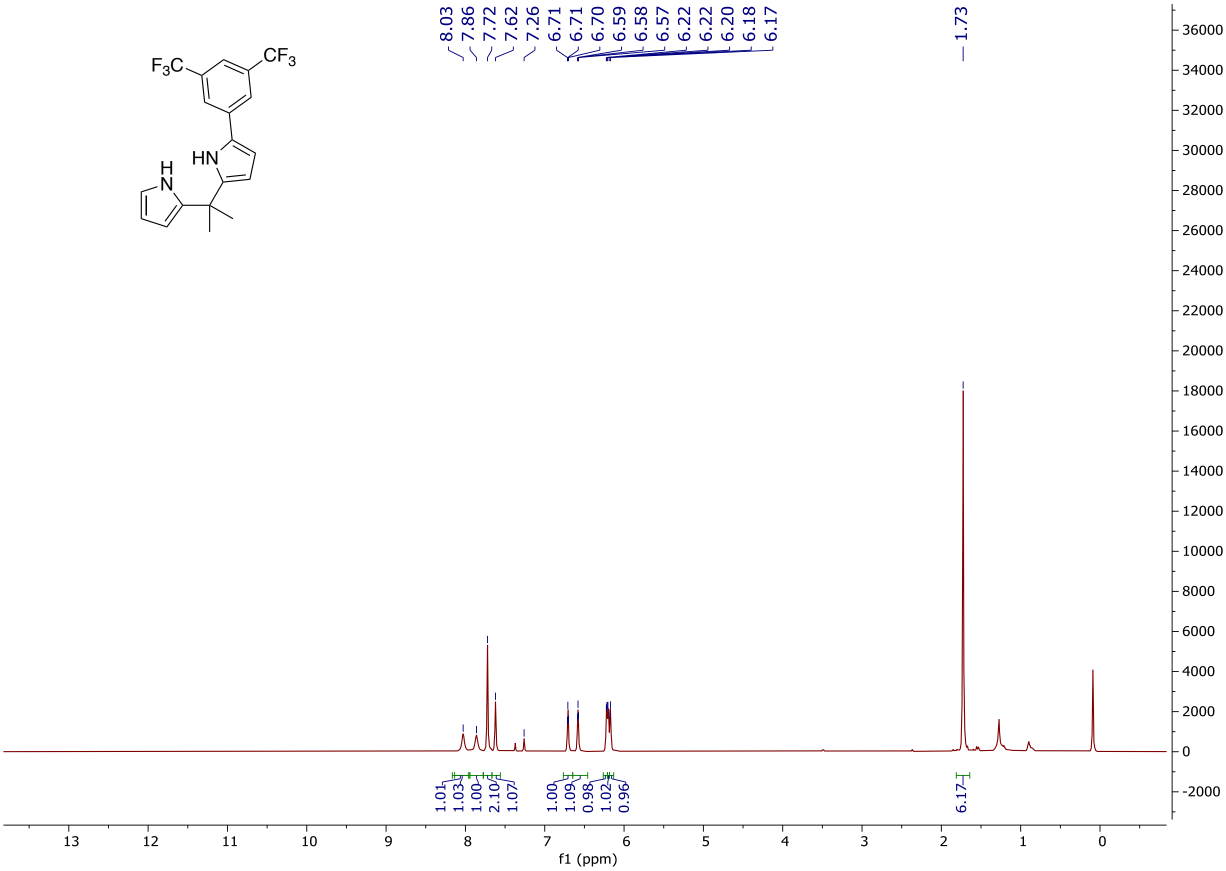

Figure S21. ¹H NMR of $H_2dpm^{2\text{-}(3,5\text{-}diCF3Ph)}$

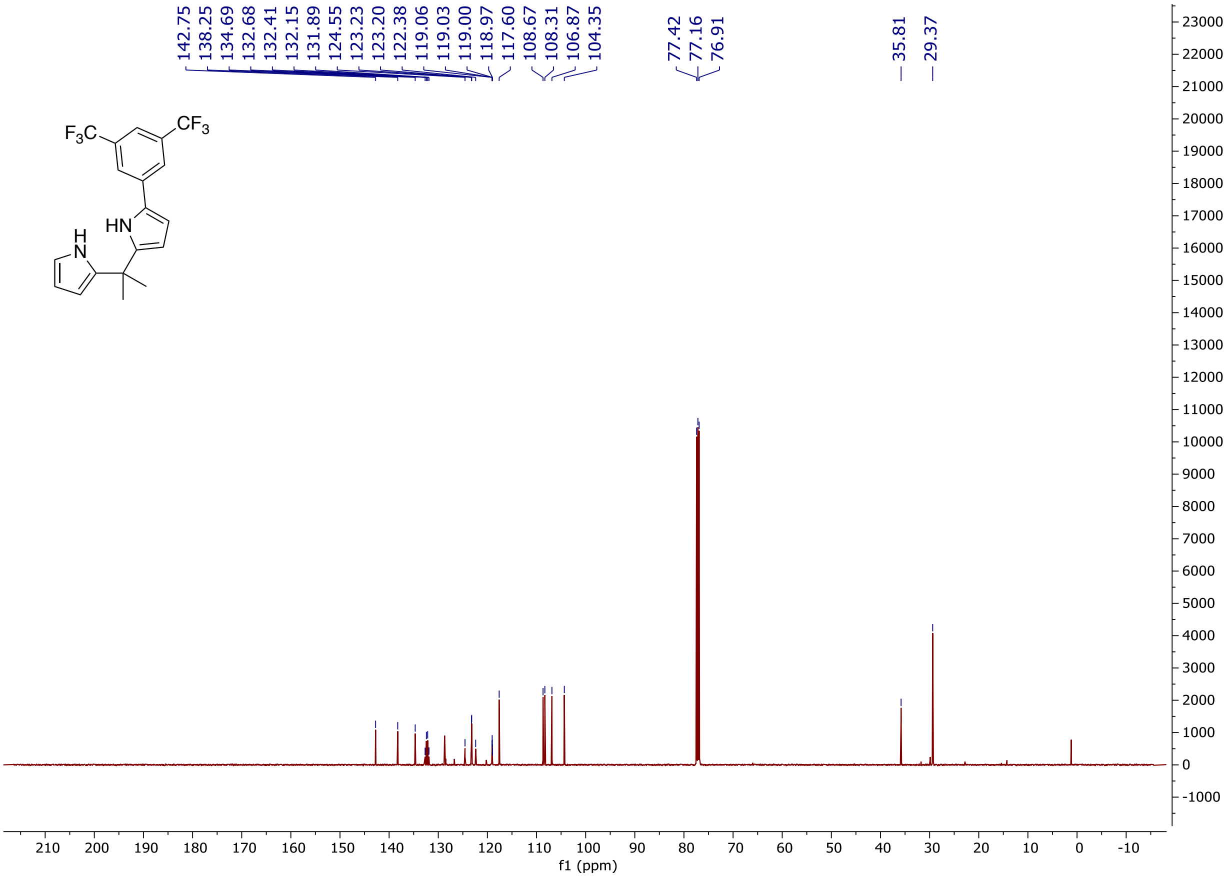

Figure S22. ^{13}*C NMR of* $H_2dpm^{2-(3,5-diCF3Ph)}$

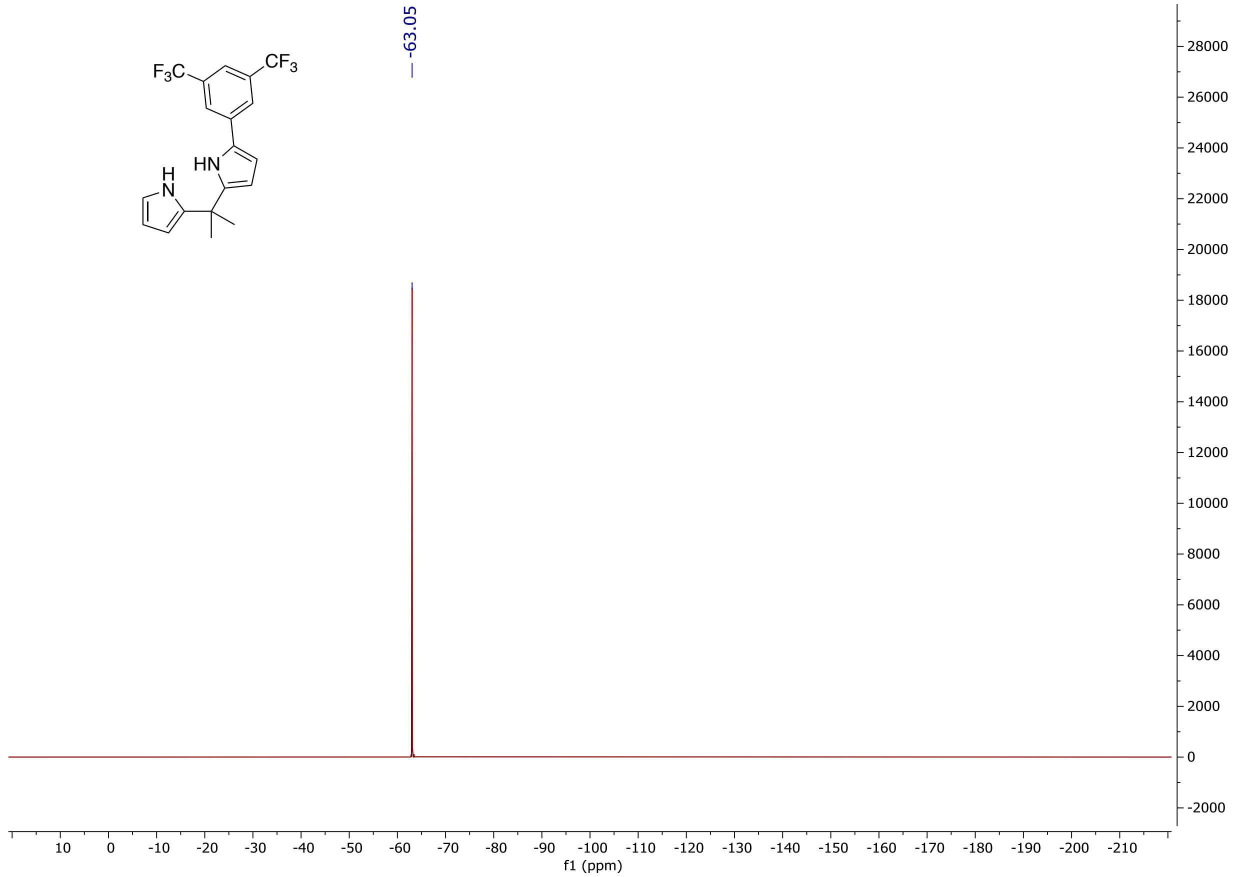

Figure S23. ^{19}*F NMR of* $H_2dpm^{2\text{-}(3,5\text{-}diCF3Ph)}$

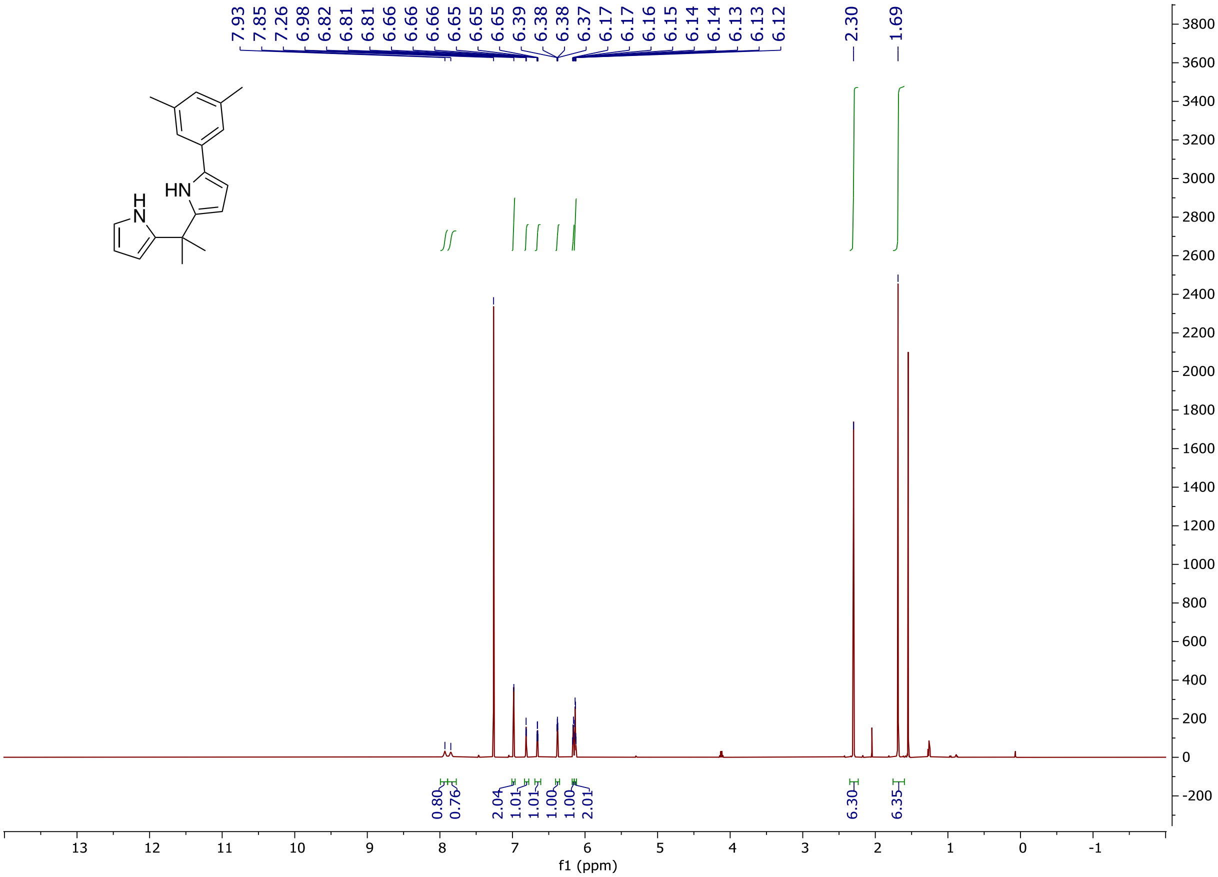

Figure S24. ^{1}H NMR of $H_2dpm^{2\text{-}(3,5\text{-}diMe)}$

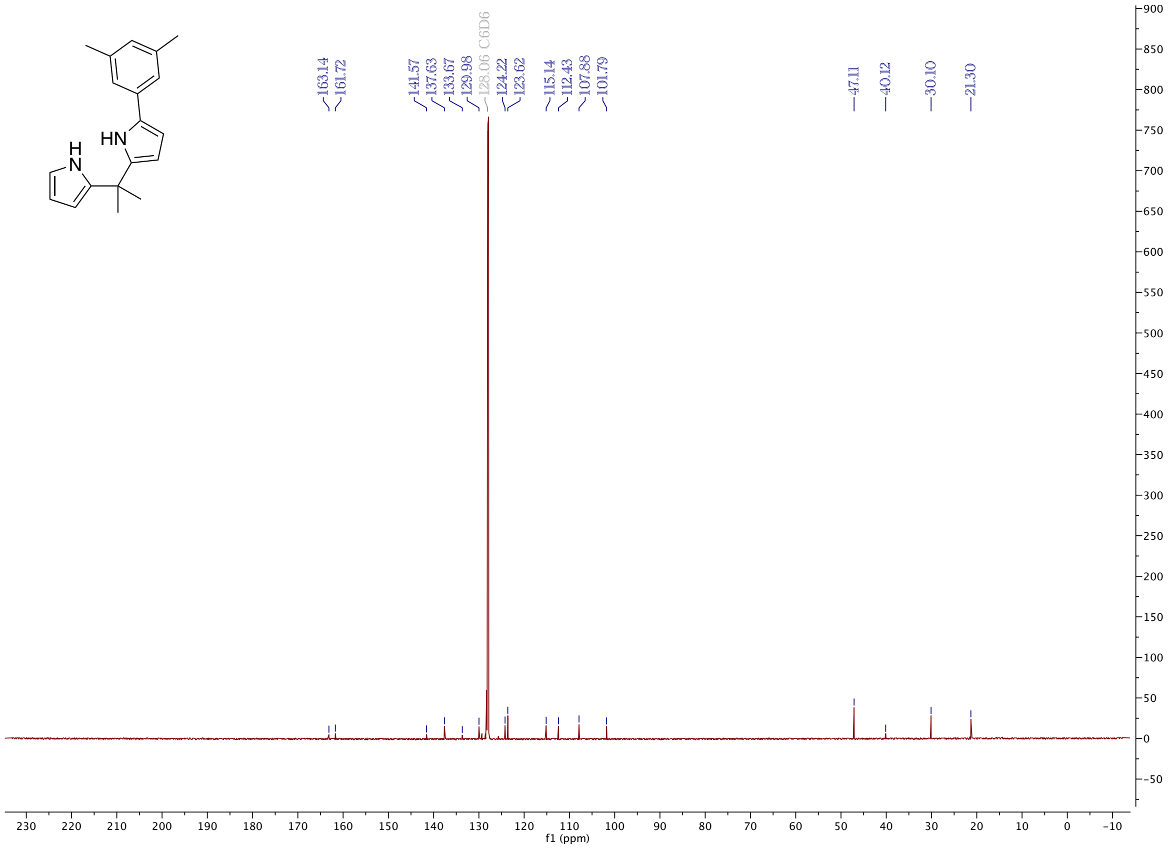

Figure S25. ^{13}C *NMR of* $H_2dpm^{2\text{-}(3,5\text{-}diMe)}$

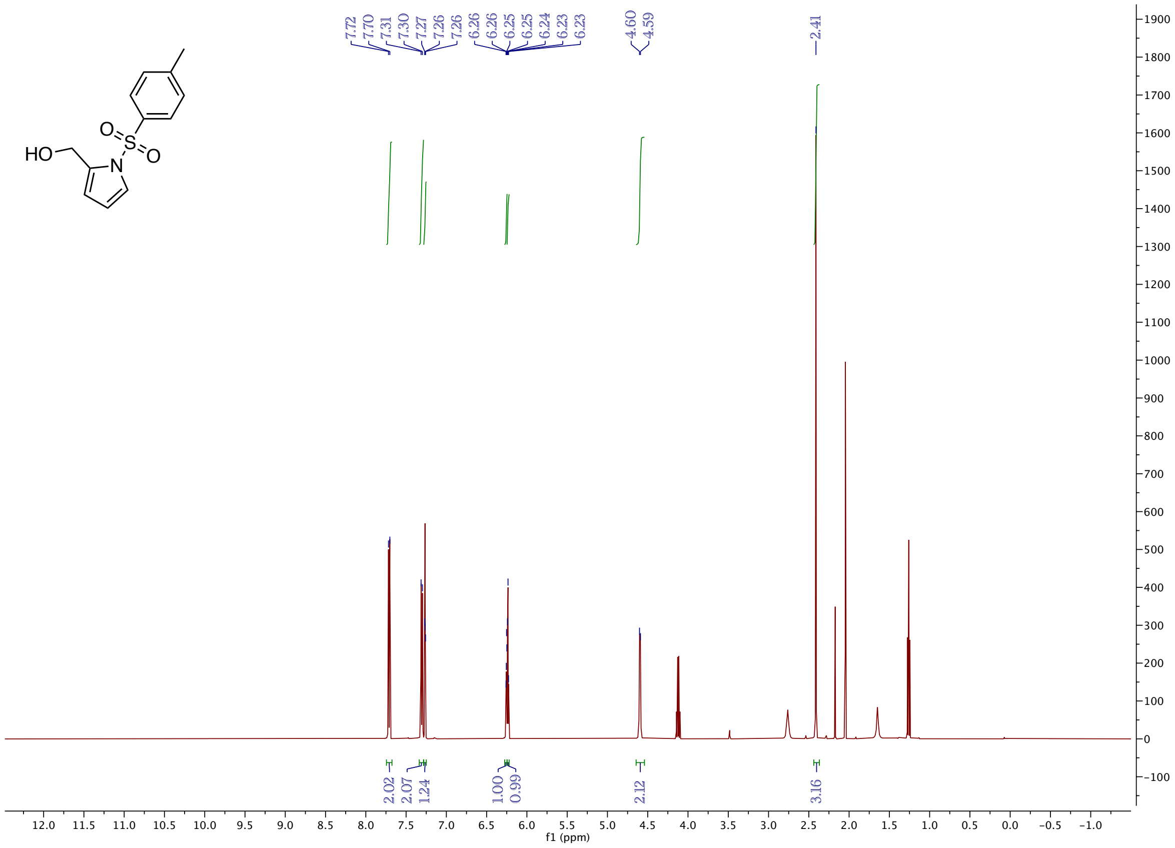

Figure S26. ^{1}H NMR of (1-tosyl-1H-pyrrol-2-yl)methanol

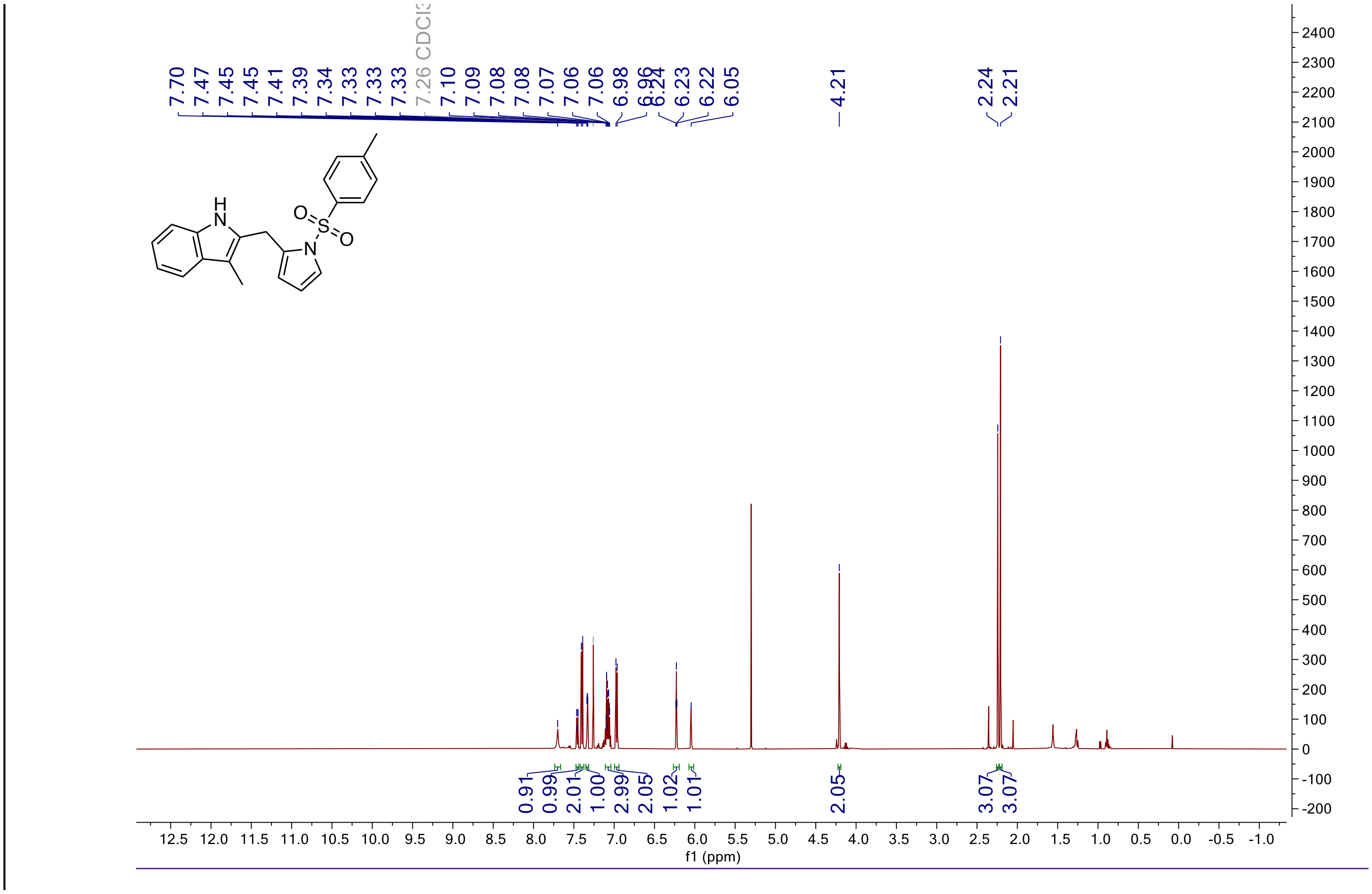

Figure S27. ^{1}H *NMR of 3-methyl-2-((1-tosyl-pyrrol-2-yl)methyl)-1H-indole. Residual solvents peaks (DCM and hexanes) from workup.*

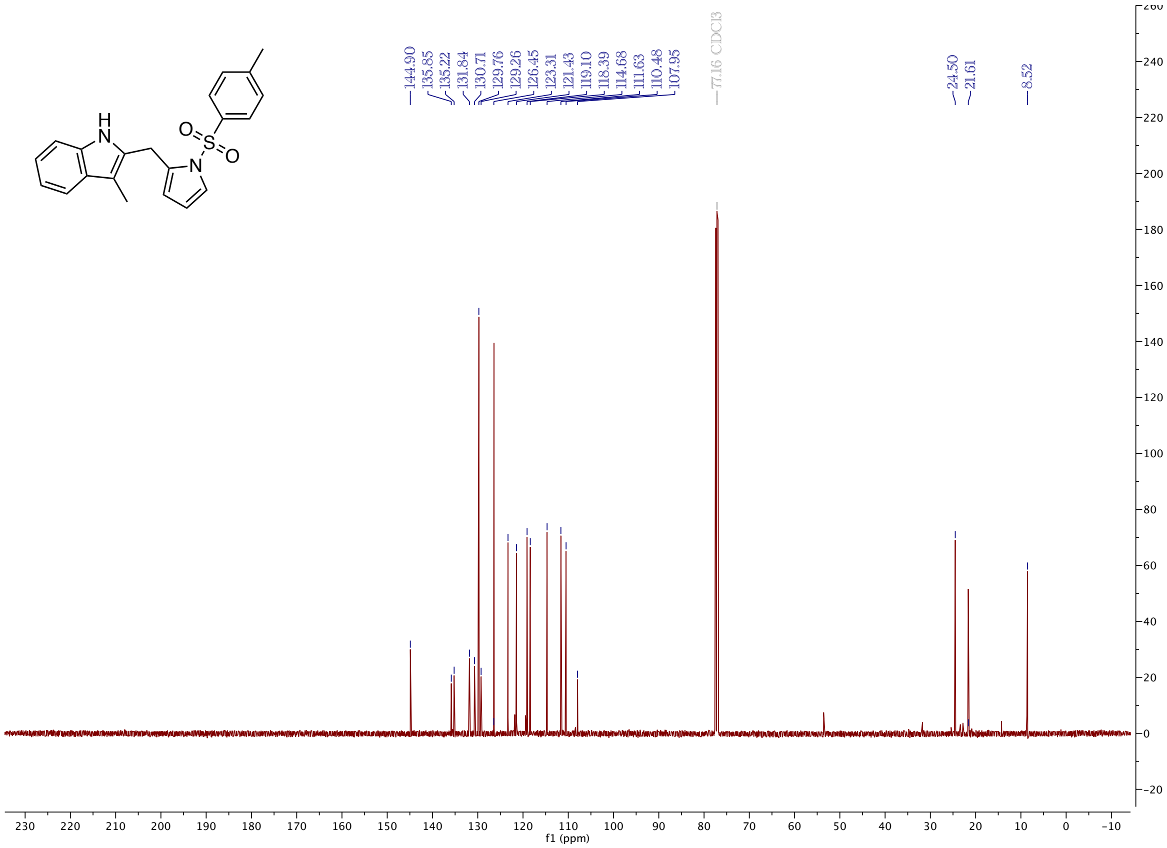

Figure S28. ^{13}*C NMR of 3-methyl-2-((1-tosyl-pyrrol-2-yl)methyl)-1H-indole*

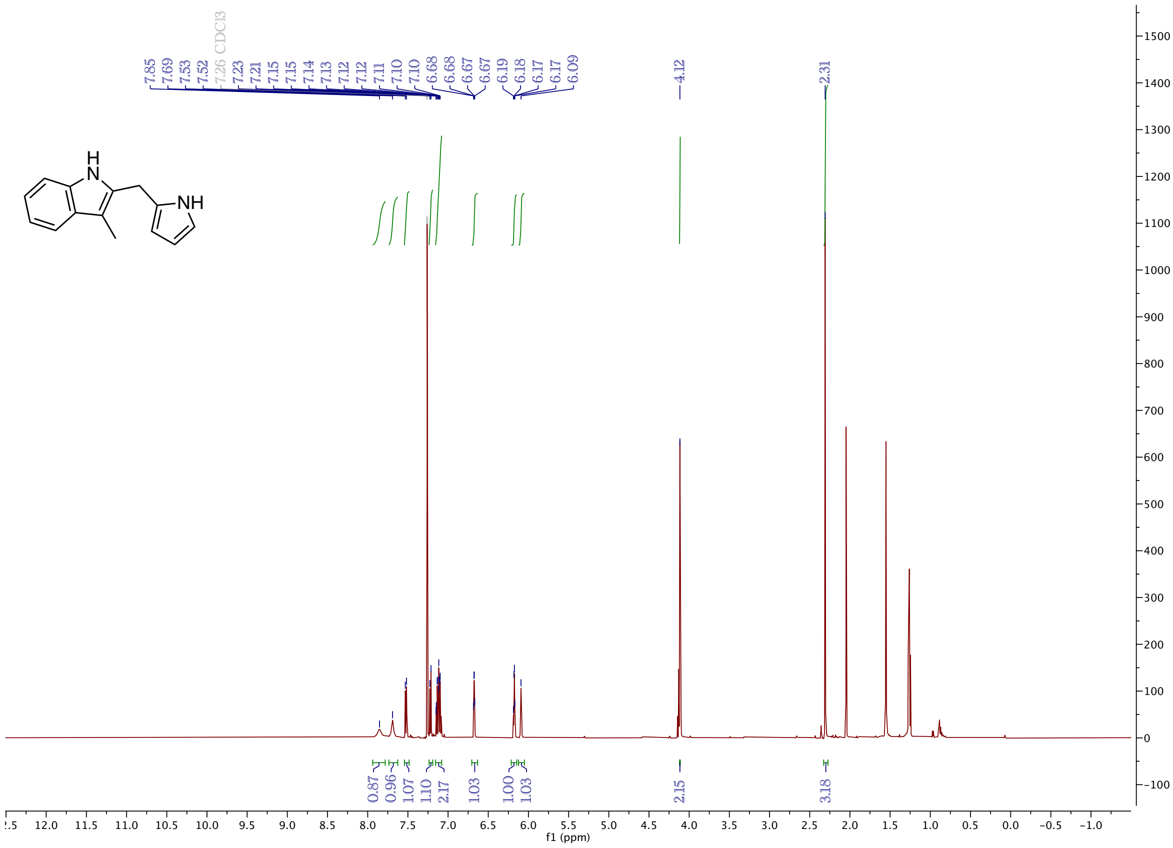

Figure S29. [1]*H NMR of 2-((1H-pyrrol-2-yl)methyl)-3-methylindole. Residual solvent peaks were observed for EtOAc and* H_2O.

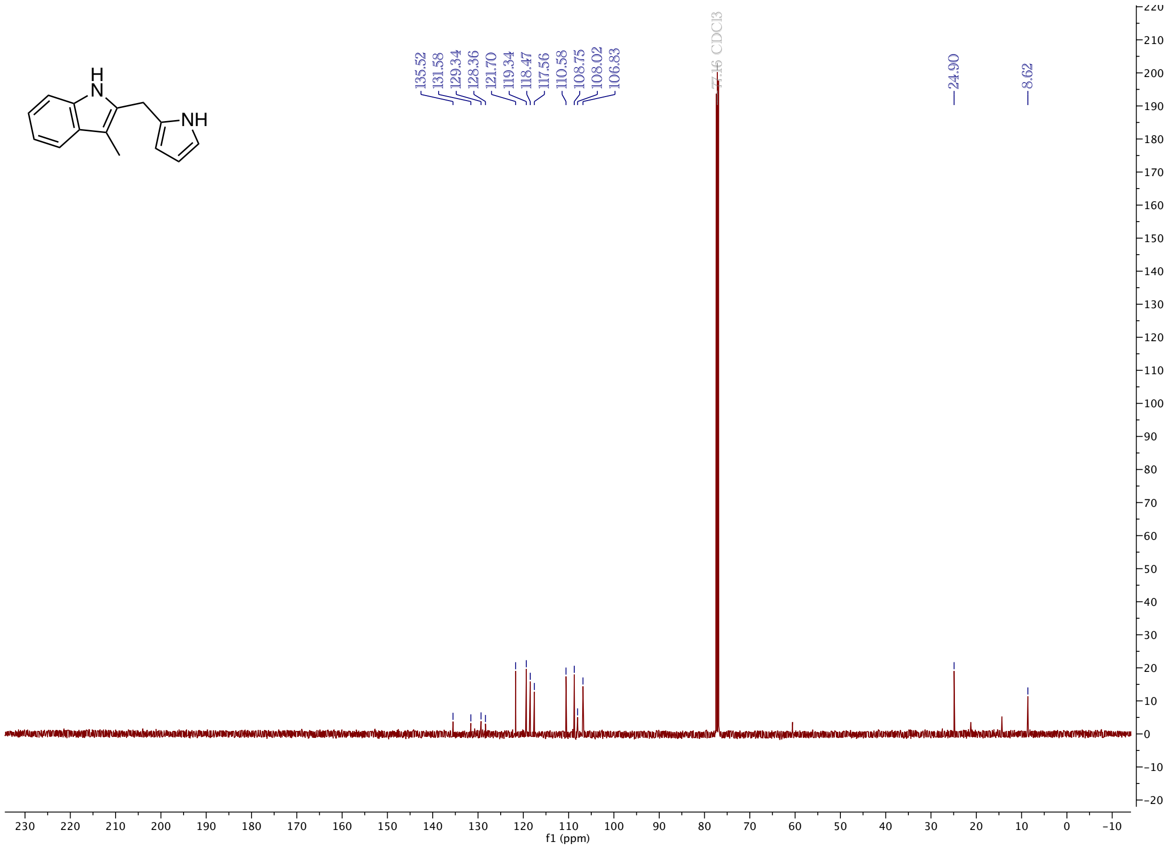

Figure S30. ^{13}C NMR of 2-((1H-pyrrol-2-yl)methyl)-3-methylindole

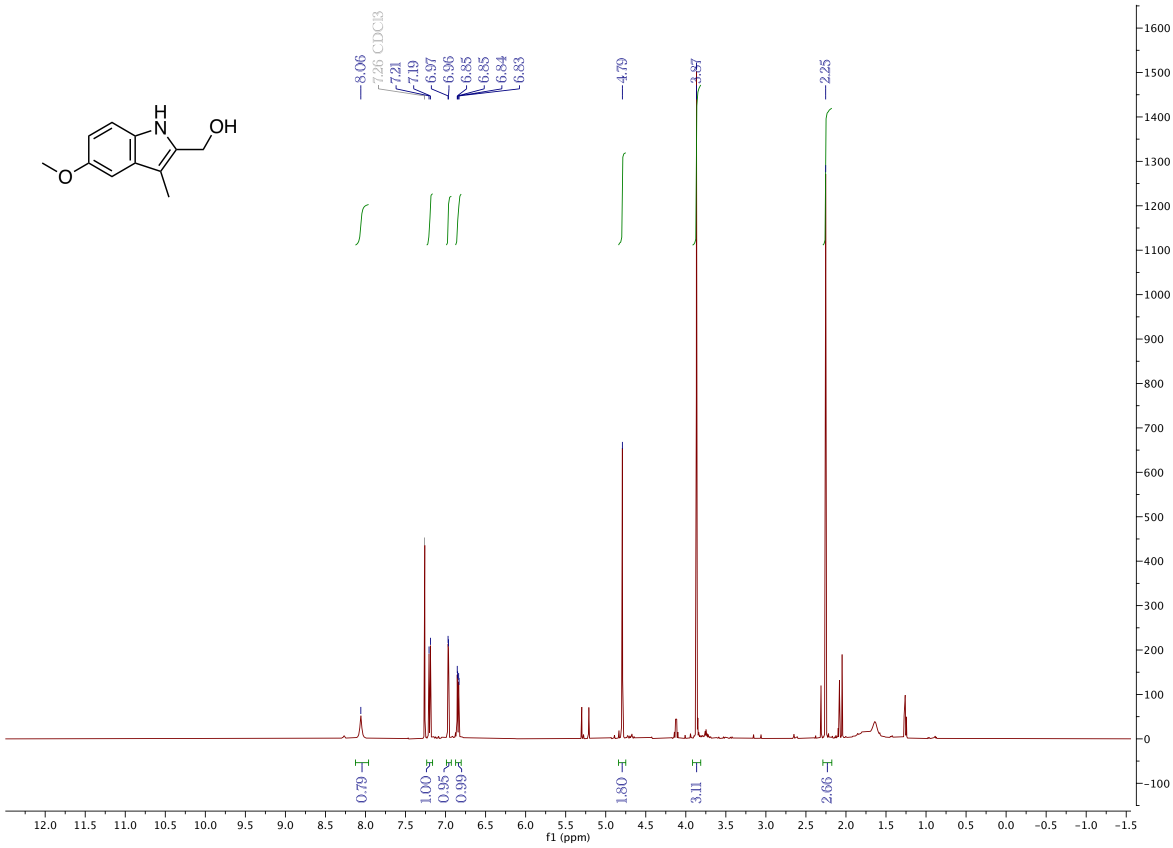

Figure S31. ^{1}H NMR of (5-methoxy-3-methyl-1H-indol-2-yl)methanol

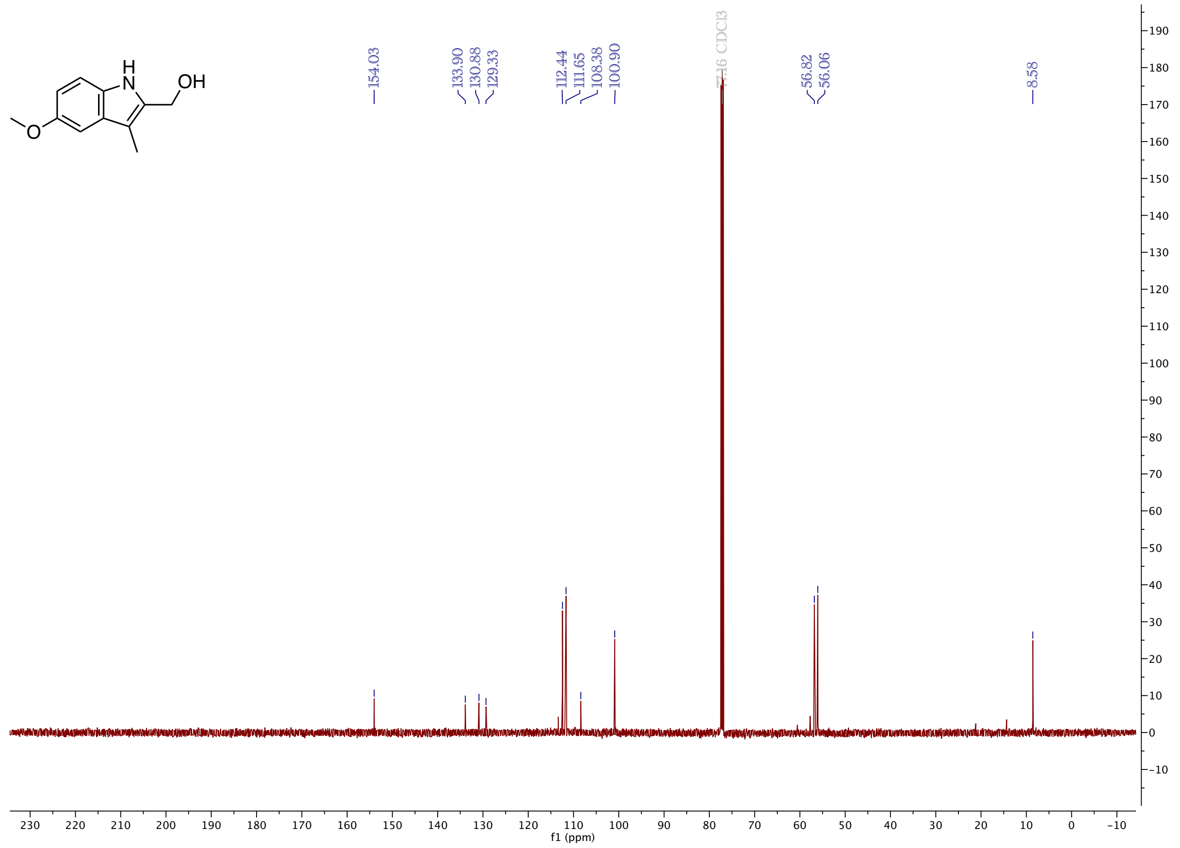

Figure S32. ^{13}C NMR of (5-methoxy-3-methyl-1H-indol-2-yl)methanol

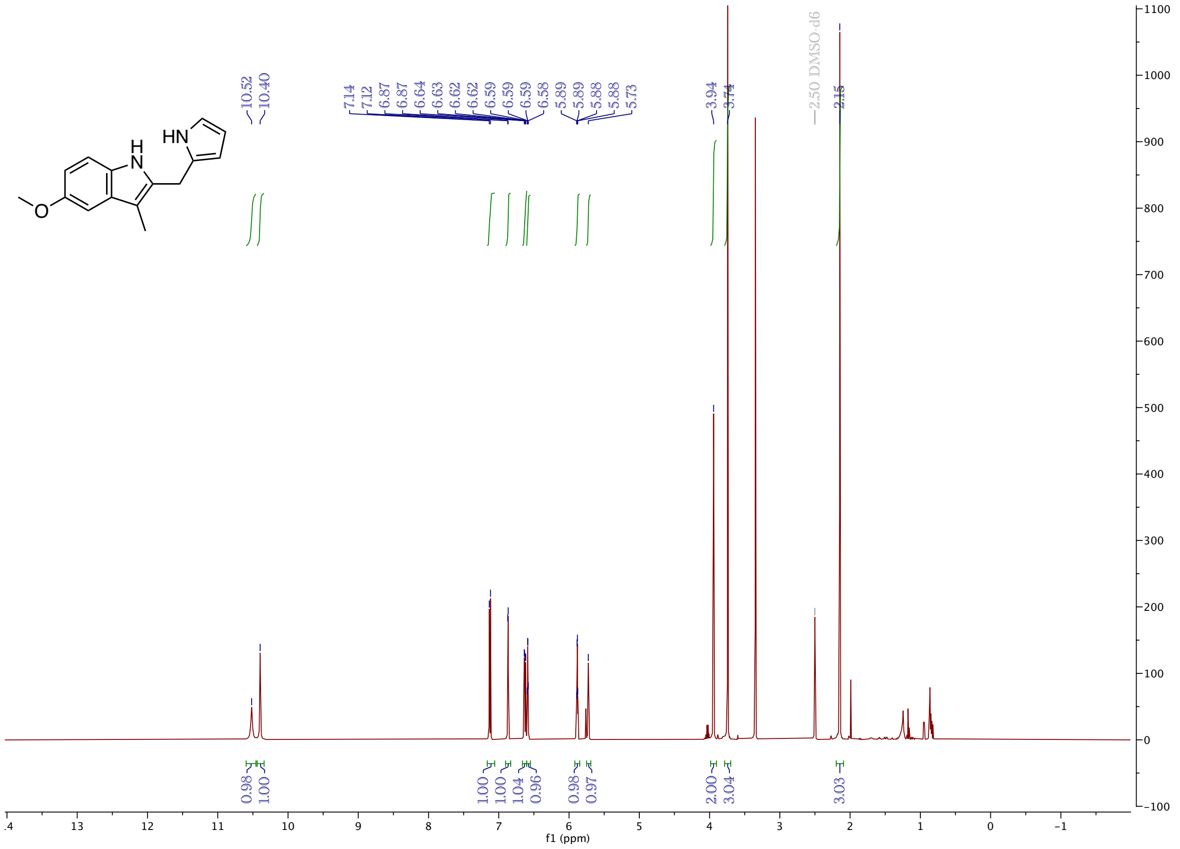

Figure S33. ^{1}H NMR of 2-((1H-pyrrol-2-yl)methyl)-5-methoxy-3-methyl-1H-indole. Residual solvent peaks were observed for hexane and H_2O.

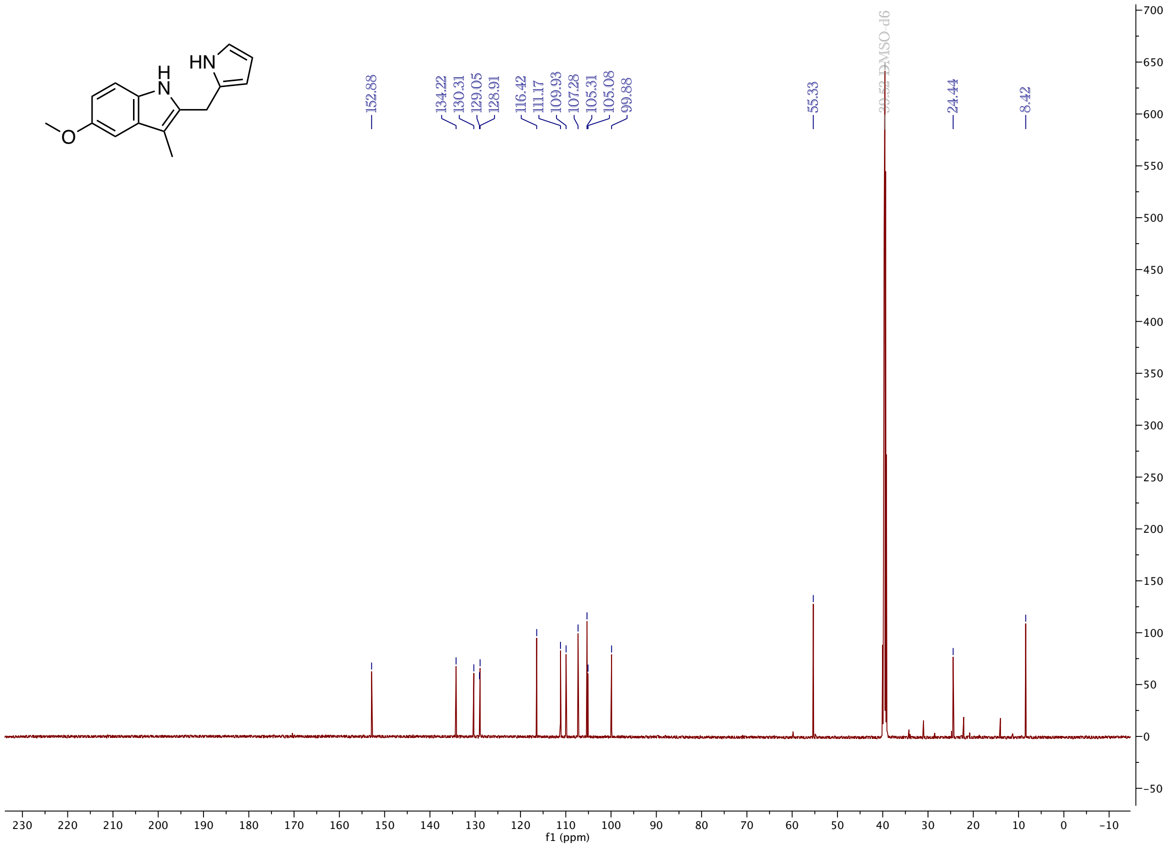

Figure S34. ^{13}C NMR of 2-((1H-pyrrol-2-yl)methyl)-5-methoxy-3-methyl-1H-indole

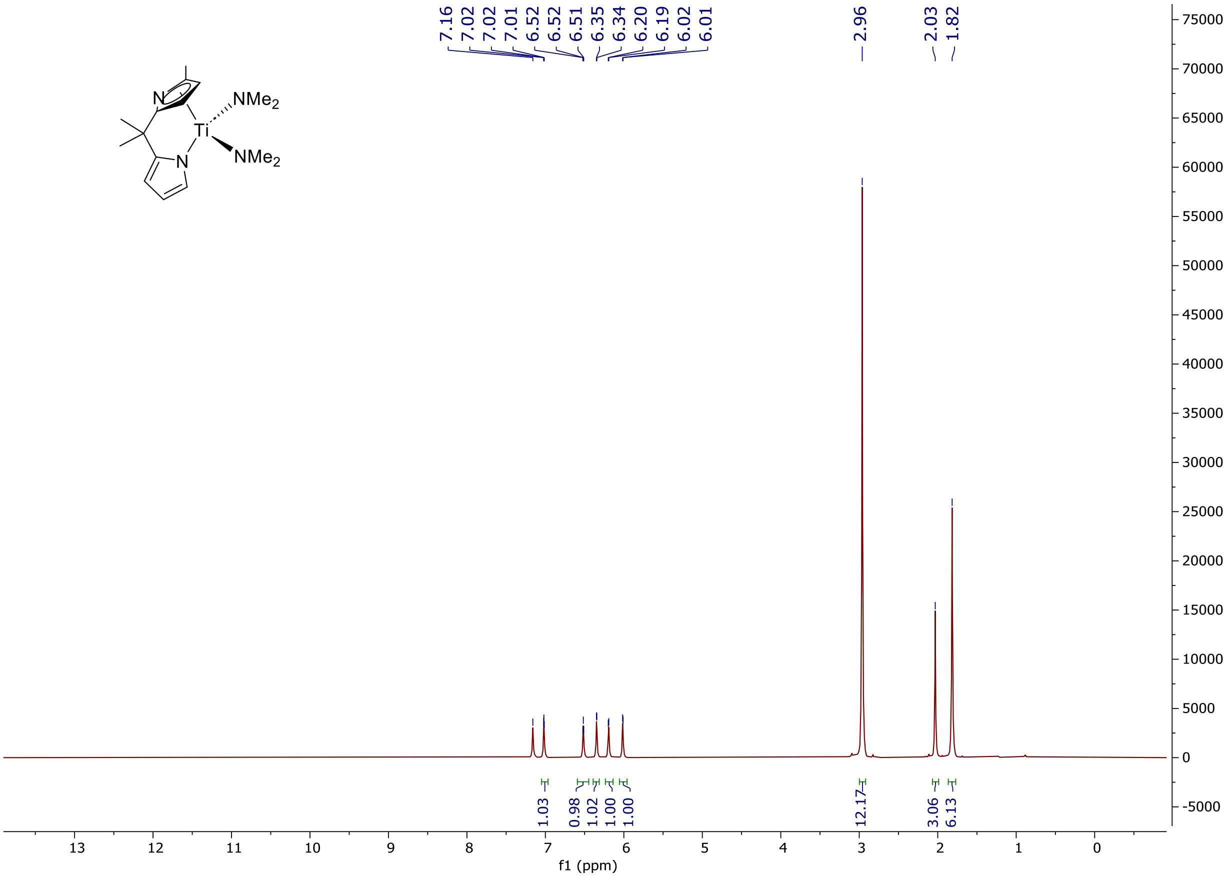

Figure S35. *^{1}H NMR of Ti(dpm$^{2\text{-}Me}$)(NMe$_2$)$_2$* ***(5a)***

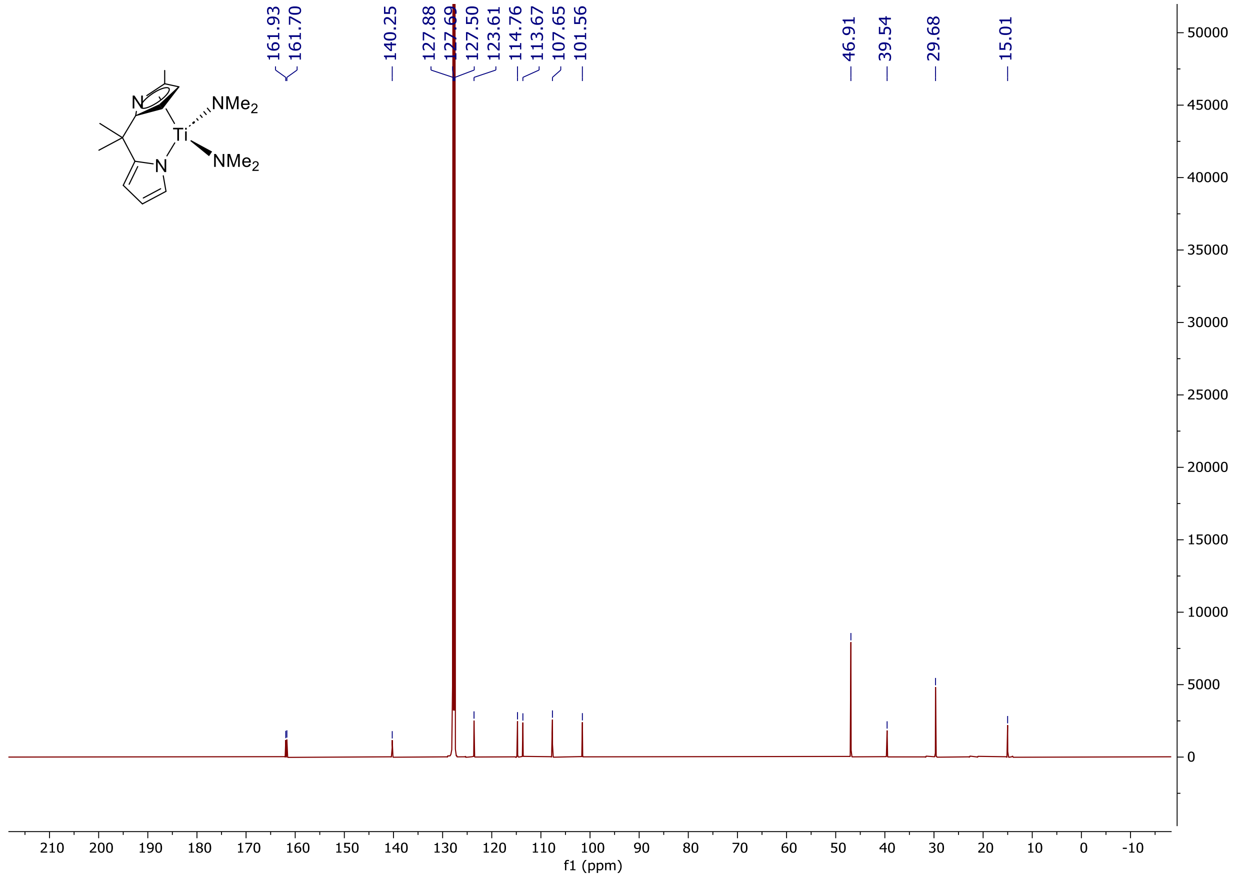

*Figure S36. ^{13}C NMR of Ti(dpm$^{2\text{-}Me}$)(NMe$_2$)$_2$ (**5a**)*

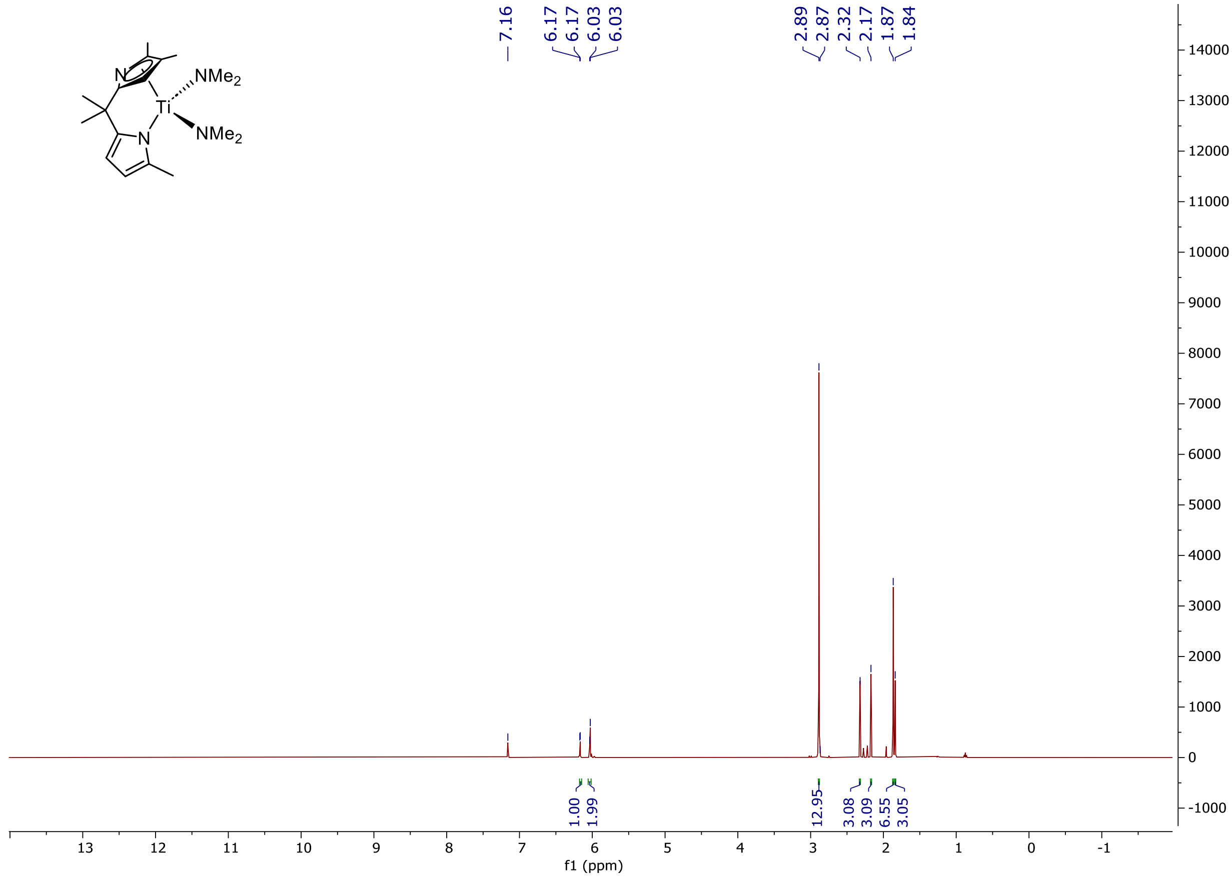

*Figure S37. ^{1}H NMR of Ti(dpm$^{2,2',3\text{-TriMe}}$)(NMe_2)$_2$ (**5b**)*

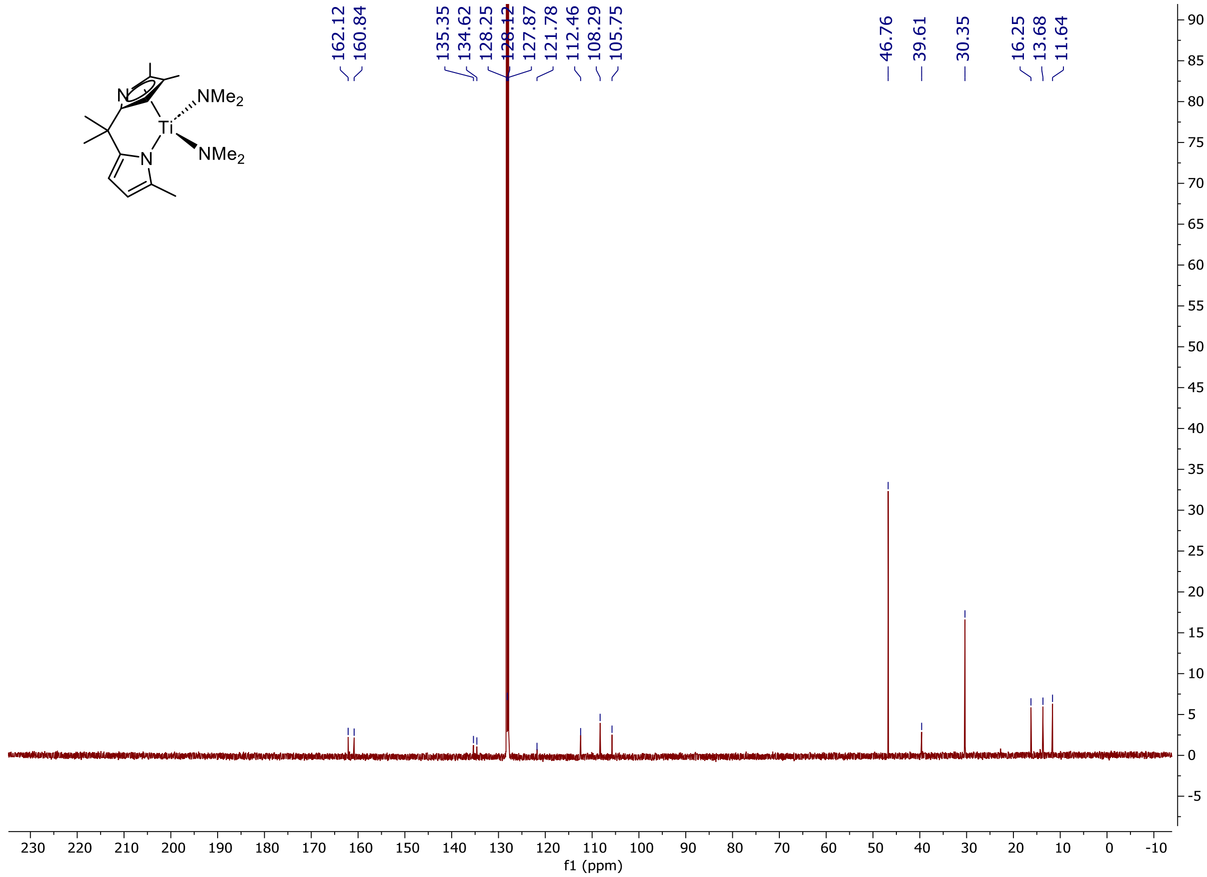

*Figure S38. ^{13}C NMR of Ti(dpm$^{2,2',3\text{-}TriMe}$)(NMe$_2$)$_2$ (**5b**)*

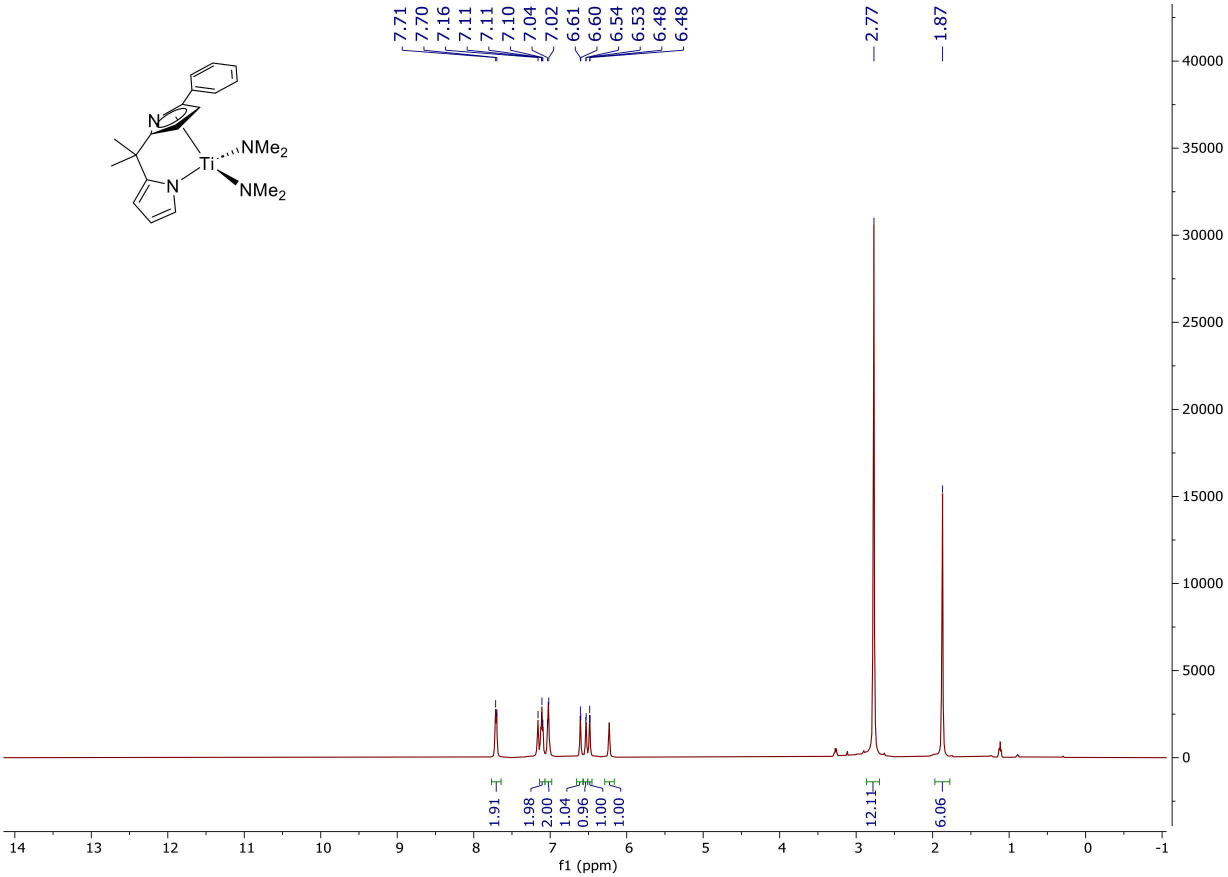

*Figure S39. ^{1}H NMR of Ti(dpm$^{2\text{-}Ph}$)(NMe$_2$)$_2$ (**5c**)*

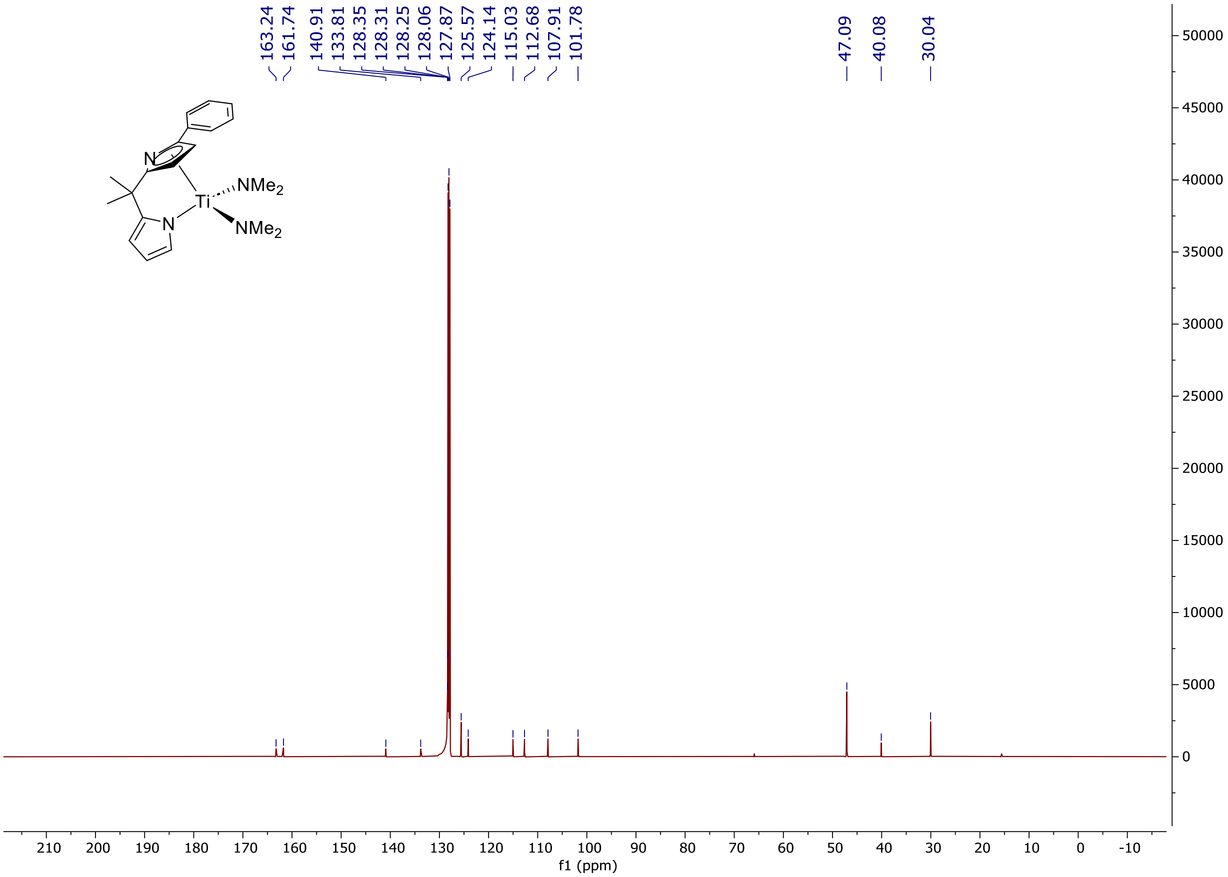

Figure S40. ^{13}*C NMR of Ti(dpm*$^{2\text{-}Ph}$*)(NMe*$_2$*)*$_2$ ***(5c)***

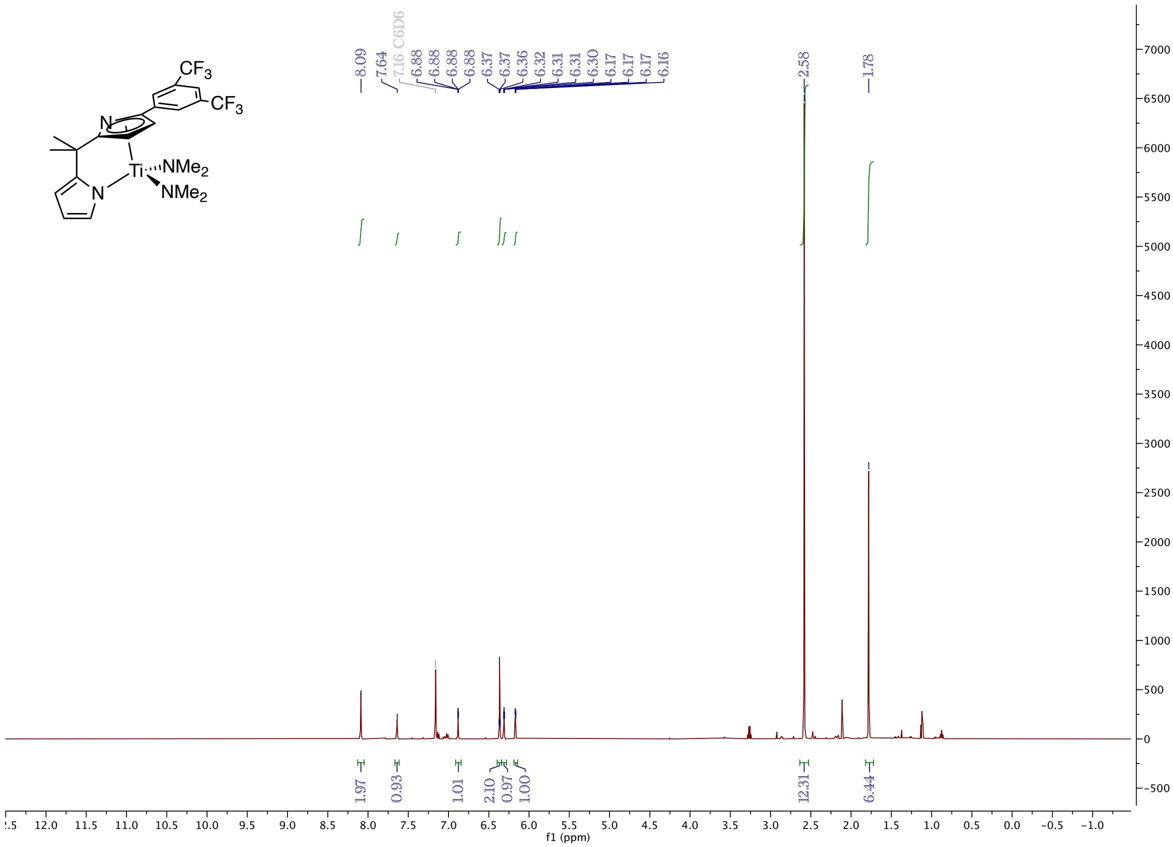

Figure S41. ^{1}H *NMR of* $Ti(pyr^{3,5\text{-}CF3Ph}\text{-}C(CH_3)_2\text{-}pyr)(NMe_2)_2$ (***5d***)

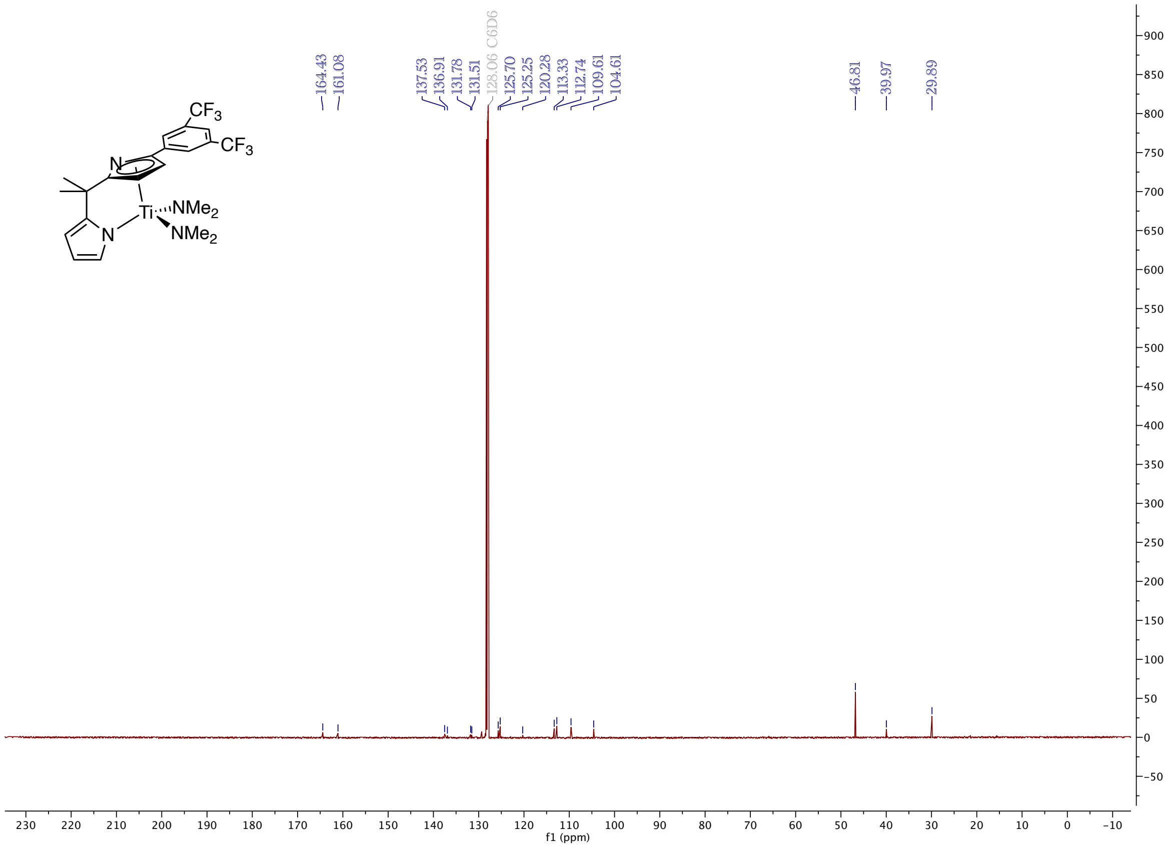

*Figure S42. ^{13}C NMR of Ti(pyr$^{3,5\text{-}CF3Ph}$-C(CH_3)$_2$-pyr)(NMe_2)$_2$ (**5d**)*

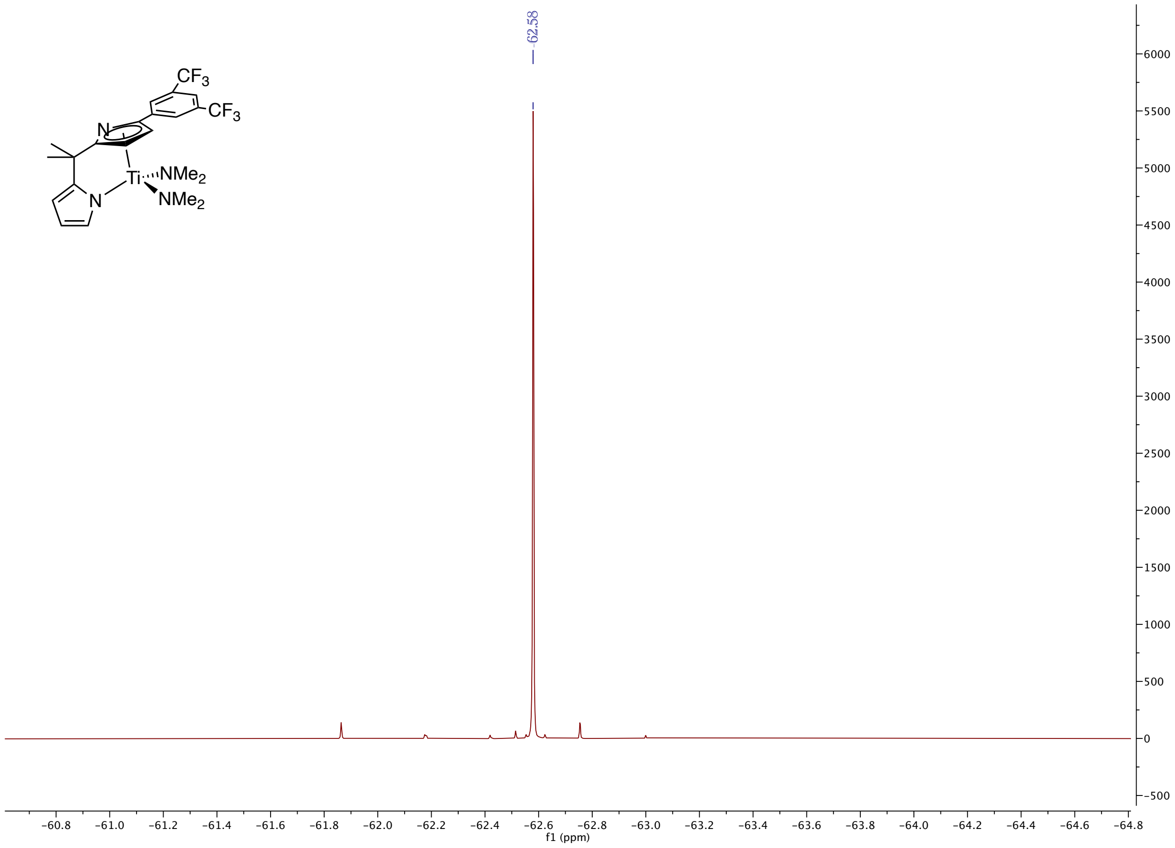

*Figure S43. ^{19}F NMR of Ti(pyr[3,5-CF3Ph]-C(CH3)2-pyr)(NMe2)2 (**5d**)*

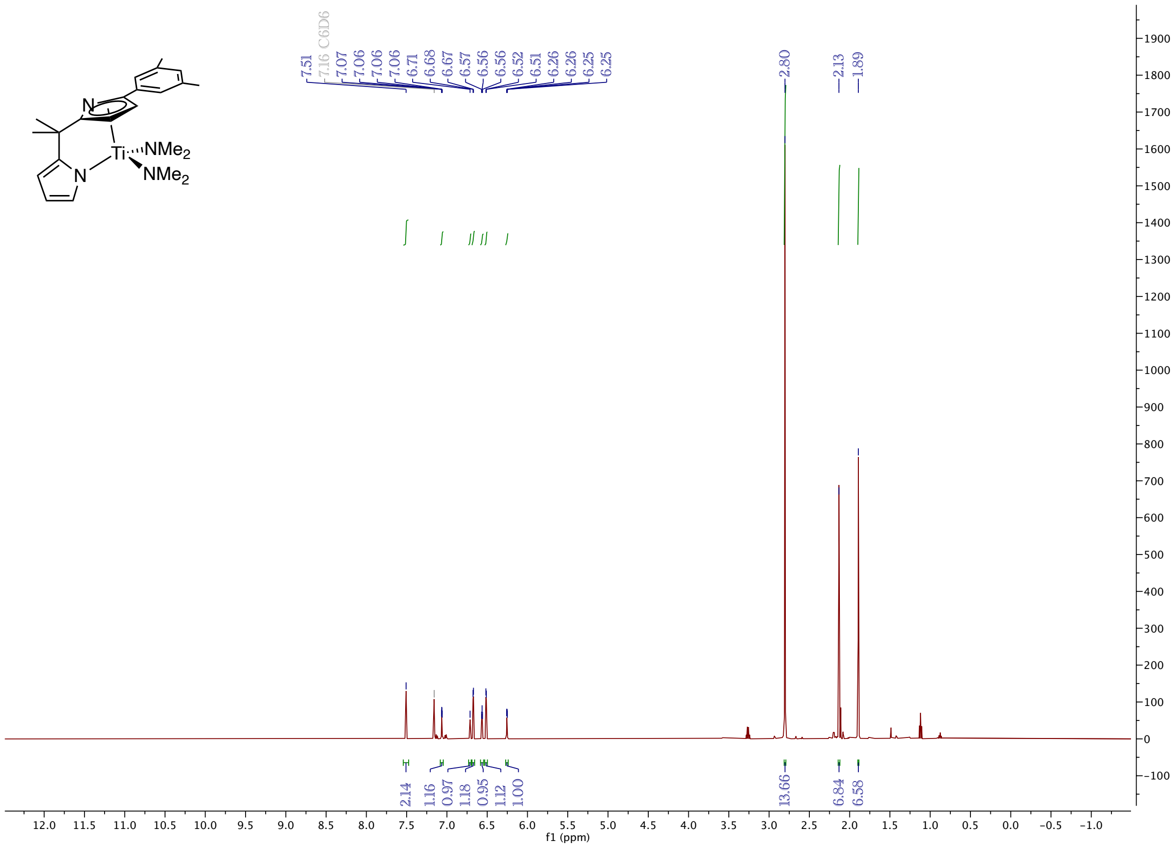

*Figure S44. ^{1}H NMR of Ti(pyr$^{3,5\text{-}diMePh}$-C(CH$_3$)$_2$-pyr)(NMe$_2$)$_2$ (**5e**)*

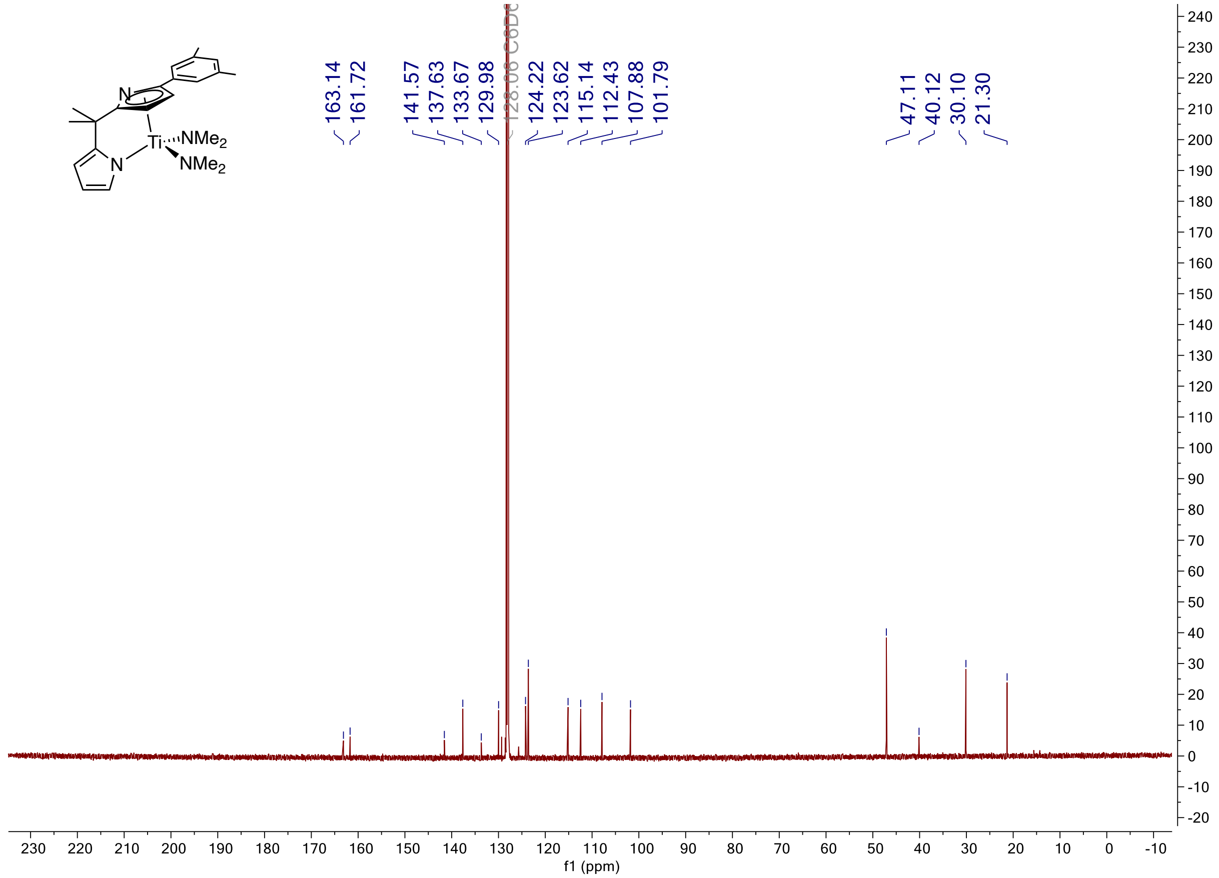

Figure S45. ^{13}*C NMR of Ti(pyr*$^{3,5\text{-}diMePh}$*-C(CH*$_3$*)*$_2$*-pyr)(NMe*$_2$*)*$_2$ *(**5e**)*

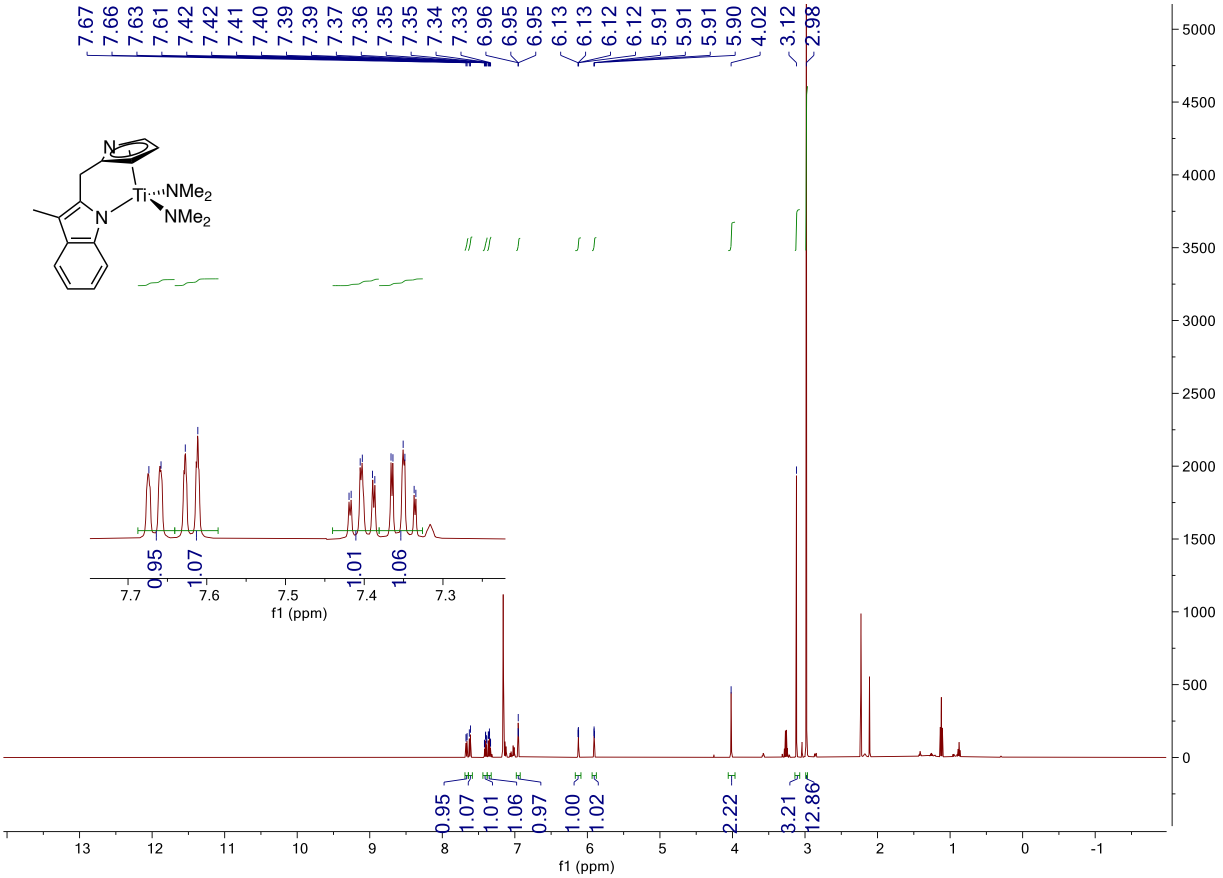

*Figure S46. ^{1}H NMR of Ti(pyr-CH$_2$-ind$^{3\text{-}Me}$)(NMe$_2$)$_2$ (**6a**)*

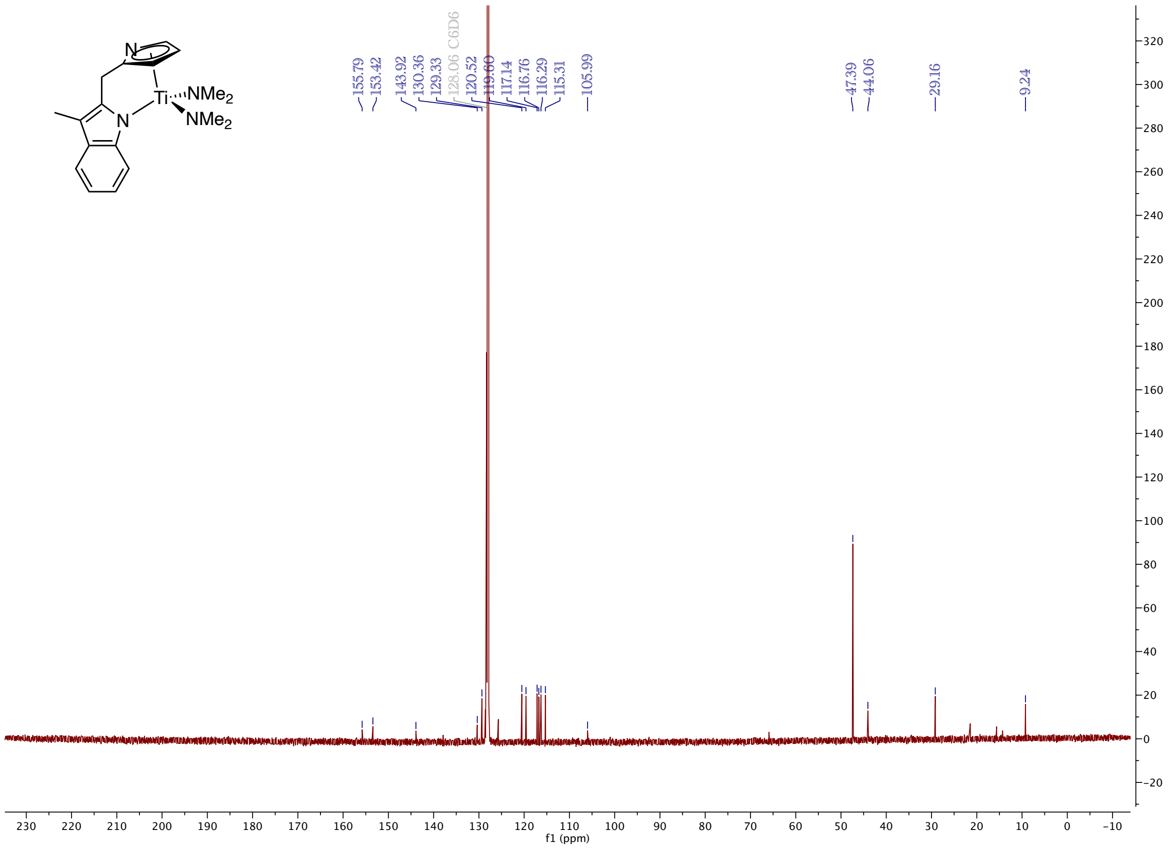

Figure S47. ^{13}*C NMR of Ti(pyr-CH$_2$-ind$^{3\text{-}Me}$)(NMe$_2$)$_2$* ***(6a)***

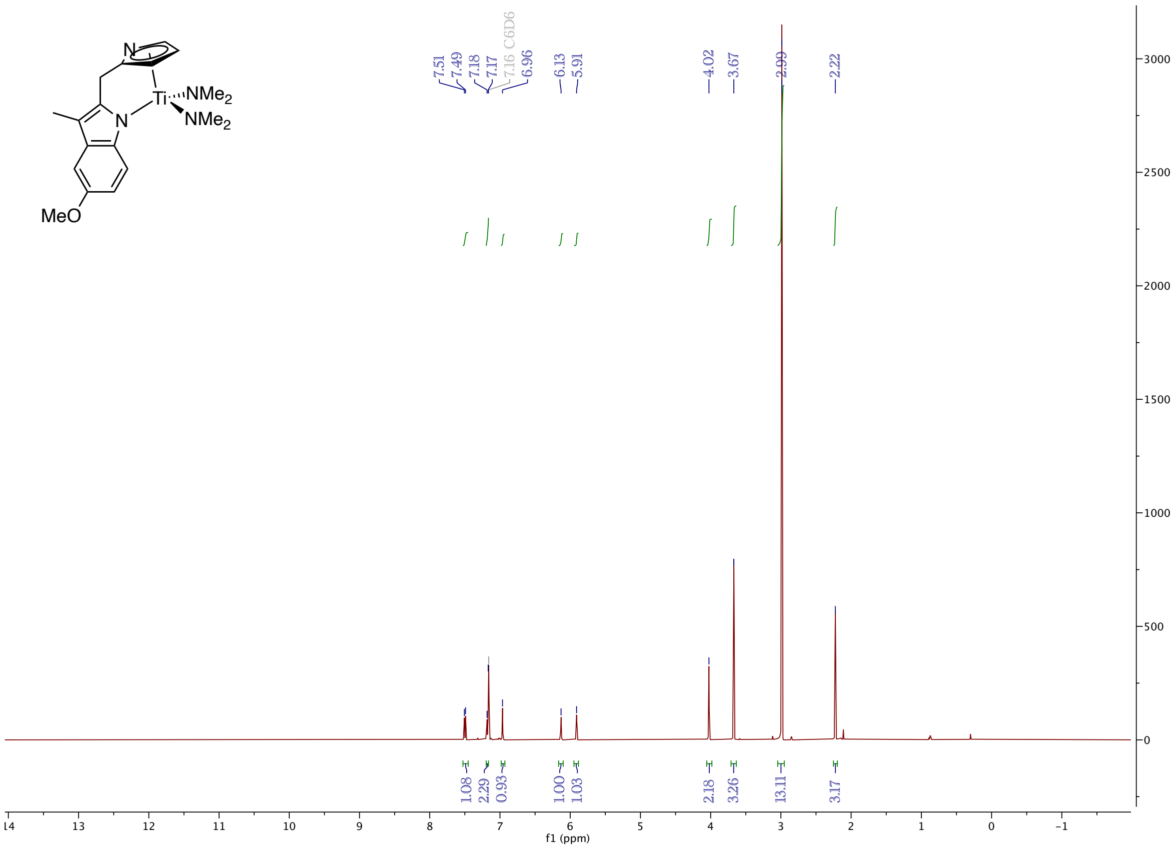

Figure S48. ^{1}H *NMR of Ti(pyr-CH$_2$-ind$^{3\text{-}Me\text{-}5\text{-}OMe}$)(NMe$_2$)$_2$* (***6b***)

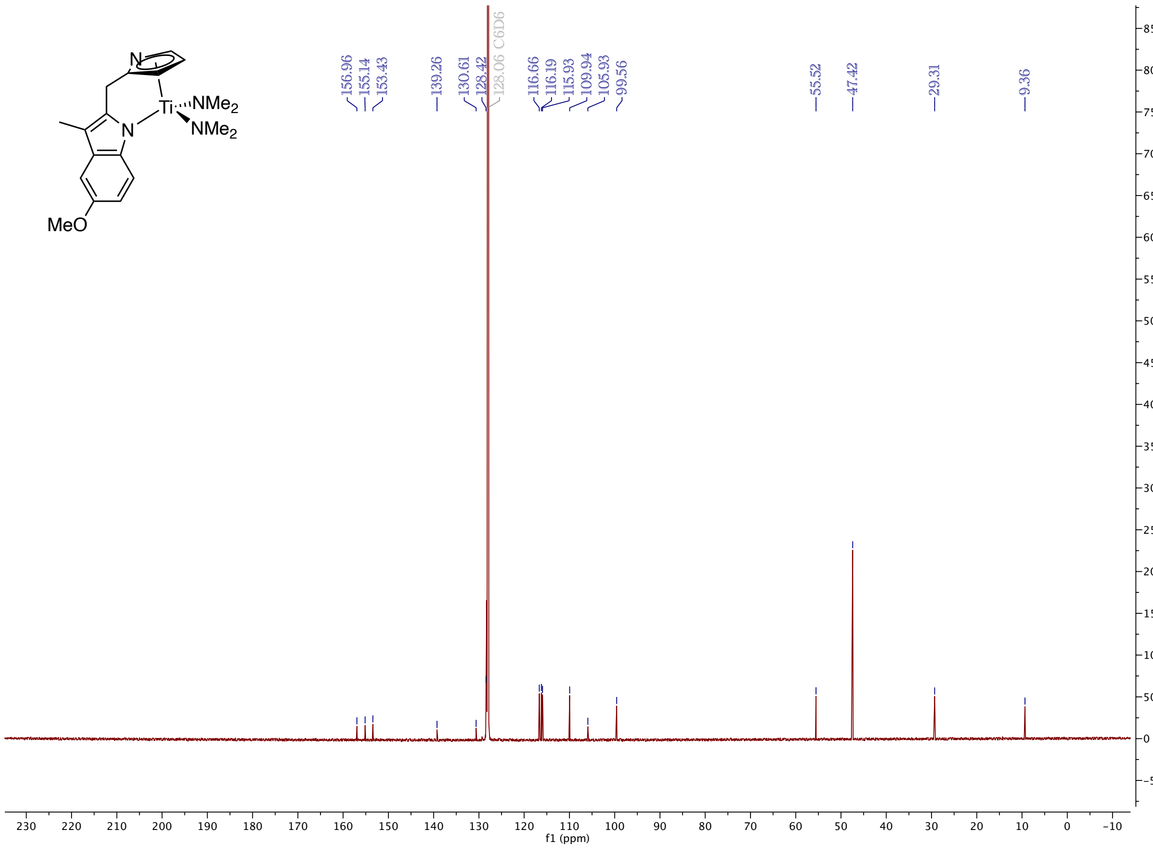

Figure S49. *^{13}C NMR of Ti(pyr-CH$_2$-ind$^{3\text{-}Me\text{-}5\text{-}OMe}$)(NMe$_2$)$_2$* (***6b***)

Single Crystal X-ray Diffraction

Thermal ellipsoids of titanium precatalysts are drawn with 50% probability level. The violet, blue, green, grey, and white spheres represent titanium, nitrogen, fluorine, carbon, and hydrogen atoms, respectively.

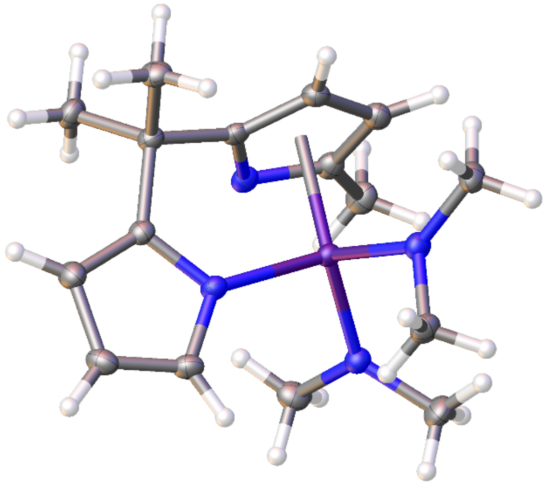

*Figure S50. Structure of Ti(dpm$^{2\text{-}Me}$)(NMe$_2$)$_2$ (**5a**) recrystallized from toluene/n-hexane.*

*Table S3. Crystallographic data and structural refinement of Ti(dpm$^{2\text{-}Me}$)(NMe$_2$)$_2$ (**5a**)*

Empirical formula	$C_{16}H_{26}N_4Ti$
Formula weight	322.31
Temperature/K	100.00(10)
Crystal system	monoclinic
Space group	$P2_1/n$
a/Å	8.09880(10)
b/Å	12.67000(10)
c/Å	16.3955(2)
α/°	90
β/°	98.0760(10)
γ/°	90
Volume/Å^3	1665.69(3)
Z	4
ρ_{calc}g/cm^3	1.285
μ/mm^{-1}	4.334
F(000)	688.0

Crystal size/mm^3	0.188 × 0.143 × 0.105
Radiation	Cu Kα (λ = 1.54184)
2Θ range for data collection/°	8.854 to 160.594
Index ranges	-10 ≤ h ≤ 10, -15 ≤ k ≤ 15, -19 ≤ l ≤ 20
Reflections collected	24926
Independent reflections	3606 [R_{int} = 0.0352, R_{sigma} = 0.0219]
Data/restraints/parameters	3606/0/197
Goodness-of-fit on F^2	1.101
Final R indexes [I>=2σ (I)]	R_1 = 0.0293, wR_2 = 0.0791
Final R indexes [all data]	R_1 = 0.0310, wR_2 = 0.0804
Largest diff. peak/hole / e Å^{-3}	0.38/-0.35

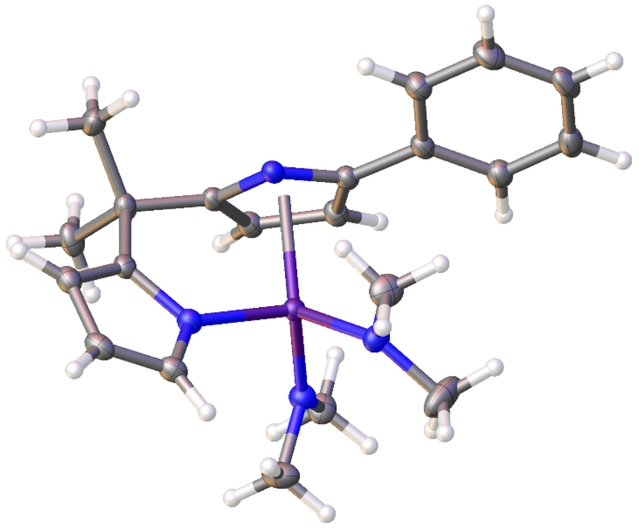

*Figure S51. Structure of Ti(dpm$^{2\text{-}Ph}$)(NMe$_2$)$_2$ (**5c**) recrystallized from toluene/n-hexane.*

*Table S4. Crystallographic data and structural refinement of Ti(dpm$^{2\text{-}Ph}$)(NMe$_2$)$_2$ (**5c**)*

Empirical formula	$C_{21}H_{28}N_4Ti$
Formula weight	384.37
Temperature/K	100.00(10)
Crystal system	monoclinic
Space group	$P2_1/n$
a/Å	10.92810(10)

b/Å	15.12680(10)
c/Å	12.41070(10)
α/°	90
β/°	98.5360(10)
γ/°	90
Volume/$Å^3$	2028.85(3)
Z	4
$\rho_{calc}g/cm^3$	1.258
μ/mm^{-1}	3.647
F(000)	816.0
Crystal size/mm^3	0.123 × 0.091 × 0.069
Radiation	Cu Kα (λ = 1.54184)
2Θ range for data collection/°	9.28 to 160.75
Index ranges	$-13 \le h \le 10, -19 \le k \le 19, -15 \le l \le 15$
Reflections collected	31126
Independent reflections	4401 [R_{int} = 0.0355, R_{sigma} = 0.0235]
Data/restraints/parameters	4401/0/241
Goodness-of-fit on F^2	1.103
Final R indexes [I>=2σ (I)]	R_1 = 0.0306, wR_2 = 0.0858
Final R indexes [all data]	R_1 = 0.0323, wR_2 = 0.0869
Largest diff. peak/hole / e $Å^{-3}$	0.31/-0.38

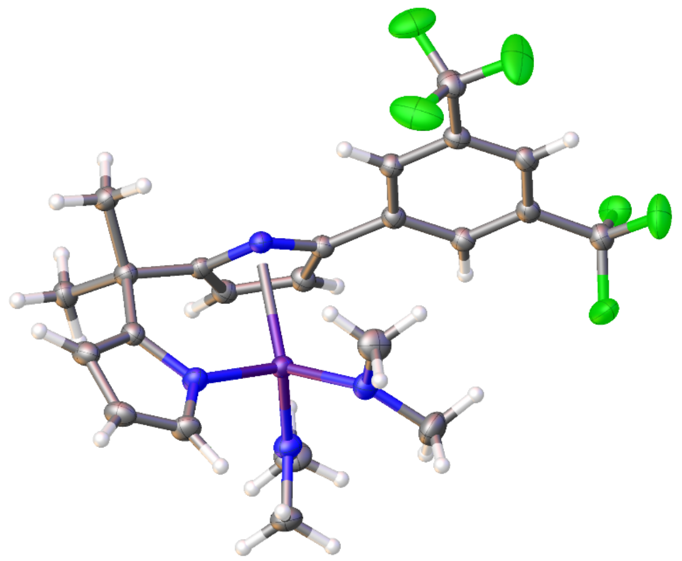

Figure S52. Structure of Ti(pyr$^{3,5\text{-}CF3Ph}$-C(CH$_3$)$_2$-pyr)(NMe$_2$)$_2$ (5d) recrystallized from ether/n-hexane.

*Table S5. Crystallographic data and structural refinement of Ti(pyr$^{3,5\text{-}CF3Ph}$-C(CH$_3$)$_2$-pyr)(NMe$_2$)$_2$ (**5d**)*

Empirical formula	$C_{46}H_{52}F_{12}N_8Ti_2$
Formula weight	1040.75
Temperature/K	99.99(10)
Crystal system	monoclinic
Space group	$P2_1/n$
a/Å	15.8889(2)
b/Å	8.43720(10)
c/Å	35.4820(4)
α/°	90
β/°	94.2020(10)
γ/°	90
Volume/Å^3	4743.85(10)
Z	4
ρ_{calc}g/cm^3	1.457
μ/mm^{-1}	3.654
F(000)	2144.0
Crystal size/mm^3	0.133 × 0.12 × 0.077
Radiation	Cu Kα (λ = 1.54184)

2Θ range for data collection/°	4.994 to 159.652
Index ranges	$-20 \leq h \leq 16$, $-10 \leq k \leq 9$, $-42 \leq l \leq 45$
Reflections collected	38974
Independent reflections	10024 [R_{int} = 0.0355, R_{sigma} = 0.0335]
Data/restraints/parameters	10024/0/625
Goodness-of-fit on F^2	1.052
Final R indexes [I>=2σ (I)]	R_1 = 0.0366, wR_2 = 0.0929
Final R indexes [all data]	R_1 = 0.0409, wR_2 = 0.0956
Largest diff. peak/hole / e $Å^{-3}$	0.52/-0.61

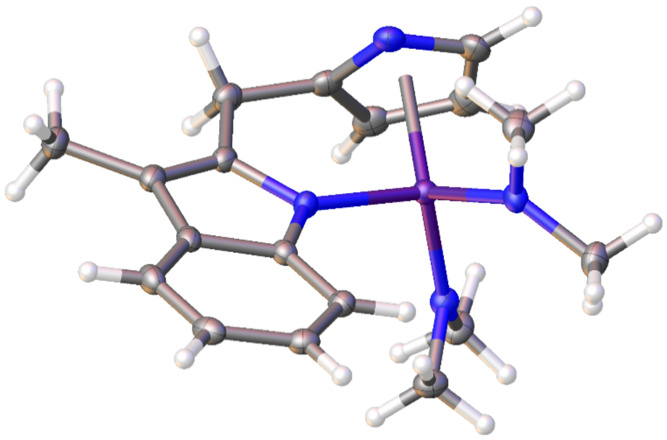

*Figure S53. Structure of Ti(pyr-CH$_2$-ind$^{3\text{-}Me}$)(NMe$_2$)$_2$ (**6a**) recrystallized from ether/n-hexane.*

*Table S6. Crystallographic data and structural refinement of Ti(pyr-CH$_2$-ind$^{3\text{-}Me}$)(NMe$_2$)$_2$ (**6a**)*

Empirical formula	$C_{18}H_{24}N_4Ti$
Formula weight	344.31
Temperature/K	100.00(10)
Crystal system	monoclinic
Space group	$P2_1/n$
a/Å	8.63010(10)
b/Å	19.5622(3)
c/Å	10.6847(2)
α/°	90
β/°	108.138(2)

γ/°	90
Volume/Å^3	1714.20(5)
Z	4
ρ_{calc}g/cm^3	1.334
μ/mm^{-1}	4.253
F(000)	728.0
Crystal size/mm^3	0.184 × 0.101 × 0.091
Radiation	Cu Kα (λ = 1.54184)
2Θ range for data collection/°	9.042 to 159.724
Index ranges	$-5 \leq h \leq 10$, $-24 \leq k \leq 24$, $-13 \leq l \leq 13$
Reflections collected	15019
Independent reflections	3650 [R_{int} = 0.0382, R_{sigma} = 0.0321]
Data/restraints/parameters	3650/0/213
Goodness-of-fit on F^2	1.088
Final R indexes [I>=2σ (I)]	R_1 = 0.0323, wR_2 = 0.0835
Final R indexes [all data]	R_1 = 0.0343, wR_2 = 0.0847
Largest diff. peak/hole / e Å^{-3}	0.31/-0.32

References

1. Hickey, A.; Merz, J.; Al Mamari, H. H.; Friedrich, A.; Marder, T. B.; McGlacken, G. P., Iridium-Catalyzed Borylation of 6-Fluoroquinolines: Access to 6-Fluoroquinolones *J. Org. Chem.* **2022,** *87*, 9977-9987.

2. Eerdun, C.; Hisanaga, S.; Setsune, J., Single Helicates of Dipalladium(II) Hexapyrroles: Helicity Induction and Redox Tuning of Chiroptical Properties *Angew. Chem. Int. Ed.* **2013,** *52*, 929-932.

3. Kotthaus, A. F.; Ballaschk, F.; Stakaj, V.; Mohr, F.; Kirsch, S. F., Synthesis and Resolution of a Chiral Diamine: 2,2′-(Propane-2,2-diyl)dipyrrolidine *Synthesis-Stuttgart* **2017,** *49*, 3107-3111.

4. Dolomanov, O. V.; Bourhis, L. J.; Gildea, R. J.; Howard, J. A. K.; Puschmann, H., *OLEX2*: A complete structure solution, refinement and analysis program *J. Appl. Crystallogr.* **2009,** *42*, 339-341.

5. Loughlin, W. A.; Jenkins, I. D.; Karis, N. D.; Schweiker, S. S.; Healy, P. C., 2-Oxo-1,2-dihydropyridinyl-3-yl amide-based GPa inhibitors: Design, synthesis and structure-activity relationship study *Eur. J. Med. Chem.* **2016,** *111*, 1-14.

6. Loughlin, W. A.; Jenkins, I. D.; Karis, N. D.; Schweiker, S. S.; Healy, P. C., 2-Oxo-1,2-dihydropyridinyl-3-yl amide-based GPa inhibitors: Design, synthesis and structure-activity relationship study *European Journal of Medicinal Chemistry* **2016,** *111*, 1-14.

7. Billow, B. S.; McDaniel, T. J.; Odom, A. L., Quantifying ligand effects in high-oxidation-state metal catalysis *Nature Chem.* **2017,** *9*, 837-842.

8. Espenson, J. H., *Chemical Kinetics and Reaction Mechanisms* 2nd ed.; McGraw-Hill: New York, 1995.

9. Swartz, D. L.; Odom, A. L., Effects of 5,5-substitution on dipyrrolylmethane ligand isomerization *Dalton Trans.* **2008,** 4254-4258.

10. Kegley, S. E.; Pinhas, A. R.; Collman, J. P., *Problems and solutions in organometallic chemistry* University Science Books: Mill Valley, Calif., 1986; p xii, 323 p.

11. Livingstone, D., *A Practical Guide to Scientific Data Analysis* Wiley: Hoboken, N.J, 2009.

12. Carlson, R.; Carlson, J. E., *Design and Optimization in Organic Synthesis* 2nd ed.; Elsevier,: Amsterdam ; San Diego, CA, 2005.

13. Draper, N. R.; Smith, H. E., *Applied regression analysis* 3rd ed.; Wiley: New York, 1998; p xvii, 706 p.

14. Box, G. E. P.; Cox, D. R., An analysis of transformations *J. Royal Stat. Soc. B* **1964,** *26*, 211-252.

15. Box, G. E. P.; Cox, D. R., An analysis of transformations revisited, rebutted *J. Am. Stat. Ass.* **1982,** *77*, 209-210.
